# Photoactivatable Fluorescent Dyes with Hydrophilic Caging Groups and Their Use in Multicolor Nanoscopy

**DOI:** 10.1021/jacs.1c09999

**Published:** 2021-10-29

**Authors:** Alexey N. Butkevich, Michael Weber, Angel R. Cereceda Delgado, Lynn M. Ostersehlt, Elisa D’Este, Stefan W. Hell

**Affiliations:** † Department of Optical Nanoscopy, Max Planck Institute for Medical Research, Jahnstrasse 29, 69120 Heidelberg, Germany; ∥ Department of NanoBiophotonics, Max Planck Institute for Biophysical Chemistry, Am Fassberg 11, 37077 Göttingen, Germany; § Optical Microscopy Facility, Max Planck Institute for Medical Research, Jahnstrasse 29, 69120 Heidelberg, Germany

## Abstract

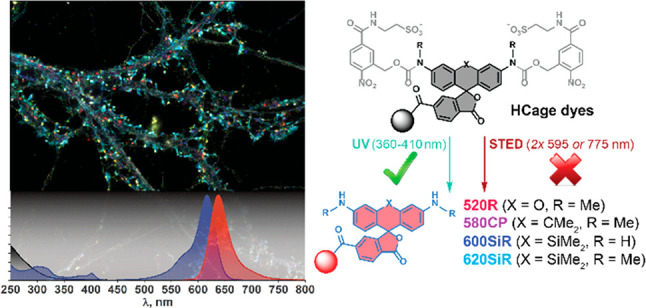

We propose a series of fluorescent dyes with hydrophilic carbamate caging groups that undergo rapid photoactivation under UV (≤400 nm) irradiation but do not undergo spurious two-photon activation with high-intensity (visible or infrared) light of about twice the wavelength. The caged fluorescent dyes and labels derived therefrom display high water solubility and convert upon photoactivation into validated super-resolution and live-cell-compatible fluorophores. In combination with popular fluorescent markers, multiple (up to six)-color images can be obtained with stimulated emission depletion nanoscopy. Moreover, individual fluorophores can be localized with precision <3 nm (standard deviation) using MINSTED and MINFLUX techniques.

Fluorescence microscopy has long been recognized as a method of choice for visualization of cellular structures and localization of biomolecules on a subcellular level. The advent of fluorescence nanoscopy (super-resolution microscopy), which overcomes the diffraction limit by differentiating between distinct molecular states of the emitters,^1^ necessitates the development of fluorescent dyes with tightly controlled photophysical properties. In particular, photoactivatable or photoswitchable fluorophores are known to fulfill the requirements of single-molecule imaging and nanoscopy techniques.^2^ These fluorophores undergo either a photochemical isomerization between two distinct forms, one fluorescent and the other non-fluorescent, or a photochemical reaction irreversibly converting a non-emitting precursor into a fluorescent dye.^3^ The precursor molecule usually represents a known fluorophore modified with a photocleavable protecting (so-called caging) group at one or more of the auxochromic residues, leading to disruption of charge delocalization across the rigid planar π-conjugated fluorophore core.^4^

In some exceptional cases, a caging modification involves the substitution with or introduction of only a few atoms into the molecule.^5^ However, the most robust and widely used 2-nitrobenzyl^6b^ and 4,5-dialkoxy-2-nitrobenzyloxycarbonyl (in particular nitroveratryloxycarbonyl, or NVOC)^6^ protecting groups add significantly to the bulkiness and hydrophobicity of the caged fluorescent label. This often leads to technical problems during labeling with caged dyes, such as poor aqueous solubility of molecular probes, aggregation, and precipitation of labeled antibodies,^7^ and may result in loss of affinity or induce degradation of labeled fusion proteins.^8^ Furthermore, the widely recommended diazoketone-caged triarylmethane dyes^9^ are poorly compatible with the transient high-intensity visible or NIR irradiation (e.g., with a 775 nm pulsed laser) required in stimulated emission depletion (STED) nanoscopy, due to their sensitivity to two-photon uncaging.^9b^

Recently, sulfonated rhodamine- and silicon-rhodamine-derived probes, internalizing upon conjugation to their molecular targets, have been described as cell-membrane-impermeant fluorescent substrates for SNAP-tag- and HaloTag-fused cell surface proteins.^10^ Here, following an entirely different approach, we propose the introduction of polar sulfonate groups onto the lipophilic 2-nitrobenzyl carbamate protecting groups, rendering the caged dyes highly water-soluble and allowing photocontrol over their membrane permeability. Following a preliminary screening of the substitution pattern of the hydrophilic caging groups, the molecules HCage 520 (**4aa**), HCage 580 (**4ba**), HCage 620 (**4ca**), and HCage 600 (**4cb**) (Figure 1) have been selected for the optimal combination of stability against two-photon activation with 595 and 775 nm STED light pulses and solubility in aqueous media without any addition of organic cosolvents. These target compounds have been prepared from known 6′-(*tert*-butoxycarbonyl)fluorescein, -carbofluorescein, and -silicofluorescein triflates^11^**1a**–**1c** and the corresponding carbamates **2a** and **2b** via a double Buchwald–Hartwig amidation catalyzed with a Pd-JackiePhos system^12^ under anhydrous conditions. The polar SO_3_H groups, anionic under physiological conditions and imparting solubility in water, were introduced into the intermediates **3aa–3cb** via basic hydrolysis of the esters and peptide coupling with taurine. The final deprotection of the 6′-carboxylate group offered the target caged dyes **4aa–4cb** suitable for conjugation.

**Figure 1:**
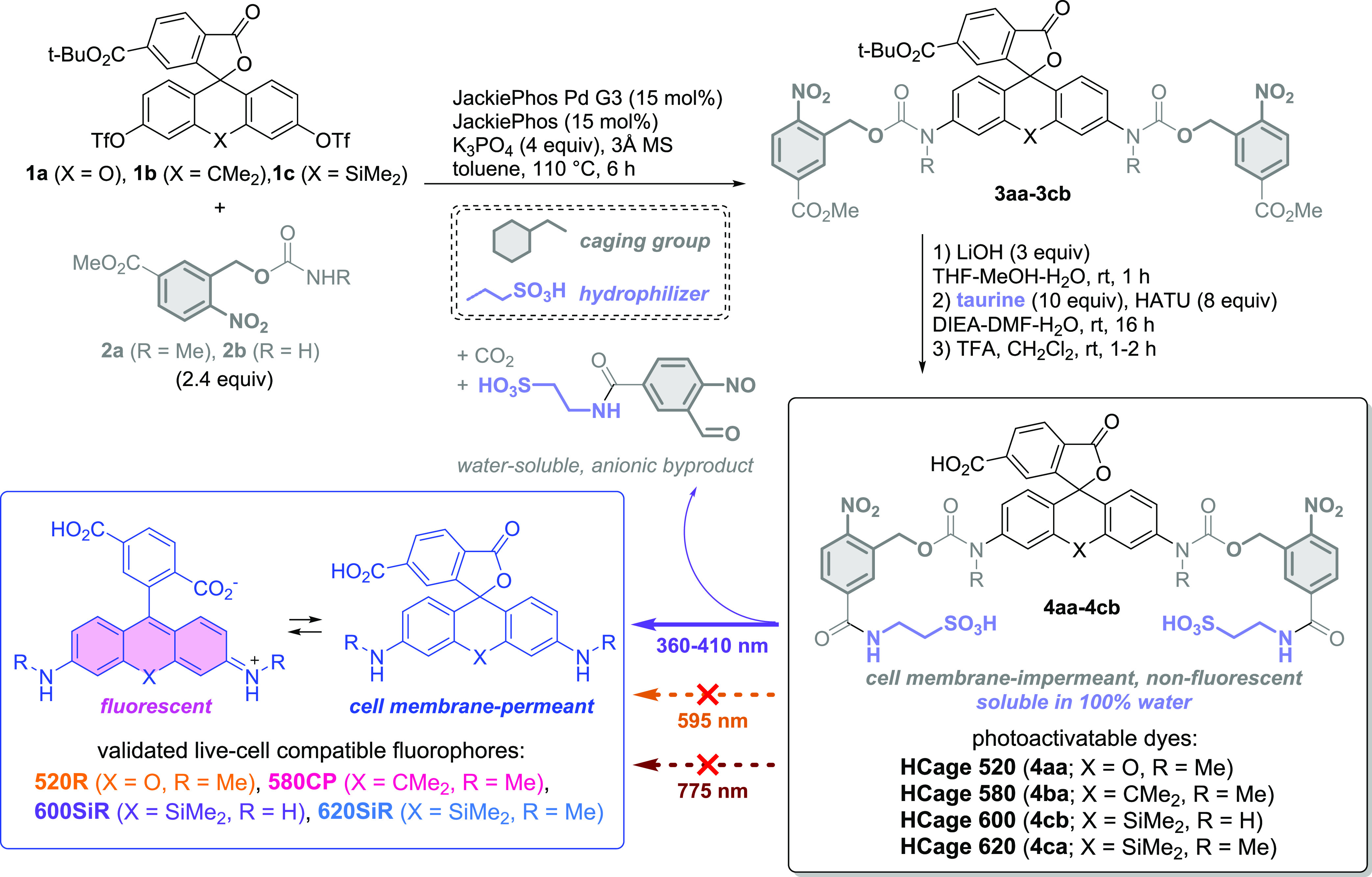
Synthesis of the photoactivatable triarylmethane dyes with hydrophilic caging groups (HCage dyes).

Upon photoactivation with UV light (400 nm LED, 355 or 405 nm laser sources), the caged dyes are cleanly converted (Figure S1) into the known cell-membrane-permeant and live-cell-compatible fluorescent dyes 520R, 580CP,^11b^ 620SiR,^13^ and its close analog 600SiR (Figure S2).

Initial evaluation of the HCage dyes has been performed by immunostaining of fixed and permeabilized U2OS cells with **4ba** (Figure S3) or **4ca**. Sample fluorescence was measured before and after a brief (2 s) activation with a 400 nm broadband LED. Under these conditions, rapid and complete uncaging of the conjugated dyes was observed, producing high-contrast images (with <0.5% fluorescence signal detected before photoactivation relative to the final image). With usual hydrophobic carbamate caging groups, a straightforward labeling procedure was previously reported to be impossible due to extensive aggregation of labeled IgG,^7^ and sample preparation required development of elaborate conjugation and purification routines. The resistance of the labeled structures against inadvertent two-photon activation with high-intensity STED light (∼430 MW/cm^2^ at the sample) was confirmed for **4ba**, **4ca**, and **4cb** (Figure S4).

With demonstrated robustness of the hydrophilic caging groups against two-photon activation, we explored the possibility of using HCage dyes for multiplexing experiments (Figure 2, Figure S5). Indeed, dyes customarily used for STED and HCage labels can be differentiated within the same spectral window based on sequential imaging and bleaching of the former and photoactivation of the latter. As a proof of principle, we have labeled six different structures in mature hippocampal neurons with two spectrally distinguishable caged dyes (HCage 620 **4ca** and HCage 580 **4ba**), a corresponding spectrally similar pair of popular 775 nm STED dyes (Abberior Star 635P and Star 580), a 595 nm STED dye excitable at 485 nm (Alexa Fluor 488), and a fluorescent dye excitable at 405 nm (Alexa Fluor 405). The imaging (Figure 2b) was performed by recording sequentially: a two-color 775 nm STED image with Star dyes (step I), a 595 nm STED image with concomitant bleaching of Star dyes with a 595 nm laser (step II), a confocal image with Alexa Fluor 405 followed by the activation of HCage dyes with a 405 nm laser (step III), and finally the second two-color 775 nm STED image with the uncaged HCage dyes (step IV). The overlaid six-color data image permits localization of the selected synaptic proteins below the diffraction limit in the context of the cytoskeleton. For example, we could visualize a dendritic spine (actin) with two different postsynaptic contacts (Bassoon, presynapse): one inhibitory (Gephyrin, postsynapse) and one excitatory (PSD95, postsynapse) (Figure 2c). Gaining detailed information on the context and synaptic status is especially relevant in the studies of neuronal plasticity.^14^ HCage dye-based probes therefore represent a new tool for multiplexed imaging as they allow for standard sample preparation procedures, are compatible with commercial STED microscopes, and do not require data post-processing such as spectral unmixing.^14a^

**Figure 2:**
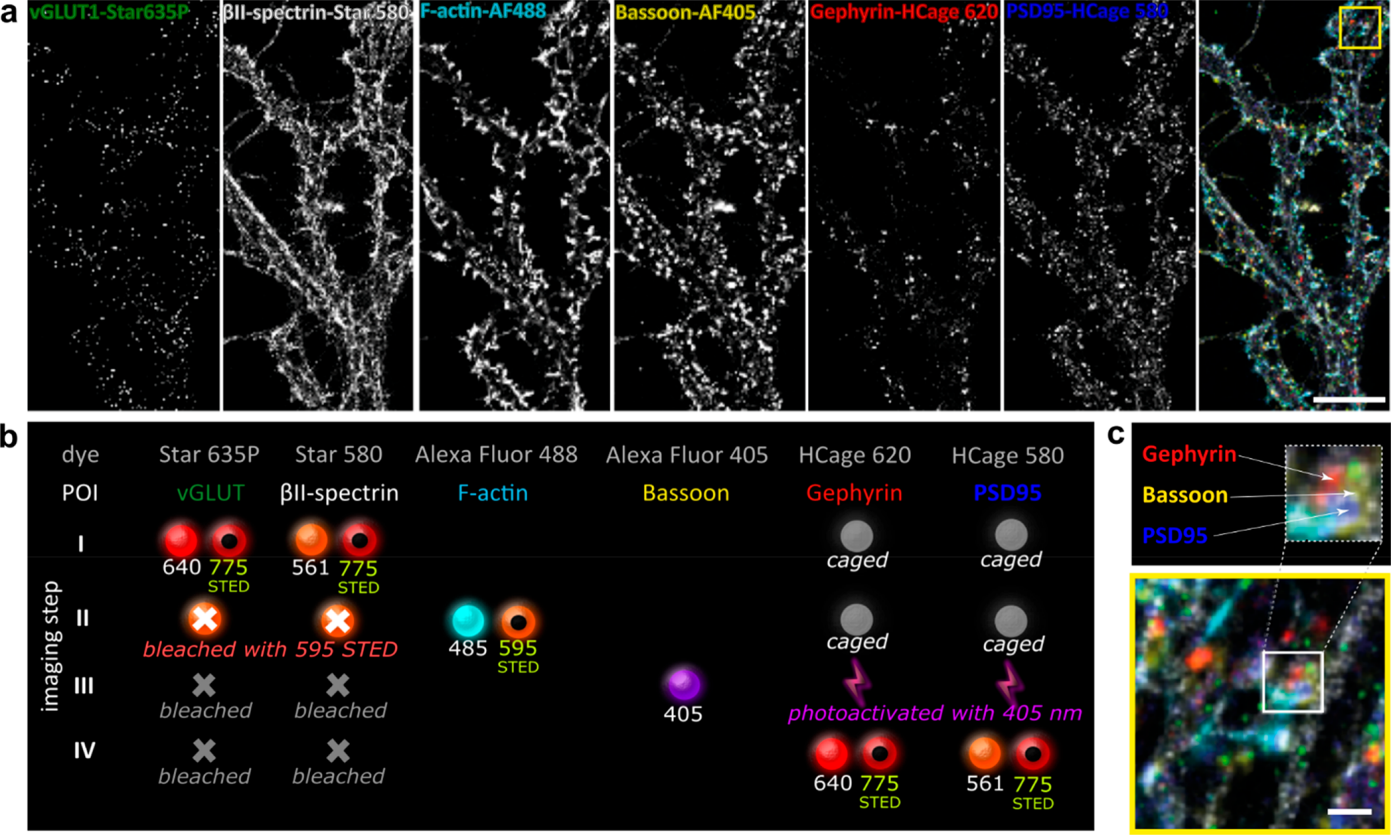
Six-color fluorescence microscopy (5× STED + 1× confocal) in fixed rat hippocampal primary neurons with the dyes of the present study (HCage 580, HCage 620) employed for color multiplexing. (a) Individual color channels and six-color overlay image. (b) Imaging sequence (I → IV, top to bottom) depicting fluorescence excitation (solid spheres) and STED (donut shape) laser lines used (wavelengths in nm), bleached fluorophores (gray crosses), caged fluorophores (gray disks) unaffected by excitation and STED light, and photoactivated (uncaged) fluorophores (lightning shapes). (c) Expanded area of the multicolor image (yellow box in (a)) showing inhibitory (Gephyrin, red) and excitatory (PSD95, blue) postsynaptic sites with the presynaptic counterpart (Bassoon, yellow) contacting a single dendritic spine. Scale bars: 10 μm (a) and 1 μm (c). Image represents a selected area of the image shown in Figure S5.

The intact cell membrane impermeability for the photoactivatable HCage dyes has been verified by incubating the U2OS cells stably expressing vimentin-HaloTag fusion protein^13^ with 2 μM **4ca-Halo** ligand in HDMEM buffer for 1 h (Figure S6). When the media had been exchanged to a dye-free buffer, only very minor staining was observed, likely due to endocytic uptake pathways rarely reported for negatively charged fluorophores.^15^ In the dye-containing media, the bright and selective staining of vimentin filaments was observed upon 2 s of wide-field UV irradiation, with the fluorescence intensity peaking after 60 s (four confocal frames with 900 μm^2^ imaging area) due to diffusion of the uncaged 620SiR-Halo, and was followed by slow bleaching of the labeled structure (Figure S7). The dynamics of vimentin filaments were also observed (Video S1). A similar observation was made for **4ba-Halo** (500 nM; Figure S8 and Video S2) and confocal UV activation, revealing complete labeling of the target structure within 5–6 min. The labeled structure could be imaged with sub-diffraction resolution (Figure S9). The specificity of the staining was confirmed by an *in situ* uncaging experiment with fixed U2OS cells stably expressing vimentin-HaloTag labeled with HCage 620 (as **4ca-Halo**) and indirect immunostaining of vimentin (Figure S10).

To demonstrate the potential of spatially controlled activation of caged fluorogenic labels directly under microscopic conditions, U2OS cells transfected with Tomm20-HT7-T2A-EGFP plasmid and mounted in a live imaging chamber in medium containing 500 nM of **4ba-Halo** ligand were irradiated at several 4 μm^2^ sized loci in close proximity to the cell membrane, and the development of target labeling was monitored over multiple frames (Figure S11 and Video S3). High fluorogenicity of the **4ba-Halo** uncaging product 580CP-Halo,^11b^ along with its rapid binding kinetics with HaloTag protein (with *k*_app_ estimated at 4.01 ± 0.31 × 10^7^ M^–1^ s^–1^ for HT7 version, see Figure S12),^8b^ provides a realistic background-free dynamic visualization of live-cell labeling. We can therefore recommend this caged HaloTag substrate for real-time observation experiments such as comparing the cellular uptake of the fluorescent probe under varying conditions, or for tagging small molecules of biological relevance and targeting them to the HaloTag-fused proteins of interest within the living cells. The required spatial and temporal control over the generation of a cell-permeant label can be conveniently achieved with brief focused UV irradiation of moderate (∼12 MW/cm^2^) intensity.

The low fluorescence background in samples labeled with caged dyes **4aa–4cb** and their selective photoactivation with UV light prompted us to evaluate their performance in recently proposed advanced fluorescence nanoscopy methods, called MINFLUX^16^ and MINSTED.^17^ For benchmarking purposes, microtubules in glutaraldehyde-fixed U2OS cells were immunostained with Abberior Star RED (KK114^18^), a widely accepted photostable and highly water-soluble STED dye. The attainable resolution of both confocal and STED images (Figure 3a,b) could then be directly compared with the image consisting of overlaid single-molecule localizations of individual secondary antibodies labeled with HCage 620 (Figure 3c,d). The hollow tubular shape of an individual microtubule becomes evident in the *y*-integrated cross-section (*x-z* projection, Figure 3e) of the 3D MINFLUX image (for histograms including all localization, see Figure S13) and in the *y-z*-integrated cross-section (Figure 3f, including all localization).^16b^ The combined use of HCage 620 dye and the MINFLUX method enabled a localization precision of 2.4 nm (along the *x*-axis) and 2.7 nm (along the *y*-axis), estimated by a 2D-Gaussian fit on the spread of all localizations centered around their mean emitter positions (Figure 3g). Averaging the standard deviation of the localizations around their mean emitter position led to comparable results, with a median localization precision of 3.0, 4.0, and 3.1 nm along the *x*-, *y*-, and *z*-axes, respectively.

**Figure 3:**
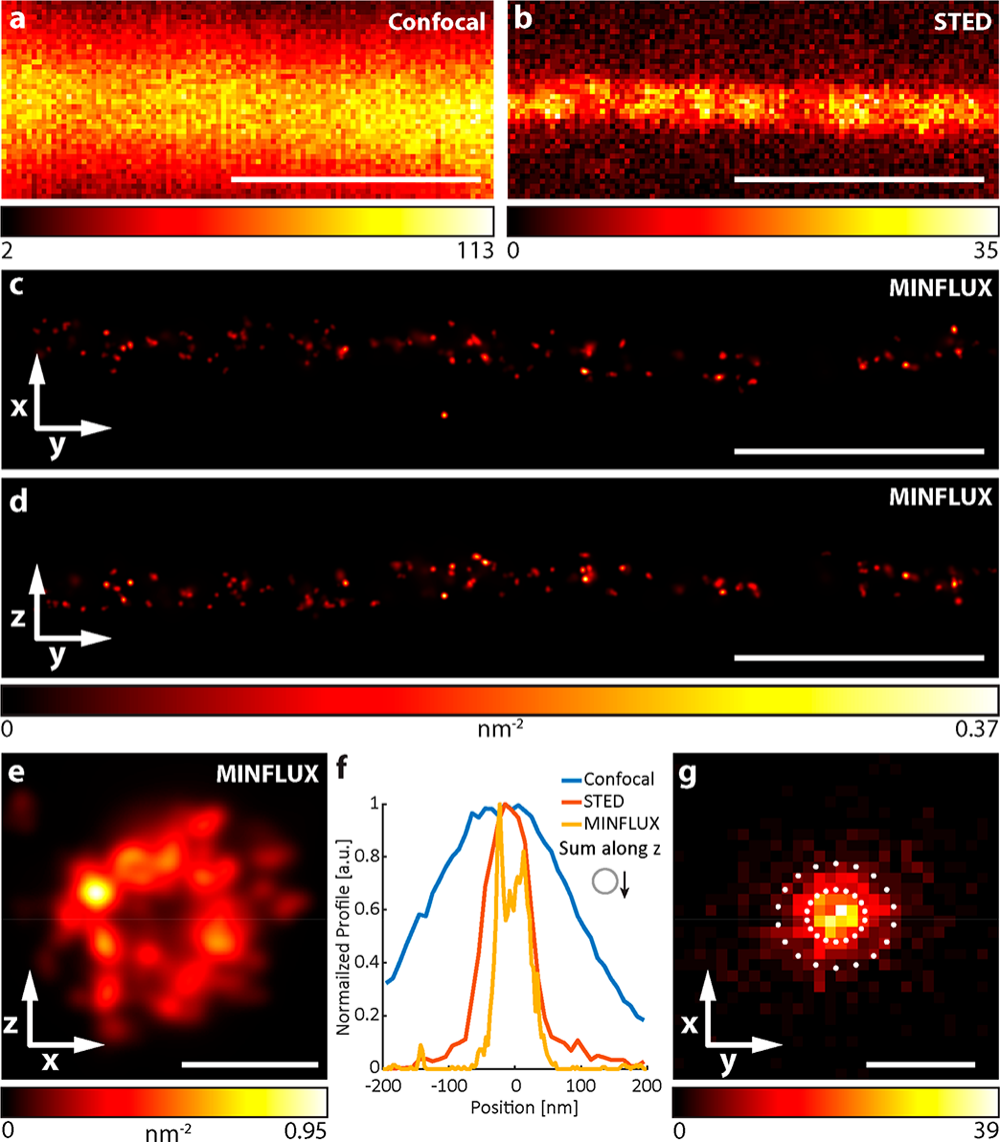
Confocal, STED, and MINFLUX images of microtubules from fixed immunolabeled U2OS cells. (a) Confocal and (b) 2D STED images of a single microtubule (α-tubulin labeled with primary antibody and secondary antibody with Abberior Star RED). (c, d) Rendered 3D MINFLUX side projections and (e) projection along the estimated tubule path, showing individual uncaged fluorophores with at least four localizations (α-tubulin labeled with primary antibody and secondary antibody with HCage 620). (f) Cross-section histogram along the *x*-axis for the different imaging methods (MINFLUX including emitters with less localizations). (g) Histogram of the localization spread around their emitter centers, considering emitters with at least four localizations (with 2000 photons each) and Gaussian-fitted localization precision with 1σ and 2σ indicated by circles (σ^*x*^: 2.4 nm, σ^*y*^: 2.7 nm). Scale bars: 500 nm (a–d), 50 nm (e), and 10 nm (g).

The high resistance of HCage 620-based probes against activation with STED laser and good photostability of 620SiR photoproduct allowed sparse activation of diverse caged ligands in MINSTED nanoscopy.^17^ We demonstrated effective MINSTED imaging of antibody-labeled caveolin clusters (Figure 4a, labeling with NHS ester), HaloTag- and SNAP-tag-labeled nucleoporins (Nup96 and Nup107, respectively, Figure 4b,c), and Nup107-mEGFP with anti-GFP nanobody (V_H_H heavy-chain IgG camelid antibody fragments) labeled with maleimide (Figure 4d). The localization precision for individual fluorophore emitters, singled out with MINSTED, was estimated at 2.6–3.5 nm (single standard deviation) for different ligands (Figure S15).

**Figure 4:**
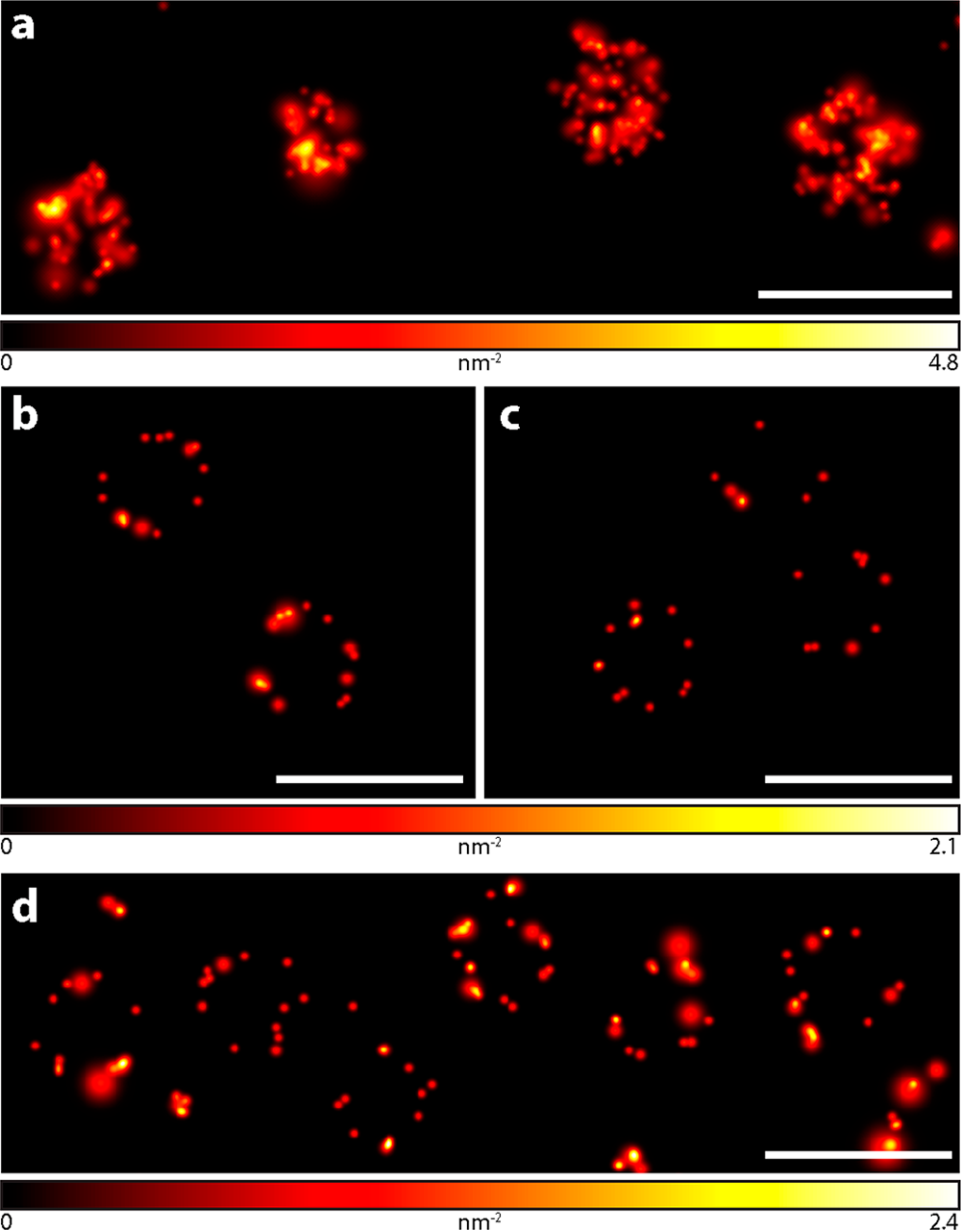
MINSTED images of macromolecular assemblies recorded in fixed U2OS cells. (a) Caveolin-1 labeled with primary and secondary antibodies with HCage 620. (b, c) Nup96 endogenously tagged with SNAP-tag (b) or HaloTag (c) and labeled with HCage 620-BG or -Halo ligand, respectively. (d) MINSTED images of fixed HeLa cells endogenously expressing Nup107-mEGFP and labeled with single-domain anti-GFP nanobody and HCage 620-maleimide (for full images, see Figure S14). Scale bars: 200 nm.

In conclusion, the proposed hydrophilic caged versions HCage 520, 580, 600, and 620 (**4aa–4cb**) of the established live-cell-compatible triarylmethane fluorophores 520R, 580CP, 600SiR, and 620SiR can be recommended for imaging in fixed (e.g., with immunostaining) and living cells (following on-demand uncaging to cell-permeant labels in the media). These caged dyes are applicable across most leading fluorescence nanoscopy modalities (STED, PALM, MINFLUX, and MINSTED). The precise spatiotemporal control over their photoactivation provides additional avenues for real-time monitoring of localized uptake of membrane-permeant fluorescent and fluorogenic ligands, such as 580CP-Halo^11b^ or 620SiR-SNAP,^13^ as well as fluorophore-tagged small molecules. In particular, the ability to sparsely activate and precisely localize individual labeled biomolecules in time and space brings us closer to the ultimate goal of understanding the biochemical processes inside a living cell on a molecular level.

## Supporting Information Available

Synthetic procedures and characterizations of the compounds; description of sample preparation, labeling, imaging, and image processing, including Figures S1–S16 and Tables S1 and S2; and NMR spectra (PDF)
Supplementary Video S1: confocal and STED time-lapse video of living U2OS cells stably expressing vimentin-HaloTag fusion protein labeled with photoactivated HCage 620-Halo (MP4)
Supplementary Video S2: photoactivation of HCage 580-Halo in the media and real-time observation of vimentin labeling in living U2OS cells stably expressing vimentin-HaloTag fusion protein (AVI)
Supplementary Video S3: localized uncaging of HCage 580-Halo and real-time observation of its uptake and Tomm20 labeling in living U2OS cells transfected with Tomm20-HT7-T2A-EGFP plasmid (AVI)

## Supplementary Material

ja1c09999_si_001.mp4

ja1c09999_si_002.avi

ja1c09999_si_003.avi
