# Supplementary material for: Photoactivatable Fluorescent Dyes with Hydrophilic Caging Groups and Their Use in Multicolor Nanoscopy
Source: J Am Chem Soc. 2021 Oct 29;143(44):18388–93. doi: 10.1021/jacs.1c09999 (PMC8587603; doi:10.1021/jacs.1c09999)
Supplement: Supplementary file 1 — ja1c09999_si_004.pdf [file ja1c09999_si_004.pdf]

Supplementary information

Photoactivatable fluorescent dyes with hydrophilic caging groups and their use in multicolor nanoscopy

Alexey N. Butkevich[‡,*,a], Michael Weber[‡,b], Angel R. Cereceda Delgado[a,b], Lynn M. Ostersehlt[b], Elisa D'Este[c], Stefan W. Hell[*,a,b]

[a] Department of Optical Nanoscopy, Max Planck Institute for Medical Research, Jahnstrasse 29, 69120 Heidelberg, Germany

[b] Department of NanoBiophotonics, Max Planck Institute for Biophysical Chemistry, Am Fassberg 11, 37077 Göttingen, Germany

[c] Optical Microscopy Facility, Max Planck Institute for Medical Research, Jahnstrasse 29, 69120 Heidelberg, Germany

[‡] These authors contributed equally.

*Corresponding authors' e-mails: alexey.butkevich@mr.mpg.de and stefan.hell@mpibpc.mpg.de.

Table of Contents

Supplementary Figures 5
Figure S1 5
Figure S2 6
Figure S3 7
Figure S4 8
Figure S5 9
Figure S6 10
Figure S7 11
Figure S8 12
Figure S9 13
Figure S10 14
Figure S11 15
Figure S12 16
Figure S13 17
Figure S14 18
Figure S15 19
Figure S16 20
Supplementary Methods..21
Manipulation of cells 21
Antibody coupling 21
Nanobody coupling..21
Cell culture and sample preparation 21
Neuron culture and sample preparation..23
Cell membrane permeability experiments..24
Imaging and data processing..25
Imaging 25
Table S1 26
Table S2 27
Image processing 28
General experimental information and synthesis 29
Thin layer chromatography..29
Preparative flash column chromatography..29

High-Performance Liquid Chromatography (HPLC) and Mass Spectrometry (MS) 29
Optical spectroscopy 30
HaloTag7 labeling kinetics 31
NMR spectra 32
Preparation of the starting materials 33
5-(Methoxycarbonyl)-2-nitrobenzyl 1*H*-imidazole-1-carboxylate (**S1**) 33
Methyl 3-(methylcarbamoyloxy)methyl-4-nitrobenzoate (**2a**) 33
Methyl 3-(carbamoyloxy)methyl-4-nitrobenzoate (**2b**) 34
General synthetic procedures for the preparation of caged fluorescent dyes 35
General procedure 1 (Buchwald-Hartwig amidation): compound **3aa** 35
General procedure 2 (hydrolysis of methyl ester): compound **3aa-OH** 36
General procedure 3 (amide coupling): compound **4a** 37
General procedure 4 (hydrolysis of *tert*-butyl ester): caged dye **4aa** (HCage 520) 38
Compound **3ba** 39
Compound **3ba-OH** 40
Compound **3ba-SO_3H** 41
Caged dye **4ba** (HCage 580) 42
Compound **3ca** 43
Compound **3ca-OH** 44
Compound **3ca-SO_3H** 45
Caged dye **4ca** (HCage 620) 46
Compound **3cb** 47
Compound **3cb-OH** 48
Compound **3cb-SO_3H** 49
Caged dye **4cb** 50
Synthesis of photoactivatable labels 51
HCage 520 NHS ester (**4aa-NHS**) 51
HCage 580 NHS ester (**4ba-NHS**) 52
HCage 600 NHS ester (**4cb-NHS**) 53
HCage 620 NHS ester (**4ca-NHS**) 54

HCage 620 maleimide (**4ca-maleimide**) 55
HCage 600 HaloTag ligand (**4cb-Halo**) 56
HCage 620 HaloTag ligand (**4ca-Halo**) 57
HCage 620 SNAP-tag ligand (**4ca-BG**) 58
Preparation of 600SiR dye and 600SiR-Halo ligand 59
Compound **S2** 59
600SiR dye 60
600SiR-Halo ligand 61
Supplementary references 62
NMR spectra 64

Supplementary Figures

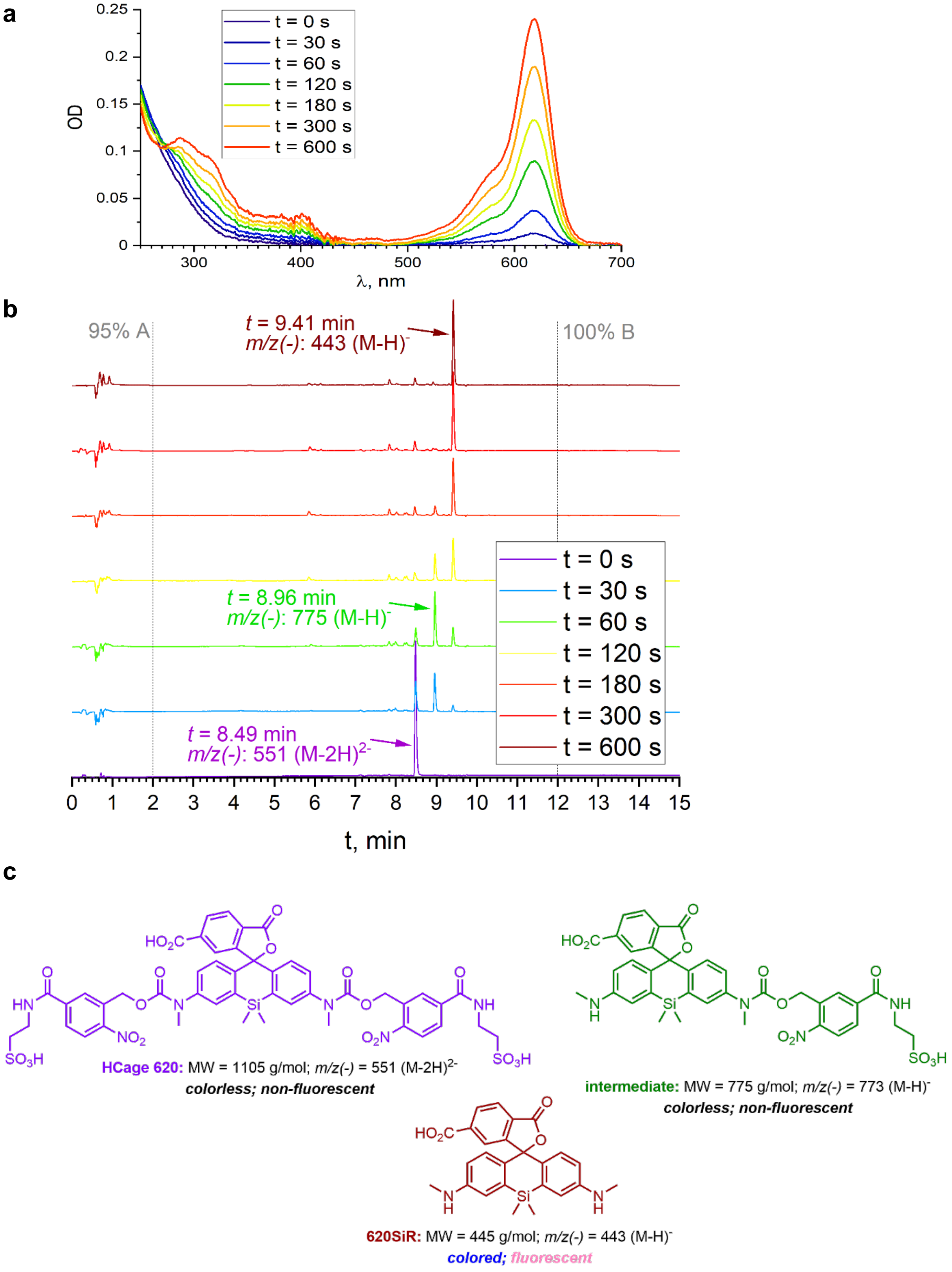

Figure S1. (**a**) Absorption spectra and (**b**) HPLC-MS analysis data (A: 50 mM ammonium formate, B: acetonitrile; detection at 254 nm) of the solution of HCage 620 (5 µM in 100 mM phosphate buffer, pH 7) irradiated at 365 nm for the indicated time (0…600 s). (**c**) Chemical structures of the starting material and the photoproducts.

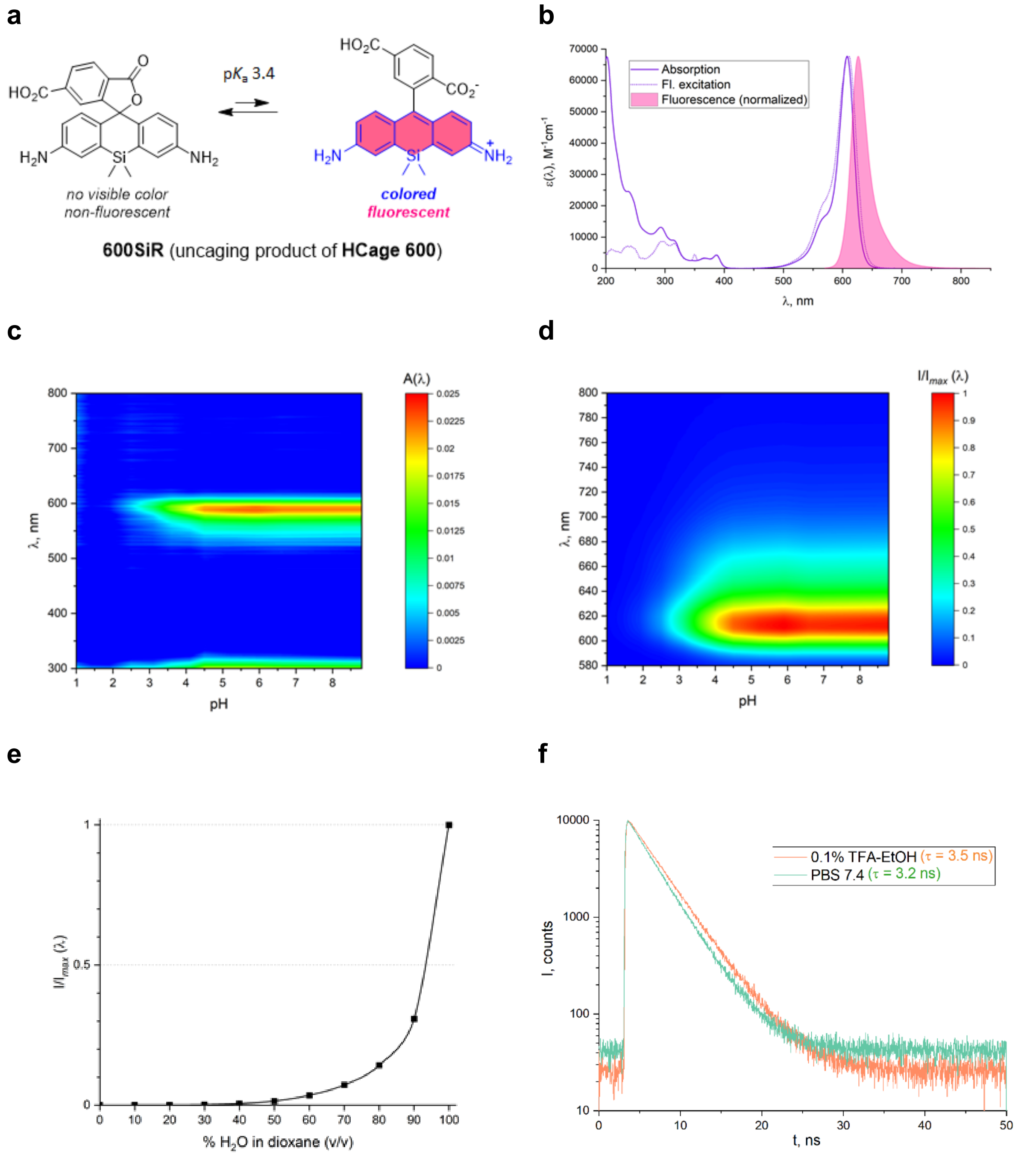

Figure S2. (**a**) Spirolactone-zwitterion equilibrium of 600SiR; (**b**) absorption, fluorescence excitation and fluorescence emission spectra of 600SiR in 0.1% (v/v) TFA – ethanol; (**c,d**): absorption (**c**) and fluorescence emission (**d**) spectra of 600SiR in 100 mM phosphate buffer (pH 1.0 … 8.8, + 1% (v/v) DMSO); (**e**) fluorescence emission intensity (at λ_{max} = 612 nm, normalized) of 600SiR in dioxane – H_2O mixtures (containing 1% (v/v) DMSO) with varying content of H_2O; (**f**) fluorescence decay of 600SiR in 0.1% (v/v) TFA – ethanol and PBS 7.4 solutions.

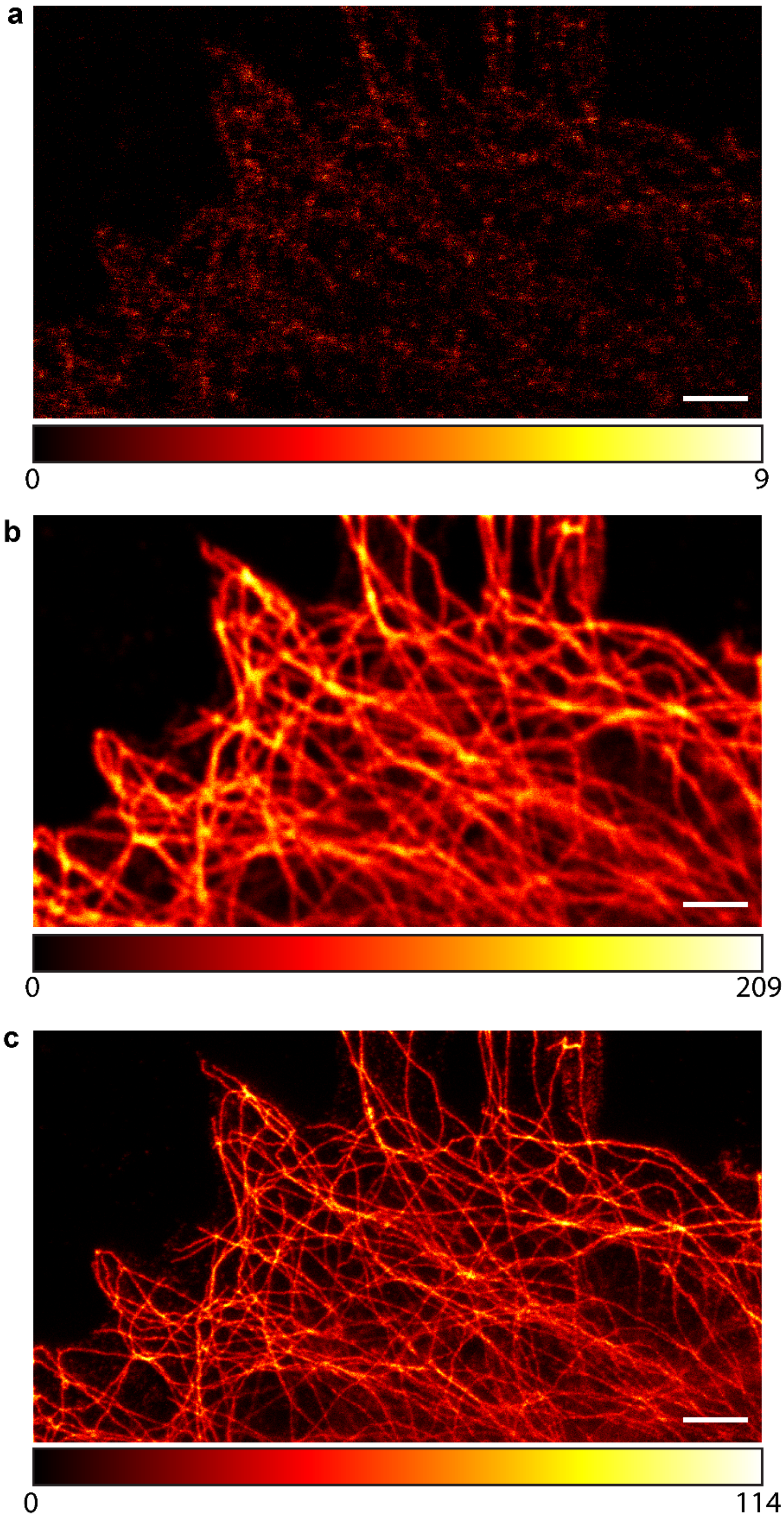

Figure S3. Images of ice-cold methanol fixed U2OS cells stained with a primary rabbit anti-α-tubulin anitibody (Abcam: ab18251) and secondary goat anti-rabbit (Dianova: 111-005-003) antibody conjugated to HCage 580 (**4ba**): **a,b** –– confocal before (**a**, background) and after UV activation with a broadband 400 nm LED (**b**); **c** – STED (775 nm) after UV activation with a broadband 400 nm LED. Scale bar: 2 μm.

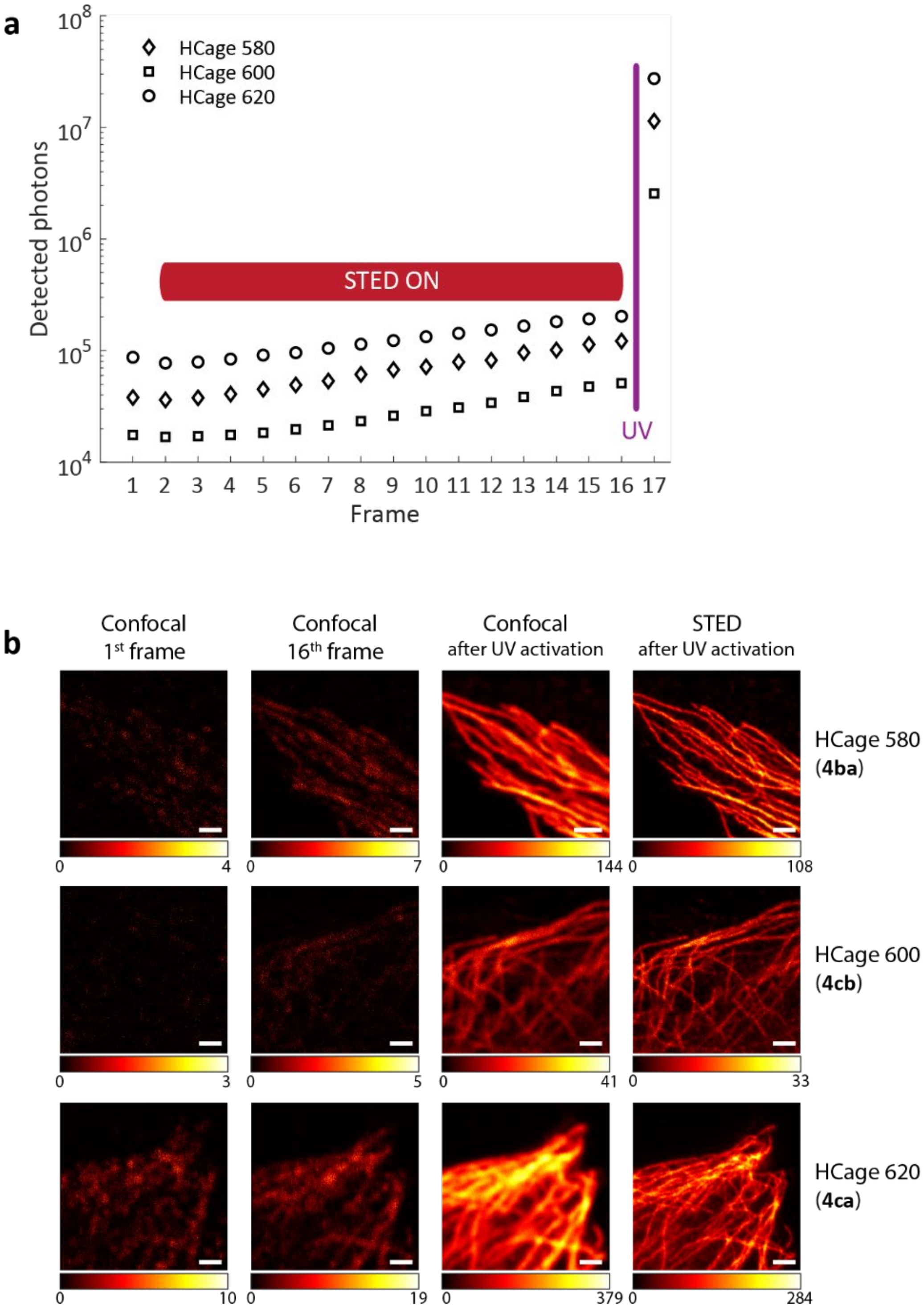

Figure S4. (**a**) Signal of consecutive confocal frames with parallel illumination with STED light (~430 MW/cm², frames 2-16) and after wide-field activation with UV light (frame 17, 6 W/cm²). Signal of the first frame (1) relative to the last frame (17): 0.32% (HCage 620), 0.33% (HCage 580) and 0.69% (HCage 600). (**b**) Background (before UV and after two-photon activation by STED) and confocal and STED images after UV activation using the same STED intensity, demonstrating lack of two-photon activation with the 775 nm STED beam and low initial signal. Scale bar: 1 µm.

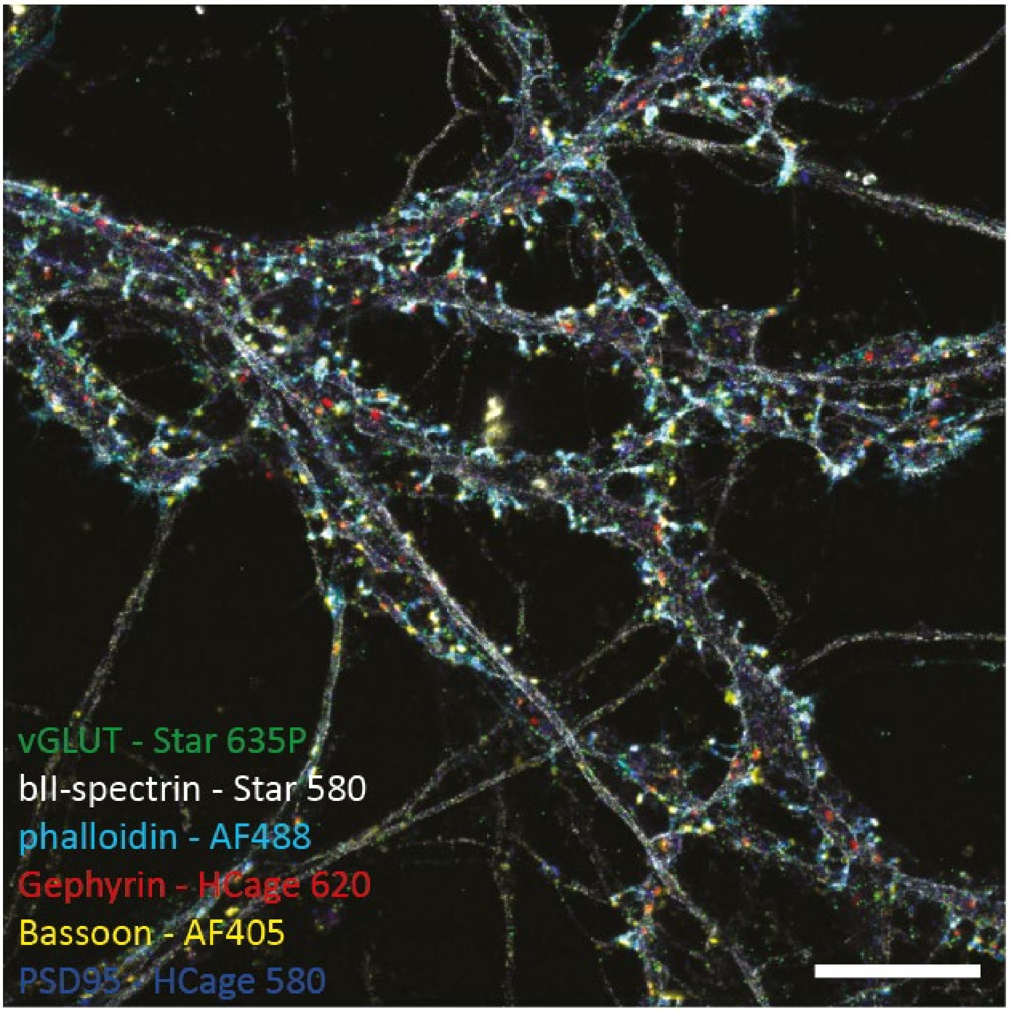

Figure S5. Full field of view (60 x 60 µm, pixel size 30 nm) for the image shown in Figure 2. Six color overlay image of vGLUT1 (Abberior STAR 635P, green), βII-spectrin (Abberior STAR 580, white), phalloidin (Alexa Fluor 488, cyan), Bassoon (Alexa Fluor 405, yellow, confocal), gephyrin (HCage 620, red), and PSD95 (HCage 580, blue). Scale bar: 10 µm. Total image acquisition time, including the corresponding confocal channels: ~ 55 min. Image acquisition parameters are listed in **Table S1**.

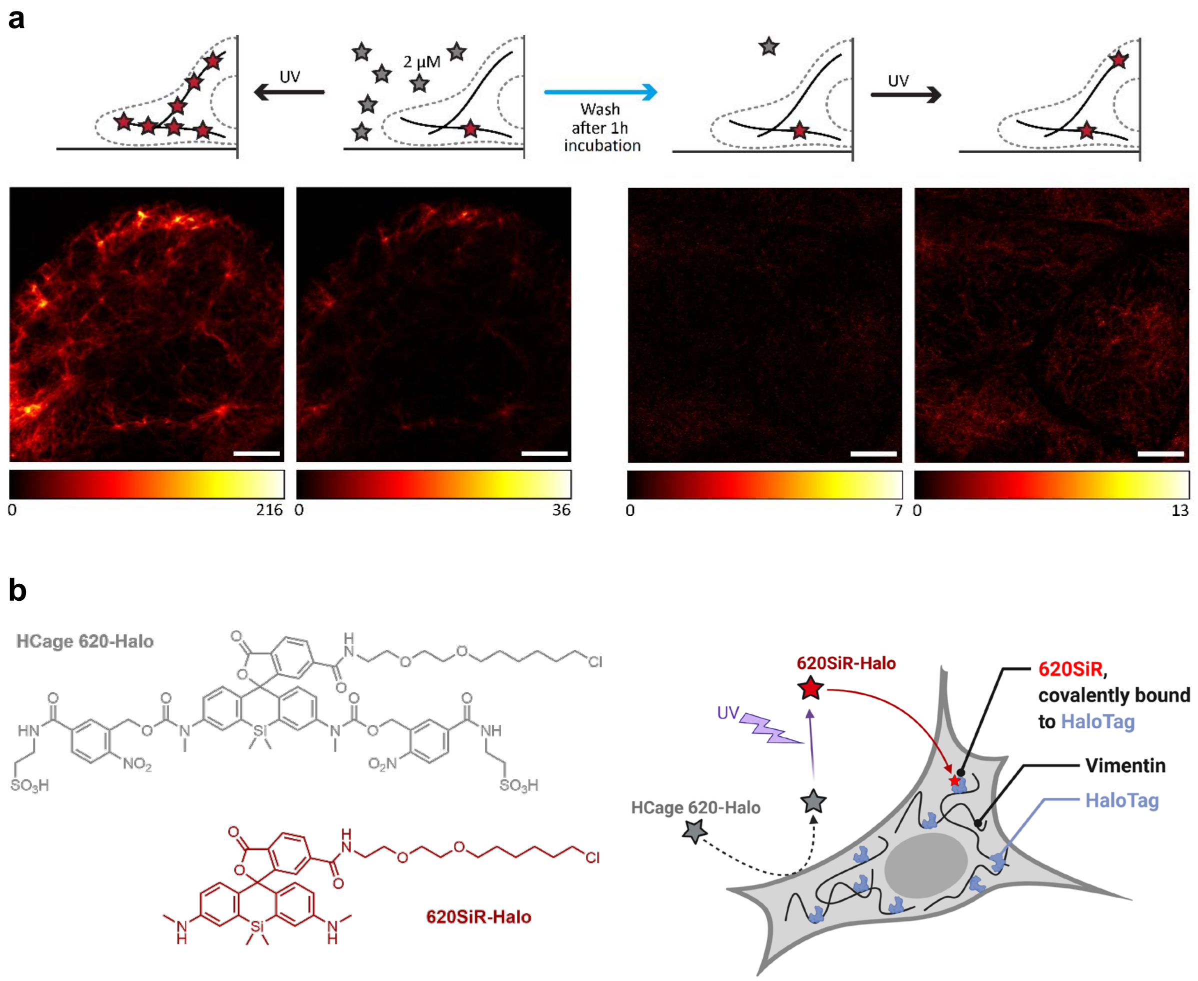

Figure S6. (**a**) Confocal images of living U2OS cells stably expressing vimentin-Halo tag fusion protein in HDMEM buffer with 2 μM HCage 620-Halo (**4ca-Halo**) before and 20 s after wide-field UV activation (left pair of images) and at a new area after 1 h of incubation and media exchange to HDMEM buffer without dye before and 20 s after UV activation (right pair of images). Scale bar: 5 μm. (**b**) Structures of the caged HCage 620-Halo and uncaged SiR620-Halo labels and a cartoon explaining UV-controlled switch of cell membrane permeability and labeling (figure created with BioRender.com).

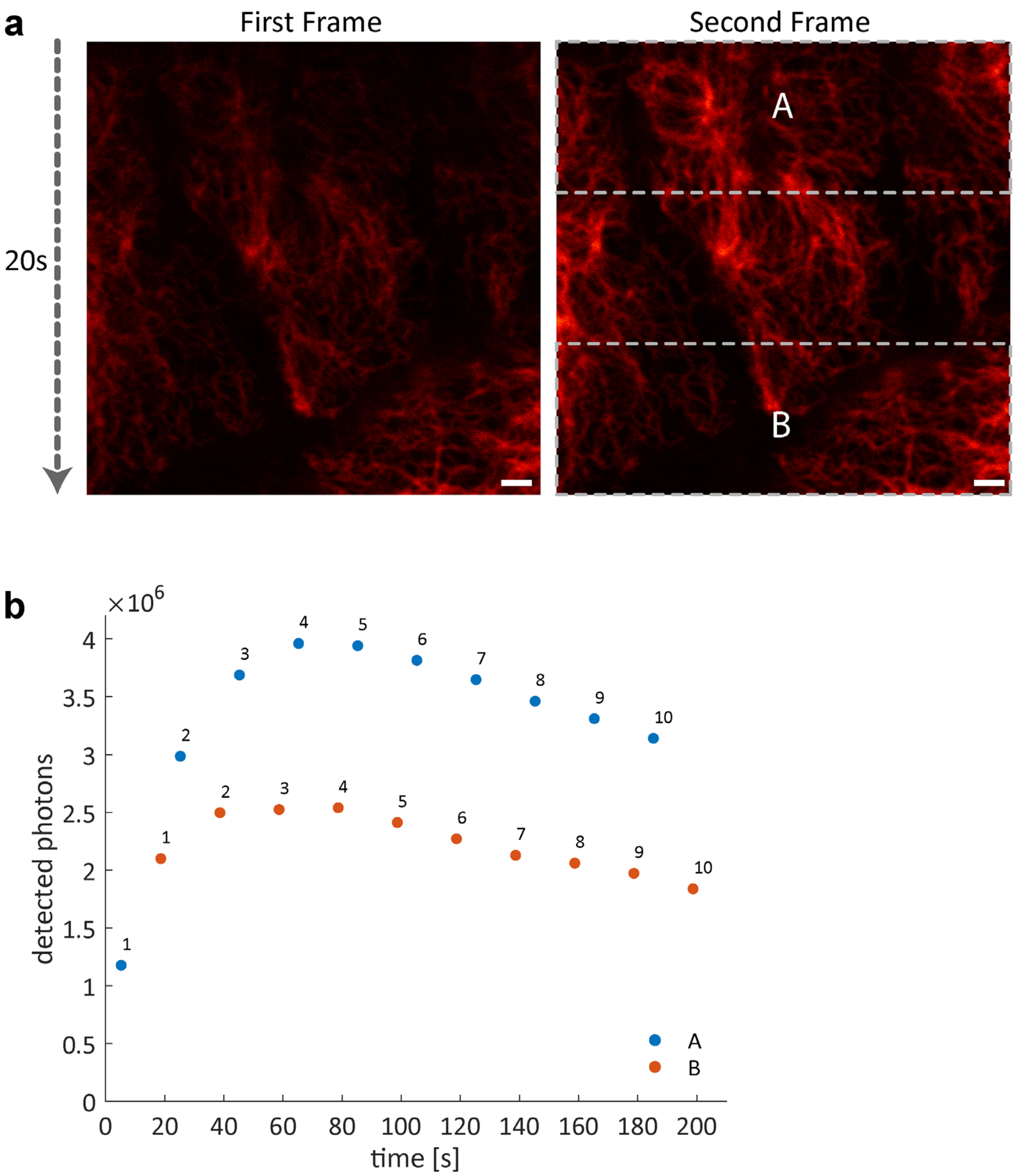

Figure S7. (**a**) First and second confocal frames (20 seconds per frame) of the supplementary video (**Video S1**) showing living U2OS cells stably expressing vimentin-HaloTag fusion protein, recorded in HDMEM buffer with 2 μM HCage 620-Halo (**4ca-Halo**) immediately after 2 seconds of widefield UV activation. Scale bar: 2 μm. (**b**) Accumulated signal evolution with time after UV activation of the upper (A) and lower third (B) acquisition. The respective frame number is indicated at each data point. The diffusion of the uncaged label and complete labeling of the target structure is complete within 40-60 s (from the frame 4 on, only slow bleaching of the labeled structure is observed), accounting for the time shift of the maximum brightness between A and B parts of the image.

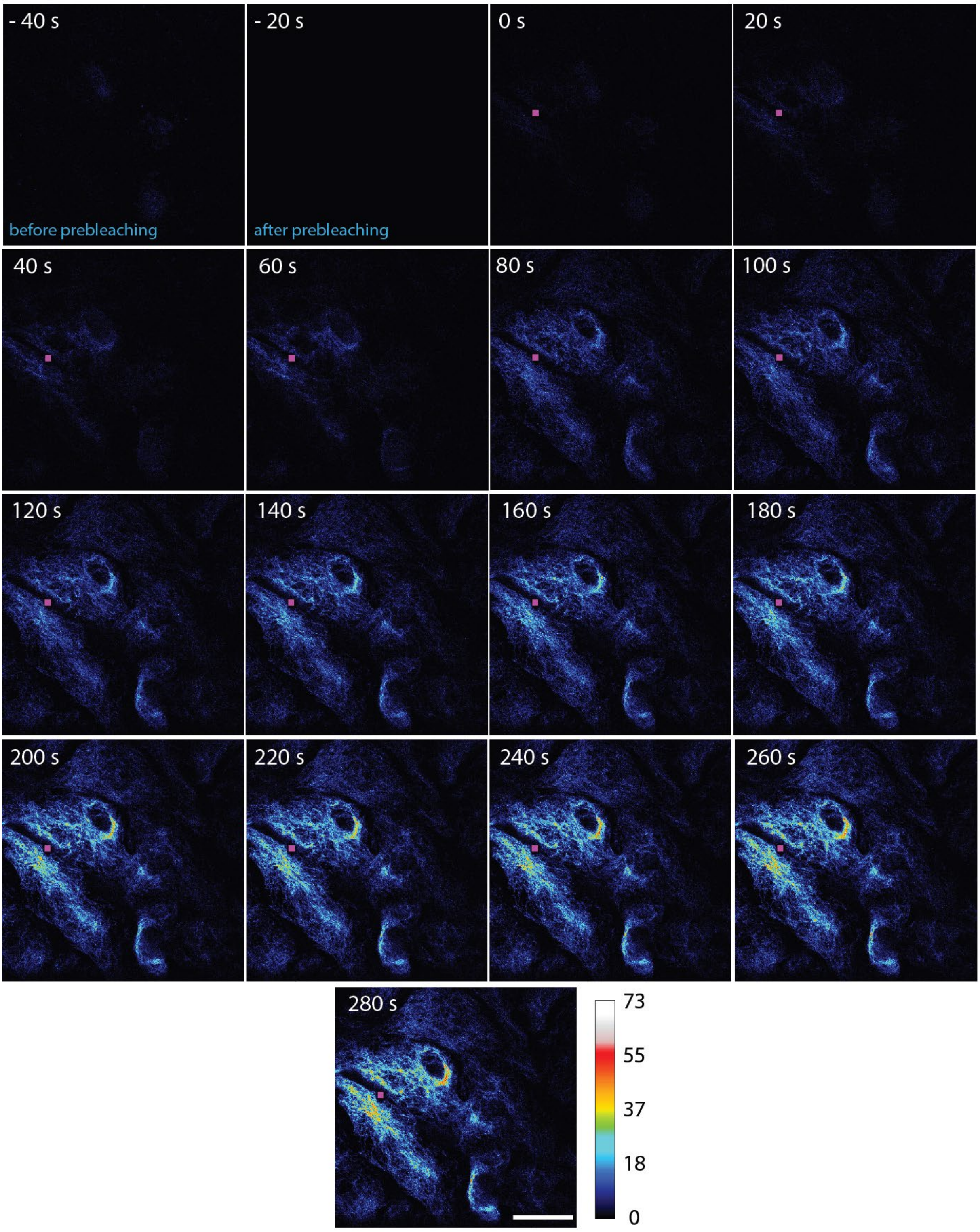

Figure S8. Confocal images of living U2OS cells stably expressing vimentin-HaloTag fusion protein, recorded in DMEM buffer (+penicillin/streptomycin, +glutamine) with 0.5 μM HCage 580-Halo (**4ba-Halo**) before and after prebleaching step (with 595 nm STED light), and following repeated cycles of activation with a 405 nm laser (12 MW/cm^2, 200 pulses with 10 μs dwell time on a 2×2 $μm^2$ region of interest outside the cells: ■) every 20 s. Excitation: 561 nm, detection: 590-630 nm. Scale bar: 200 μm.

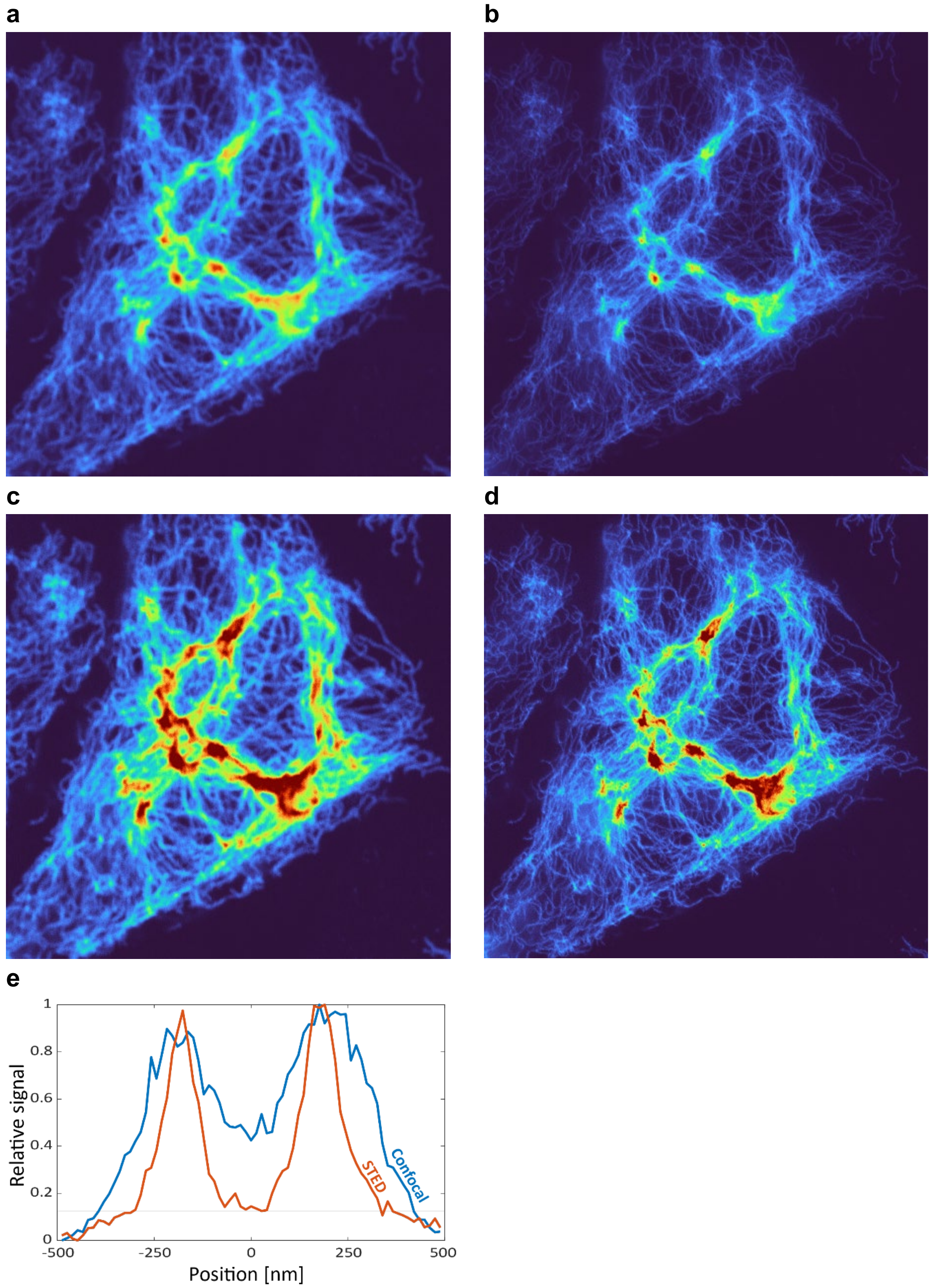

Figure S9. Confocal (**a**) and STED (**b**) image of U2OS cells stably expressing vimentin-HaloTag fusion protein recorded in HDMEM buffer with 2 μM HCage 620-Halo (**4ca-Halo**) ligand 20 s after widefield UV activation. Image size 30×30 μm. (**c,d**) Same images as (**a,b**) saturated to increase visibility. (**e**) Sample fluorescence intensity profiles across individual vimentin filaments.

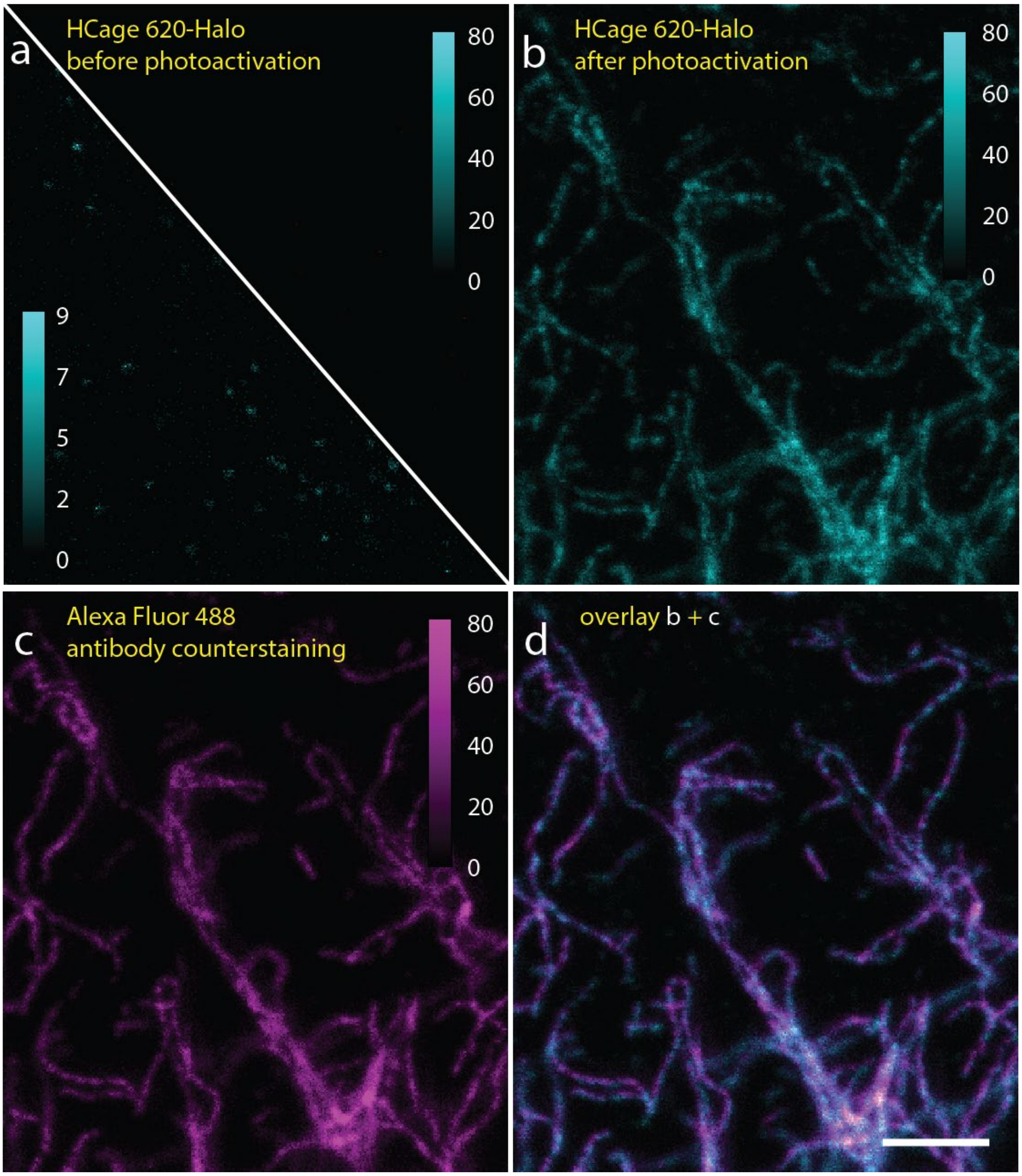

Figure S10. STED images of fixed U2OS cells stably expressing vimentin-HaloTag fusion protein labeled with HCage 620-Halo (**4ca-Halo**) before (**a**) and after (**b**) photoactivation with a 405 nm laser. Panel (**c**) shows a counterstaining with a primary anti-vimentin and secondary Alexa Fluor 488 antibody to visualize vimentin before photoactivation and confirm the colocalization with **4ca-Halo** (**d**). Samples were mounted in Mowiol for imaging. Excitation: 488 nm (**c**), 561 nm (**a**,**b**); detection: 650 – 725 nm; STED: 595 nm (**c**), 775 nm (**a**,**b**). Scale bar: 2 µm.

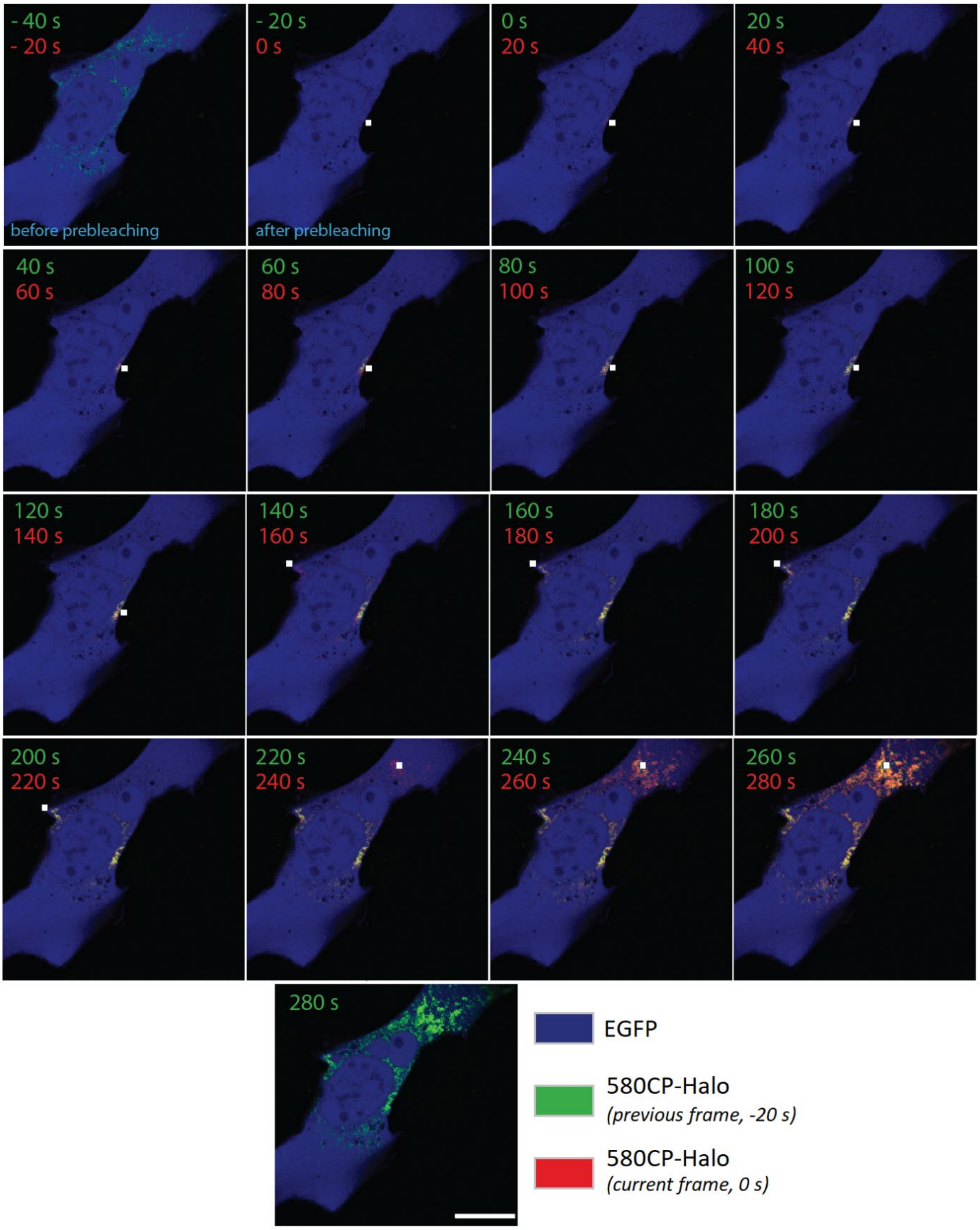

Figure S11. Confocal images of a living U2OS cell transfected with Tomm20-HT7-T2A-EGFP plasmid, recorded in DMEM buffer (+penicillin/streptomycin, +glutamine) with 0.5 μM HCage 580-Halo (**4ba-Halo**) before and after prebleaching step (with 595 nm STED light), and following repeated cycles of activation with a 405 nm laser (200 pulses with 10 μs dwell time at selected 2×2 μm^2 regions of interest outside the cells: □) every 20 s. Excitation: 561 nm, detection: 590-630 nm. Scale bar: 200 μm.

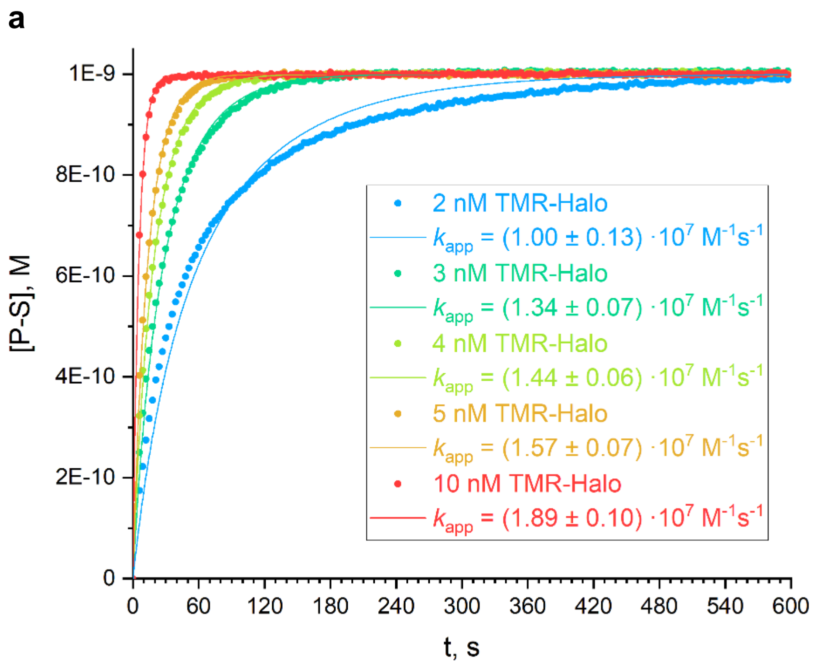

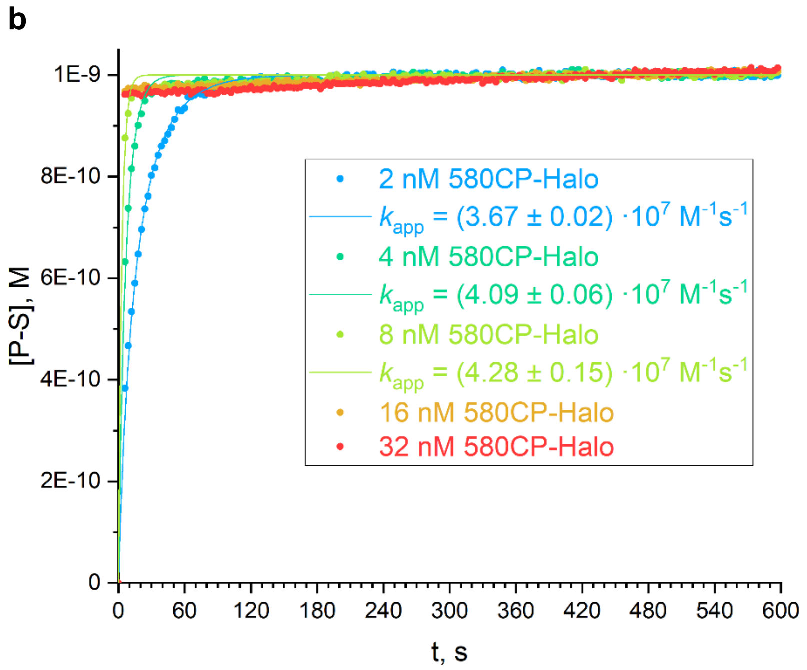

Figure S12. Reaction kinetics between HaloTag7 protein (1 nM) and TMR-Halo (control, **a**) or 580CP-Halo (uncaging product of HCage 580-Halo, **b**) fluorescent ligands monitored by fluorescence polarization in 0.1% CHAPS-PBS at pH 7.4. The averaged apparent second order rate constants are: for TMR-Halo, $(1.45 \pm 0.33) \cdot 10^{7}$ $M^{-1}s^{-1}$ (lit.: $(1.88 \pm 0.01) \cdot 10^{7}$ $M^{-1}s^{-1}$ [1]); for 580CP-Halo, $(4.01 \pm 0.31) \cdot 10^{7}$ $M^{-1}s^{-1}$.

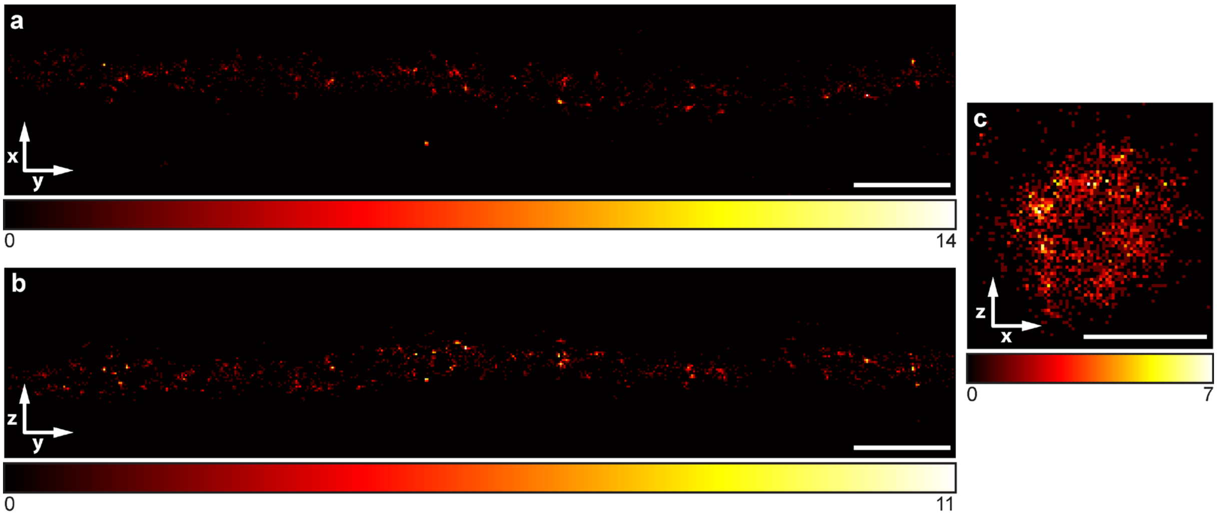

Figure S13. Side projections (**a,b**) and a projection along the estimated tubule path (**c**) of all MINFLUX localizations after filtering displayed as histogram. Pixel size: 4 nm (**a, b**), 1.5 nm (**c**). Scale bars: 200 nm (**a,b**), 50 nm (**c**).

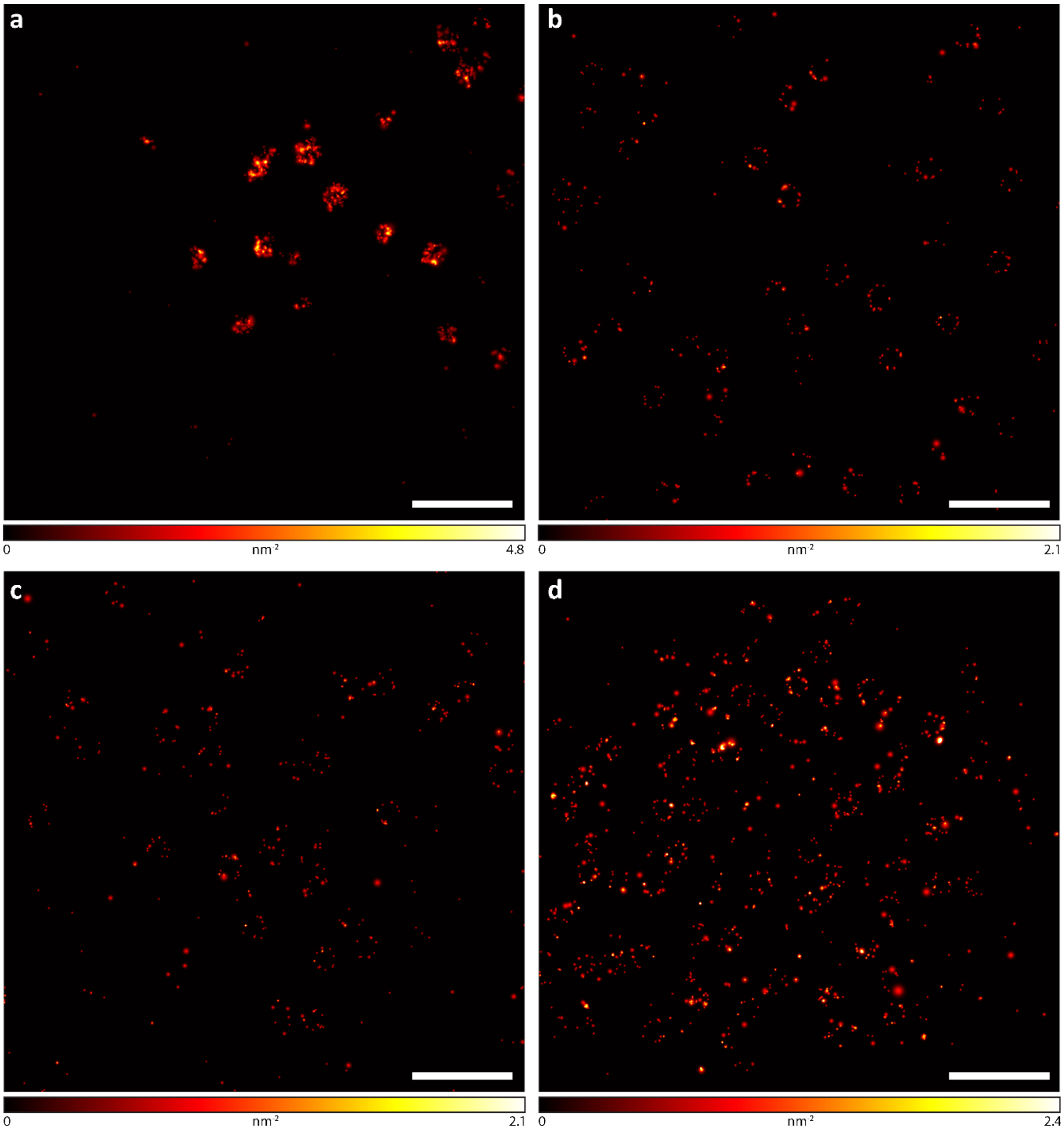

Figure S14. Full MINSTED images of fixed U2OS cells: (**a**) caveolin-1 labeled with primary and secondary antibody with HCage 620, (**b-c**) Nup96 endogeneously tagged with SNAP-tag (**b**) or HaloTag (**c**) and labeled with HCage 620 SNAP-tag (**4ca-BG**) or HaloTag ligand (**4ca-Halo**), respectively. (**d**) Full MINSTED images of fixed HeLa cells endogeneously expressing Nup107-mEGFP and labeled with single-domain anti-GFP nanobody and HCage 620-maleimide (**4ca-maleimide**). Scale bars: 500 nm.

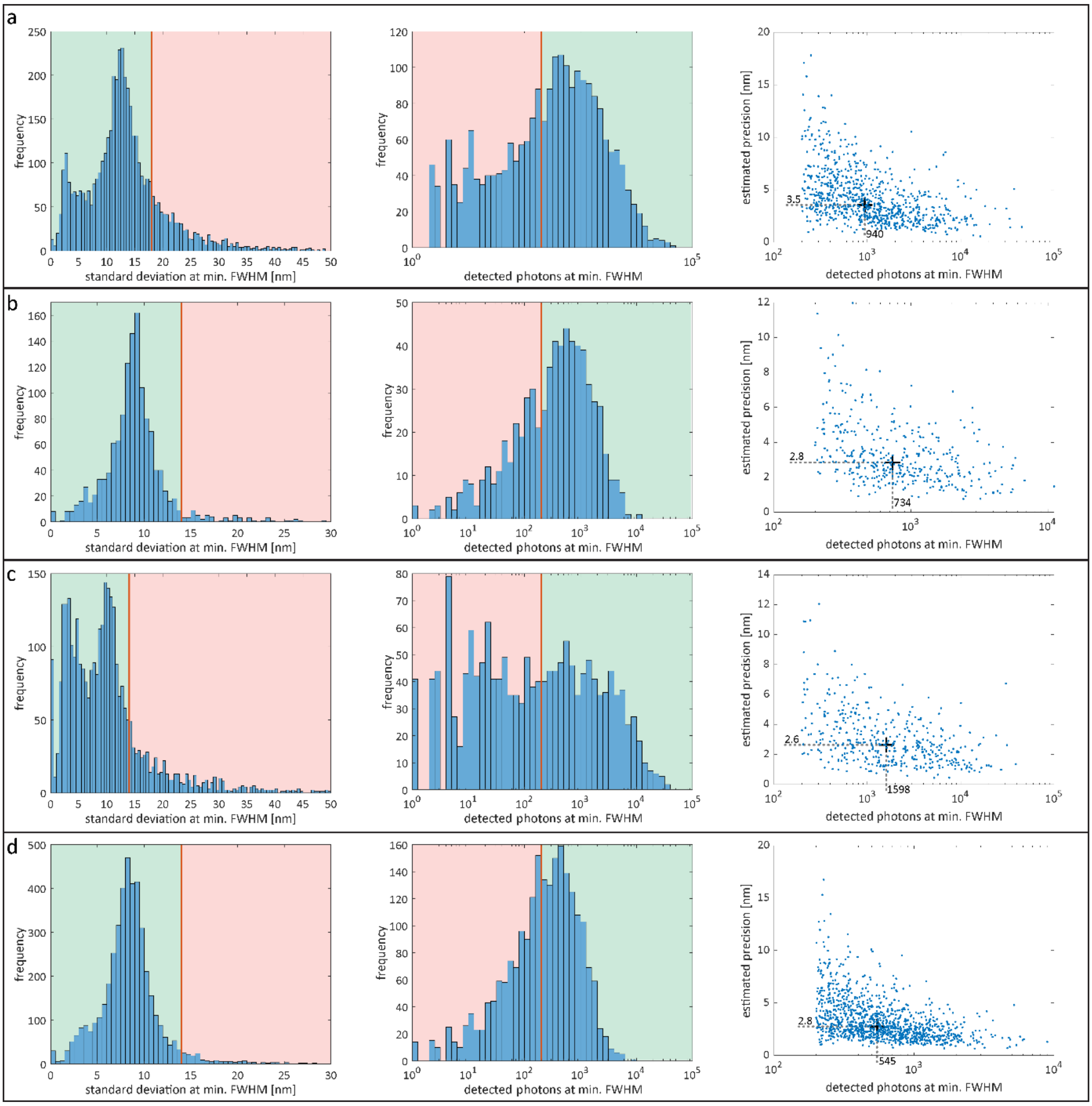

Figure S15. Distribution of the standard deviation (first column), distribution of the number of detected photons at minimal full width at minimal half maximum (FWHM; second column) and the estimated precision *vs.* detected photons at minimal FWHM of the MINSTED measurements of caveolin (**a**), NUP96-SNAP (**b**), NUP-Halo (**c**) and NUP107-mEGFP (**d**) labeled with HCage 620 (**4ca**), with the median estimated precision and number of photons indicated (third column). The localizations corresponding to the red areas of the histograms are rejected.

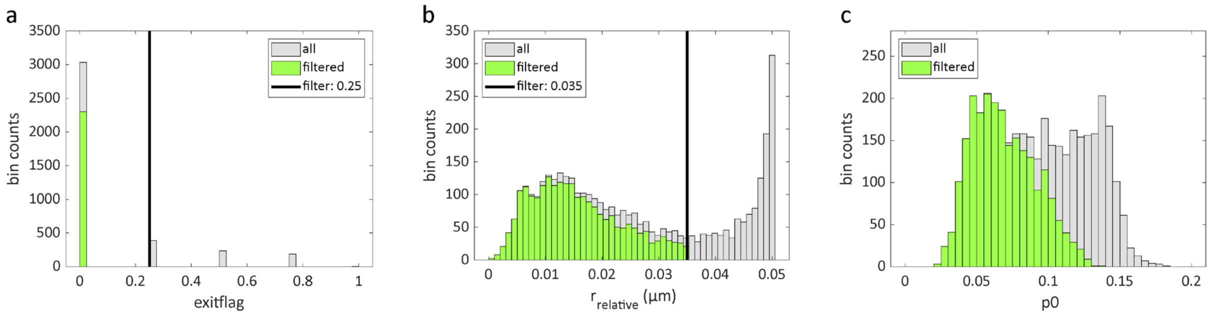

Figure S16. Histogram of the MINFLUX localization parameters used for filtering and quality control, before (grey) and after filtering (green) and their filter value (black line). (a) exitflag value larger than 0 indicates localizations at any of the grid edges during the grid-search based estimator, (b) distance between the final position estimate and the center of the excitation beam pattern in the last iteration $r_{relative}$, (c) relative photon count number in the central exposure position *p0*. Two populations are visible in $r_{relative}$ and *p0*, and the population of localizations assigned to background events was discarded by filtering.

Supplementary Methods

Manipulation of cells

Antibody coupling

To couple the dyes to antibody, 400 µL (~1 mg) of secondary antibody (goat anti-rabbit: Dianova 111-005-003, or donkey anti-mouse: Dianova 715-005-151) was mixed with 40 µL 1 M $NaHCO_3$ and 15 µL DMSO premixed with 100 µg of NHS-modified dye. After 1 h of stirring, the coupled antibody was purified from the unreacted dye using a PD-10 (GE Healthcare) size exclusion column with PBS as elution buffer.

Nanobody coupling

Nanobody against GFP (Nanotag, X2a and X2b anti-GFP) were coupled with HCage 620 maleimide according to the recommended protocol of the supplier. In brief, 10 mM neutralized tris(2-carboxyethyl)phosphine (Sigma Aldrich, 646547) was added to the nanobody. After 30 min incubation on ice, the buffer was exchanged to 10 mM potassium phosphate pH 6, 2 mM ethylenediaminetetraacetic acid and 300 mM sodium chloride using a PD-10 (GE Healthcare) size exclusion column. To the solution, a 1/10 of the volume of 1 M Tris/HCl pH 8 was added and mixed with a 5-fold molar excess of maleimide-modified dye dissolved in DMSO. After quick vortexing, the solution was incubated for 90 min on ice overlayed with argon and purified using a PD-10 size exclusion column with 10 mM potassium phosphate pH 6, 2 mM ethylenediaminetetraacetic acid and 300 mM sodium chloride as elution buffer.

Cell culture and sample preparation

The human osteosarcoma cell line U2OS (European Collection of Authenticated Cell Cultures, cat. 92022711, lot 17E015) was cultivated on coverslips in McCoy's medium (Thermo Fisher Scientific, 16600082) supplemented with 10% (v/v) fetal bovine serum (Bio&SELL, S0615) and penicillin-streptomycin (Sigma Aldrich, P0781).

The cells for imaging of microtubules with glutaraldehyde fixation were prepared according to a modified protocol from [2]. In brief, the cells were pre-extracted using 0.4% (v/v) glutaraldehyde and 0.25% (v/v) Triton X-100 in PBS pH 7.4 at 37 °C for

90 s. The samples were fixed using 3% (v/v) glutaraldehyde in PBS pH 7.4 at 37 °C for 15 min and quenched using sodium borohydride (1 mg/ml) in water for 7 min. After washing with PBS and blocking/permeabilization with 3% (w/v) BSA and 0.2% (v/v) Triton X-100 in PBS for 90 min, primary (Thermo Fisher Scientific, cat. MA1-80017) and secondary antibody were applied in 3% (w/v) BSA in PBS with intermediate washes using PBS. For MINFLUX imaging, the cells were stained with a ternary antibody conjugated with Alexa Fluor 488 (Molecular Probes, A11055) for 30 min and washed with PBS. For methanol fixation, cells were fixed using cold methanol (-20 °C) for 4 min, blocked with 2% (w/v) BSA in PBS for 10 min. Primary alpha-tubulin (Abcam, cat. ab18251) and secondary antibody were applied for 1 h in 2% BSA (w/v) in PBS with intermediate washing with PBS.

For imaging of caveolae, the cells were fixed with 8% (w/v) paraformaldehyde in PBS at 37 °C for 5 min and permeabilized using 0.5% (v/v) Triton X-100 in PBS for 5 min. After blocking with 2% (w/v) BSA in PBS, samples were labelled with primary antibody against caveolin-1 (Cell Signaling, cat. 3267) for 1 h, washed with PBS and incubated with secondary antibody conjugated with HCage 620 for 1 h. After a subsequent wash with PBS, the cells were stained with a ternary antibody conjugated with Alexa Fluor 488 (Molecular Probes, A11055) for 30 min and washed with PBS.

U2OS cells endogenously expressing vimentin tagged with HaloTag [3] were cultivated on chambered glass slides like wild-type U2OS cells. For live-cell imaging, the cells were washed with HEPES-buffered DMEM media (Thermo Fisher Scientific, 21063029) without phenol red and supplemented with 1% penicillin-streptomycin (Sigma Aldrich, P0781) and imaged in the same buffer with 2 µM HCage 620-Halo (**4ca-Halo**).

U2OS cells expressing nuclear pore protein NUP96 endogenously tagged with Halo- and SNAP-tag (U-2 OS-CRISPR-NUP96-Halo clone #252 (330448, CLS GmbH) [4] and U-2 OS-CRISPR-NUP96-SNAP clone #33 (330444, CLS GmbH) [4]) were cultured on coverslips in McCoy's medium (Thermo Fisher Scientific, 16600082) supplemented with 10% (v/v) fetal bovine serum (Bio&SELL, S0615), 1% (v/v) non-essential amino acid solution (Thermo Fisher Scientific, 11140050) and 1% (v/v) penicillin-streptomycin (Sigma Aldrich, P0781). HeLa cells expressing nuclear pore protein NUP107 fused with mEGFP (HK-2xZFN-mEGFP-NUP107 (330676, CLS GmbH) [5]) were cultured in DMEM media (Thermo Fisher Scientific, 31053044)

supplemented with 10% (v/v) fetal bovine serum (Bio&SELL, S0615), 1% (v/v) sodium pyruvate (Sigma Aldrich, S8636) and 1 % (v/v) GlutaMax (Thermo Fisher Scientific, 35050038) on coverslips. The cells were fixed with 2.4% (w/v) paraformaldehyde in PBS for 15 min, quenched with 100 mM ammonium chloride in PBS and permeabilized with 0.5% (v/v) Triton X-100 in PBS with washings with PBS between the steps.

For MINSTED measurements, the samples were incubated for 20 min with freshly diluted silica shelled silver nanoplates (nanoComposix, SPSH1050; 2.5 µg/mL in PBS). In case of MINFLUX measurements, gold nanorods (Nanopartz, A12-25-980-CTAB-DIH-1-25) were diluted with twice the volume PBS, sonicated for 10 min and incubated for 45 min on the sample. After washing with PBS, the cells were mounted with buffer (20 mM HEPES pH 7, 150 mM NaCl) using Twinsil (Picodent).

Neuron culture and sample preparation

Cultures of dissociated rat hippocampal primary neurons were prepared from postnatal P0–P2 Wistar rats of either sex and as described in [6]. Procedures performed in this study were in compliance with the Animal Welfare Act of the Federal Republic of Germany (Tierschutzgesetz der Bundesrepublik Deutschland, TierSchG) and the Animal Welfare Laboratory Animal Regulations (Tierschutzversuchsverordnung). According to the TierSchG and the Tierschutzversuchsverordnung no ethical approval from the ethics committee is required for the procedure of sacrificing rodents for subsequent extraction of tissues, as executed in this study. The procedure for sacrificing P0–P2 rats performed in this study was supervised by animal welfare officers of the Max Planck Institute for Medical Research (MPImF) and conducted and documented according to the guidelines of the TierSchG (permit number assigned by the MPImF: MPI/T-35/18).

Samples were fixed for 20 min in 4% paraformaldehyde in PBS, pH 7.4, and quenched for 10 min in quenching buffer (PBS, 100 mM glycine, 100 mM ammonium chloride). Cells were permeabilized for 5 min in 0.1% Triton X-100 and blocked with 1% BSA (Fisher Scientific, cat. BP1600-100) for 1 h. Afterwards, the samples were incubated with primary antibodies from mouse isotype IgG2 and rabbit in PBS for 1 h at rt. Samples were washed in PBS, pH 7.4 and incubated with secondary antibodies anti-mouse and anti-rabbit in PBS, pH 9 for 1 h at rt. The samples were then refixed for 10

min in 4% paraformaldehyde in PBS, pH 7.4, and quenched for 10 min in quenching buffer (PBS, 100 mM glycine, 100 mM ammonium chloride). Samples were rinsed with ddH_2O, washed with PBS, pH 9 and incubated with a primary antibody from guinea pig in PBS, pH 9 for 1 h at rt. In parallel, a primary antibody isotype IgG1 from mouse was pre-mixed with a nanobody against mouse IgG1 in PBS, pH 7.4 for 1 h at rt. Subsequently, the nanobody-primary-antibody pre-mixed cocktail was diluted to a final dilution of the primary antibody of 1:400. A single domain antibody against vGLUT-1 conjugated to Abberior STAR 635P (Synaptic Systems, cat. N1602-Ab635P-S, 1:200), phalloidin-Alexa Fluor 488 (Thermo Fisher, cat. A12379, 1:200) and the secondary antibody against guinea pig were added to the premix. Samples were washed and incubated with the previously prepared cocktail in PBS, pH 9 for 1h at rt.

Primary antibodies used were: Gephyrin (Synaptic Systems, cat. 147 008, 1:200 dilution), PSD95 IgG2a (Abcam, cat. ab2723, 1:200 dilution), Bassoon (Synaptic Systems cat. 141 004, 1:200 dilution) and beta-II-spectrin IgG1 (BD Bioscience, cat. 612563, 1:400 dilution). Secondary antibodies used in this study were: HCage 620 anti-rabbit (in-house coupled with goat anti-rabbit: Dianova 111-005-003), HCage 580 anti-mouse (in-house coupled with donkey anti-mouse: Dianova 715-005-151), a single domain antibody against mouse IgG1 (NanoTag, cat. N2002-Ab580-S, 1:200) and AlexaFluor 405 anti-guinea pig (Abcam, cat. ab175678, 1:200). After washing, samples were embedded in Mowiol supplemented with DABCO. For labeling the secondary antibodies with HCage dyes, see the “Antibody Coupling” section above.

Cell membrane permeability experiments

For data shown in Supplementary figures S8, S10 and S11, U2OS cells stably expressing vimentin tagged with HaloTag [3] (Vim-Halo U2OS) and wild-type U2OS cells were cultured in high glucose DMEM supplemented with GlutaMAX (ThermoFisher, cat. No. 31966-021) and 10% fetal bovine serum (FBS, ThermoFisher, cat. No. s0115-500mL) in a humidified 5% CO_2 incubator at 37 °C. The cells were split once every week or at confluence. Cells were seeded on 0.17 mm thick borosilicate coverslips (diameter 18 mm) placed in 12-well plates (TPP Techno Plastic Products AG, cat. No. 92012). Wildtype U-2 OS cells were transfected with 2 µg of Tomm20-HaloTag7-T2A-GFP plasmid via Lipofectamine™ 2000 Transfection Reagent (ThermoFisher, cat. No. 11668-027) according to manufacturer's recommendations.

After lipofection, the cells were grown in a humidified 5% CO_2 incubator at 37 °C for 2 d. Live imaging was performed with Tomm20-HaloTag-T2A-GFP transfected U2OS cells or with Vim-Halo U2OS cells after diluting the HCage 580-Halo (**4ba-Halo**) conjugate to a concentration of 500 nM in preheated 37 °C DMEM (phenol red free) supplemented with GlutaMAX (ThermoFisher, cat. No. 21063-029 500mL). In the case of transfected U2OS cells, the GFP volume labeling signal (excitation at 485 nm) was used to recognize Tomm20-HaloTag-T2A-GFP expressing cells. Any initial signal appearing after exciting at 561 nm was bleached using the STED 595 nm line. After that, small regions of interest were illuminated with a CW 405 nm excitation train of 200 repetitions. The pixel size was set to 200 nm and the pixel dwell time to 10 µs. The signal of the uncaged HCage 580-Halo was then tracked for 15 frames with an activation pulse train between each image.

An HCage 620-Halo ligand (**4ca-Halo**) was also used in fixed Vim-Halo U2OS cells. Cells were fixed in paraformaldehyde for 15 min, followed by a 10 min quenching step with quenching buffer (PBS, 100 mM glycine, 100 mM ammonium chloride) and permeabilization in 0.1% Triton X-100. After blocking with 1% BSA, the cells were incubated with 1 µM **4ca-Halo** ligand for 30 min and washed in PBS, pH 7,4. Counterstaining of vimentin filaments was performed using primary anti-vimentin (Abcam, cat. Ab92547) and secondary anti-rabbit Alexa Fluor 488 (ThermoFisher, cat. A11034) antibodies. Samples were embedded in Mowiol supplemented with DABCO and imaged on an Abberior expert line microscope (Abberior Instruments GmbH, Germany) with pulsed STED lines at 775 nm and 595 nm, excitation lasers at 640 nm, 561 nm, 485 nm, 405 nm and 355 nm, and spectral detection. Detection windows were set to 650–725 nm, 600–630 nm and 505–560 nm to detect HCage 620, HCage 590 and Alexa Fluor 488, respectively.

Imaging and data processing

Imaging

For data shown in Figure 2, Supplementary Figures S8, S10 and S11, samples were imaged on an Abberior expert line microscope (Abberior Instruments GmbH, Germany) with pulsed STED lines at 775 nm and 595 nm, excitation lasers at 640 nm, 561 nm, 485 nm, 405 nm (CW) and 355 nm (CW), and spectral detection. All pulsed lasers were operated at 40 MHz. Detection windows were set to 650–725 nm, 600–630 nm, 505–

560 nm, and 420–475 nm to detect STAR635P/HCage 620, STAR580/HCage 580, Alexa Fluor 488 and Alexa Fluor 405, respectively. Images were acquired with a 100x/1.4 NA magnification oil immersion lens. STAR635P and STAR580 were imaged semi-simultaneously during a first acquisition with STED at 775 nm, while Alexa Fluor 488 was imaged afterwards using STED at 595 nm. Following the STED imaging of Alexa Fluor 488, which served as a bleaching step for STAR635P and STAR580, Alexa Fluor 405 was imaged. Subsequently, HCage 620 and HCage 580 were activated at 405 nm and then imaged in a second acquisition step with STED at 775 nm. Confocal images of the Alexa Fluor 488 channel were performed in all the image sequences to monitor lateral drift and used for image registration. Axial drift was minimized by the Z-focus drift compensation unit of the microscope. Laser powers, line accumulations and dwell times used for imaging are noted in Table S1. Pixel size was set to 30 nm for all images, pinhole was set to 100 µm (1 AU).

Table S1. Imaging parameters used for activation of the HCage dyes and imaging with the respective fluorophores (Figure 2, Supplementary Figure S5). Laser powers are referred to values measured at the back focal aperture. Note that imaging parameters can vary when imaging other structures due to different protein abundances or signal intensities.

Fluorophore	Excitation (µW)	STED (mW)	Line repetitions	dwell tme (µs)
STAR635	24.5	113	12	15
STAR580	23	304	10	15
STAR GREEN	11.9	7.6	12	7
Alexa Fluor 405	170	not used	2	10
Activation 405 nm*	3850	not used	3	10
HCage 620 *(uncaged)*	65	190	8	10
HCage 580 *(uncaged)*	46	304	6	10

* 200 nm pixel size used for this step.

The data shown in Figure 3(a,b), Supplementary Figures S3, S4, S6, S7 and S9, and Supplementary Video S1 were acquired on an Abberior expert line STED microscope

(Abberior Instruments GmbH) equipped with 485 nm, 561 nm and 640 nm 40 MHz pulsed excitation lasers, a pulsed 775 nm STED 40 MHz laser, an UPlanSApo 100x/1.40 Oil objective and a wide-field 400 nm UV led (CoolLed). The fluorescence was detected using two detection windows between 605-625 nm and 650-720 nm. Pixel size and pinhole diameter were adapted for each imaging application.

For iterative 3D MINFLUX imaging, the microscope described in [7] equipped with a 642 nm laser for excitation and a 405 nm laser for activation was used. Regions of interest were selected based on a widefield EMCCD camera recording at 488 nm excitation. For 3D MINFLUX imaging a previously described iteration scheme was used [7], having a targeted coordinate pattern with a diameter (L) equal to 100 nm in the last iteration. To account for the higher sensitivity of photoactivatable dyes compared to cyanines with thiol induced blinking, the activation laser was attenuated using a neutral density filter and the excitation laser power was adjusted for each iteration accordingly.

MINSTED imaging was performed using the microscope described in [8]. In brief, the fluorophores were activated using a wide-field 355 nm laser and searched in raster-scanned overview images using a 635 nm laser and an APD detecting fluorescence between 640 nm and 720 nm. Active fluorophores were localized using a 775 nm STED laser. The minimal full width at half maximum of the effective point spread function and scan pattern diameter of 74 nm was used in case of caveolin, whereas 54 nm was used for nuclear pore samples.

Table S2. Total acquisition times for multiplexed STED, 3D MINFLUX and MINSTED images of the present work.

Figure	Imaging method	Sample area (μm^2) or volume (μm^3)	Time (s)
Figure 3 (c-e)	3D MINFLUX	0.4 μm^3	7080 (3600*)
Figure S5	STED (5x) + confocal (1x)	3600 μm^2	~ 3300
Figure S14 (a)	MINSTED	6.8 μm^2	930
Figure S14 (b)	MINSTED	6.8 μm^2	380
Figure S14 (c)	MINSTED	6.8 μm^2	800
Figure S14 (d)	MINSTED	6.8 μm^2	860

* 80% of localizations were accomplished within 60 min.

Image processing

The images were visualised and processed with Imspector (Abberior Instruments GmbH, Göttingen, Germany) and ImageJ 1.53c (https://imagej.nih.gov/ij/). Images of Alexa Fluor 405 were chromatically corrected with the ImageJ plugin DoM v.1.1.6 (https://github.com/ekatrukha/DoM_Utrecht), and image registration was carried out with the bUnwarpJ plugin version 2.6.12 [9] with the 488 channel as a reference image for all the measurements. Background subtraction was done with a rolling ball radius of 50 pixels and the brightness was adjusted uniformly to an eight of the maximum counts throughout the images for better visibility in the figures. Finally, the resulting images were smoothed with ImageJ, replacing each pixel with the average of its 3x3 neighbourhood. The images in Figure 3(a,b), Supplementary Figures S3, S4, S6, S7 and S9 and the Supplementary Video S3 are presented without processing.

MINFLUX localizations were processed as described in [7]. During the MINFLUX post-processing an optimized three-state Hidden-Markov model was used to segment the photon traces. To discard localizations due to background fluctuations two filters were applied: Localizations for which the grid-search based estimator fell to any grid edge and final localizations which are >35 nm apart from the estimated position in the previous iteration were excluded from further analysis as presented in Supplementary Figure S16. The photon trace of an emitter with more than 4000 photons was divided into multiple localizations with at least 2000 photons. The localization precision was calculated based on at least 4 such localizations assigned to the same emitter. The localizations were rendered with the estimated localization precision down to a minimal standard deviation of 3 nm. Localizations for which no localization precision could be calculated were only included in the non-rendered images as presented in Supplementary Figure S13.

MINSTED data were analysed as presented in [8]. The localizations acquired with MINSTED were filtered based on the standard deviation of the center positions after reaching the maximal STED power and the number of detected photons at this final step. The distributions of these parameters and the filter value are presented in Supplementary Figure S15.

General experimental information and synthesis

Thin layer chromatography

Analytical TLC (normal phase) was performed on Merck Millipore ready-to-use aluminum sheets coated with silica gel 60 (F_{254}) (Cat. No. 1.05554.0001). Analytical TLC on reversed phase (RP-C_{18}) was performed on Merck Millipore ready-to-use aluminum sheets coated with RP-18 60 (F_{254}s) (Cat. No. 1.05560.0001). Compounds were detected by exposing TLC plates to UV-light (254 or 366 nm) or by heating with vanillin stain (6 g vanillin and 1.5 mL conc. H_2SO_4 in 100 mL ethanol), 1 N NaOH or 1 N HCl as indicated.

Preparative flash column chromatography

Automated separations on normal phase were performed with an Isolera Spektra One system (Biotage AG, Sweden) using commercially available cartridges of suitable size (RediSep Rf series from Teledyne ISCO, Puriflash Silica HP 30µm series from Interchim) and solvent gradient indicated. Automated separations on reversed phase (C18, Amino) were performed on the cartridge flow path of a Reveleris Prep system (Büchi Labortechnik AG, Switzerland) using the type of cartridge and solvent gradient as indicated.

High-Performance Liquid Chromatography (HPLC) and Mass Spectrometry (MS)

Analytical liquid chromatography-mass spectrometry was performed on an LC-MS system (Shimadzu): 2x LC-20AD HPLC pumps with DGU-20A3R solvent degassing unit, SIL-20ACHT autosampler, CTO-20AC column oven, SPD-M30A diode array detector and CBM-20A communication bus module, integrated with CAMAG TLC-MS interface 2, FCV-20AH_2 diverter valve and LCMS-2020 spectrometer with electrospray ionization (ESI, 100 – 1500 m/z). Analytical column: ThermoScientific Hypersil Gold 50×2.1 mm 1.9µm, standard conditions: sample volume 1-2 µL, solvent flow rate 0.5 mL/min, column temperature 30 °C. General method: isocratic 95:5 A:B over 2 min, then gradient 95:5 to 0:100 A:B over 5 min, then isocratic 0:100 A:B over 2 min; solvent A – water + 0.1% (v/v) HCO_2H, solvent B – acetonitrile + 0.1% (v/v) HCO_2H.

High resolution mass spectra (HRMS) were obtained on a maXis II ETD (Bruker) with electrospray ionization (ESI) at the Mass Spectrometry Core facility of the Max-Planck Institute for Medical Research (Heidelberg, Germany).

Preparative high-performance liquid chromatography was performed on a Büchi Reveleris Prep system using the suitable preparative columns and conditions as indicated for individual preparations. Method scouting was performed on a HPLC system (Shimadzu): 2x LC-20AD HPLC pumps with DGU-20A3R solvent degassing unit, CTO-20AC column oven equipped with a manual injector with a 20 µL sample loop, SPD-M20A diode array detector, RF-20A fluorescence detector and CBM-20A communication bus module; or on a Dionex Ultimate 3000 UPLC system: LPG-3400SD pump, WPS-3000SL autosampler, TCC-3000SD column compartment with 2× 7-port 6-position valves and DAD-3000RS diode array detector. The test runs were performed on analytical columns with matching phases (HPLC: Interchim 250×4.6 mm 10 µm C18HQ, Interchim 250×4.6 mm 5 µm PhC4, solvent flow rate 1.2 mL/min; UPLC: Interchim C18HQ or PhC4 75×2.1 mm 2.2 µm, ThermoScientific Hypersil Gold 100×2.1 mm 1.9 µm, solvent flow rate 0.5 mL/min).

Optical spectroscopy

Absorption spectra were recorded with a Varian Cary 4000 UV-Vis double-beam spectrophotometer (Agilent Technologies, USA). The emission spectra were recorded with a Varian Cary Eclipse fluorescence spectrophotometer (Agilent). The absorption and emission spectra were recorded in quartz cells (optical path length 1 cm). Fluorescence quantum yields (absolute values) were obtained with a Quantaurus-QY absolute PL quantum yield spectrometer (model C11347-11, Hamamatsu) according to the manufacturer’s instructions. All measurements were performed in air-saturated solvents at ambient temperature. Fluorescence lifetimes of 600SiR dye were measured with a FluoTime 300 fluorescence lifetime spectrometer FluoTime 300 (PicoQuant) using a picosecond pulsed diode laser LDH-D-TA-560 (PicoQuant, SN: 01029652) and the manufacturer’s EasyTau 2 fitting/analysis software, in air-saturated solvents thermostated at 25 °C.

Series of absorption and fluorescence emission spectra were recorded in triplicate with a CLARIOstar Plus microplate reader (BMG LABTECH GmbH, Germany) in 96-well microplates (200 µL/well): non-binding polystyrene F-bottom, µClear (Greiner Bio-One GmbH, Ref. 655906) for aqueous solutions, polypropylene F-bottom (Greiner Bio-One

GmbH, Ref. 655201) for dioxane-water mixtures. The spectra were recorded at 25 °C in air-saturated solvents and are background corrected.

HaloTag7 labeling kinetics

Kinetic measurements by means of fluorescence polarization (FP) were performed on a CLARIOstar Plus microplate reader as described in [10]. More specifically, a polystyrene black flat-bottom non-binding 96 well plate (Greiner Bio-One, part Nr. 655900) was loaded with a series of concentrations (0 … 64 nM; 100 µL each) of HaloTag7 protein in 0.1% CHAPS – PBS pH 7.4. The injector was loaded with a 2 nM solution of the corresponding fluorescent ligand (TMR-Halo or 580CP-Halo) in 0.1% CHAPS – PBS pH 7.4. Mixing was performed in a well-by-well readout mode (fast kinetics) by injecting (at 300 µL/s) 100 µL of the ligand solution and double orbital shaking for 3 s. Fluorescence polarization readout was started 6 s from the injection point at 3 s intervals for the total time of 10 min. The optic settings were set as follows:

for TMR-Halo: excitation filter 540-20 nm, dichroic filter 566 nm (long pass), emission filter 590-20 nm, gain: 1600;

for 580CP-Halo: excitation filter 590-50 nm, dichroic filter 639 nm (long pass), emission filter 675-50 nm, gain: 1600.

Experiments were run in triplicates and the results were fitted for every protein concentration in Origin 2020b using the following equation:

$$[P-S] = C_0(P) \cdot \frac{1-e^{k_{app}(C_0(P)-C_0(S))t}}{1-\frac{C_0(P)}{C_0(S)} \cdot e^{k_{app}(C_0(P)-C_0(S))t}}$$

where C_0(P) and C_0(S) – initial concentrations of the protein and the substrate (HaloTag ligand) after mixing (in mol/L), t – time past mixing point (in s), k_{app} – apparent second-order reaction rate constrant (in $M^{-1}s^{-1}$, to be determined), and [*P*–*S*] – concentration of the bound substrate (in mol/L), determined from the FP reading at time t as follows:

$$[P-S](t) = \frac{FP(t) - FP_{min}}{FP_{max} - FP_{min}} \cdot C_0(S)$$

where FP_{min} is the value of FP as read out in the absence of protein (C_0(P) = 0) and FP_{max} is the value of FP determined in the presence of excess protein (≥32 equiv) after 10 min equilibration time.

NMR spectra

NMR spectra were recorded at 25 °C with a Bruker Ascend 400 spectrometer at 400.15 MHz (^{1}H), 376.52 MHz (^{19}F) and 100.62 MHz (^{13}C) and are reported in ppm. All ^{1}H spectra are referenced to tetramethylsilane as an internal standard (δ = 0.00 ppm). ^{13}C spectra are referenced to tetramethylsilane (δ = 0 ppm) using the signals of the solvent: $CDCl_3$ (77.16 ppm), CD_3OD (49.00 ppm), $\underline{C}D_3CN$ (1.32 ppm), acetone-d_6 (29.84 ppm) or DMSO-d_6 (39.52 ppm). Multiplicities of the signals are described as follows: s = singlet, d = doublet, t = triplet, q = quartet, m = multiplet or overlap of non-equivalent resonances; br = broad signal. Coupling constants $^{n}J_{X\text{-}Y}$ are given in Hz, where n is the number of bonds between the coupled nuclei X and Y ($J_{H\text{-}H}$ are always listed as J without indices).

Preparation of the starting materials

5-(Methoxycarbonyl)-2-nitrobenzyl 1H-imidazole-1-carboxylate (S1)

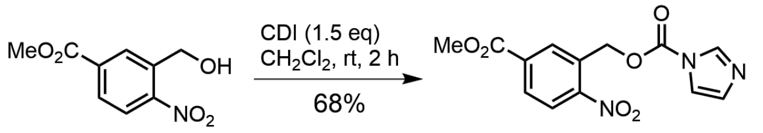

S1

1,1'-Carbonyldiimidazole (CDI; 972 mg, 6 mmol, 1.5 eq) was added portionwise to a stirred solution of methyl 3-(hydroxymethyl)-4-nitrobenzoate [11] (844 mg, 4 mmol) in dry CH_2Cl_2 (20 mL). After stirring for 2 h at rt, sat. aq. NH_4Cl (20 mL) was added and the reaction mixture was extracted with CH_2Cl_2 (3 × 20 mL). The combined extracts were dried over Na_2SO_4, filtered, evaporated, the residue was dissolved in EtOAc and the solution was passed through a plug of silica gel, washing with EtOAc. The filtrate was evaporated to viscous light yellow oil, which was freeze-dried from 1,4-dioxane to provide **S1** as yellowish solid (832 mg, 68%).

^{1}H NMR (400 MHz, $CDCl_3$): δ 8.31 (dd, J = 1.7, 0.8 Hz, 1H), 8.23 (dd, J = 8.5, 1.7 Hz, 1H), 8.20 (d, J = 8.4 Hz, 1H), 7.46 (t, J = 1.5 Hz, 1H), 7.11 (d, J = 1.5 Hz, 1H), 5.84 (s, 2H), 3.99 (s, 3H).
^{13}C NMR (101 MHz, $CDCl_3$): δ 164.7, 150.3, 148.3, 137.3, 135.1, 131.2, 131.1, 131.0, 130.3, 125.7, 117.3, 65.9, 53.2.
HRMS ($C_{13}H_{11}N_3O_6$): *m/z* (positive mode) = 306.0718 (found $[M+H]^+$), 306.0721 (calc.).

Methyl 3-(methylcarbamoyloxy)methyl-4-nitrobenzoate (2a)

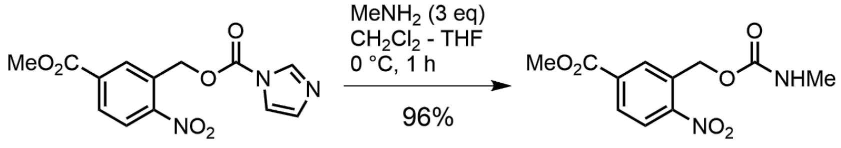

S1 **2a**

To a solution of **S1** (610 mg, 2 mmol) in dry CH_2Cl_2 (7 mL), colled in ice-water bath, methylamine (2 M in THF; 3 mL, 6 mmol, 3 eq) was added dropwise. The reaction mixture was stirred at 0…5 °C for 1 h and evaporated on Celite. The product was isolated by flash chromatography on Biotage Isolera system (40 g Teledyne ISCO RediSep Rf cartridge, gradient 20% to 100% EtOAc/hexane) to yield 512 mg (96%) of **2a** as white solid.

^{1}H NMR (400 MHz, $CDCl_3$): δ 8.26 (s, 1H), 8.10 (s, 2H), 5.51 (s, 2H), 4.91 (br.s, 1H), 3.97 (s, 3H), 2.84 (d, J = 4.9 Hz, 3H).
^{13}C NMR (101 MHz, $CDCl_3$): δ 165.2, 156.3, 150.0, 134.6, 133.6, 130.3, 129.8, 125.1, 62.9, 53.0, 27.8.

HRMS ($C_{11}H_{12}N_2O_6$): *m/z* (positive mode) = 269.0767 (found $[M+H]^+$), 269.0768 (calc.).

Methyl 3-(carbamoyloxy)methyl-4-nitrobenzoate (2b)

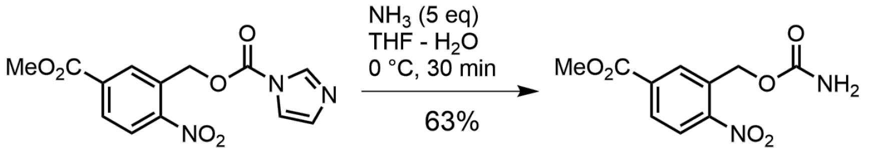

S1 **2b**

To a solution of **S1** (196 mg, 0.64 mmol) in THF (2.5 mL), colled in ice-water bath, aq. ammonia (25%, ~13 M in water; 0.25 mL, 3.21 mmol, 5 eq) was added dropwise. The reaction mixture was stirred at 0-5 °C for 30 min, diluted with methanol and evaporated to dryness on Celite. The product was isolated by flash chromatography on Biotage Isolera system (12 g Interchim SiHP 30 µm cartridge, gradient 20% to 100% EtOAc/hexane) to yield 103 mg (63%) of **2b** as white solid.

^{1}H NMR (400 MHz, DMSO-d$_6$): δ 8.21 (d, J = 8.5 Hz, 1H), 8.18 (d, J = 2.0 Hz, 1H), 8.10 (dd, J = 8.5, 2.0 Hz, 1H), 6.98 (br.s, 1H), 6.69 (br.s, 1H), 5.35 (s, 2H), 3.92 (s, 3H).
^{13}C NMR (101 MHz, DMSO-d$_6$): δ 164.7, 156.0, 149.7, 133.8, 133.7, 129.5, 129.3, 125.4, 61.3, 52.9.
HRMS ($C_{10}H_{10}N_2O_6$): *m/z* (positive mode) = 255.0611 (found $[M+H]^+$), 255.0612 (calc.).

General synthetic procedures for the preparation of caged fluorescent dyes

General procedure 1 (Buchwald-Hartwig amidation): compound 3aa

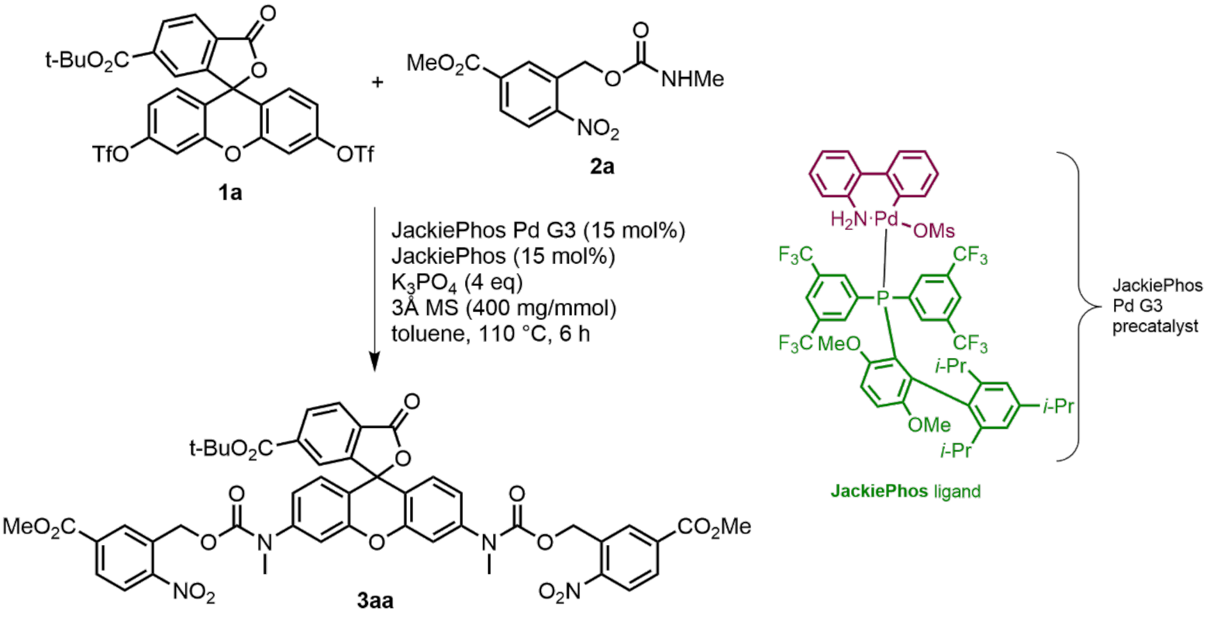

In a flame-dried 10 mL tube, loaded with anhydrous K_3PO_4 (119 mg, 0.56 mmol, 4 eq) and 3Å molecular sieves (56 mg), 6'-(*tert*-butoxycarbonyl)fluorescein ditriflate [12] (**1a**; 98 mg, 0.14 mmol), compound **2a** (90 mg, 0.34 mmol, 2.4 eq), JackiePhos Pd G3 precatalyst (24.5 mg, 0.021 mmol, 15 mol%) and JackiePhos ligand (16.7 mg, 0.021 mmol, 15 mol%) were loaded. The tube was sealed, anhydrous toluene (1.4 mL) was injected, the mixture was degassed on a Schlenk line and stirred at 110 °C for 6 h. It was then diluted with CH_2Cl_2, filtered through a plug of Celite (washing with CH_2Cl_2 and EtOAc-CH_2Cl_2), the filtrate was evaporated on Celite and the product was isolated by flash chromatography on Biotage Isolera system (25 g Interchim SiHP 30 µm cartridge, gradient 20% to 80% EtOAc/hexane) and freeze-dried from dioxane to yield 92 mg (70%) of **3aa** as white solid.

^{1}H NMR (400 MHz, $CDCl_3$): δ 8.25 (dd, J = 8.0, 1.3 Hz, 1H), 8.17 (br.s, 2H), 8.12 – 8.05 (m, 5H), 7.84 (s, 1H), 7.27 (d, J = 2.2 Hz, 2H), 7.05 (dd, J = 8.5, 2.2 Hz, 2H), 6.82 (d, J = 8.5 Hz, 2H), 5.68 – 5.52 (m, 4H), 3.90 (s, 6H), 3.39 (s, 6H), 1.54 (s, 9H).
^{13}C NMR (101 MHz, $CDCl_3$): δ 168.5, 165.0, 164.1, 154.4, 153.0, 151.5, 150.0, 145.1, 138.7, 134.6, 132.8, 131.3, 130.5, 130.0, 129.4, 128.8, 125.4, 125.23, 125.19, 121.5, 116.3, 114.1, 82.8, 82.2, 64.2, 53.0, 37.8, 28.1.
HRMS ($C_{47}H_{40}N_4O_{17}$): m/z (positive mode) = 933.2462 (found $[M+H]^+$), 933.2461 (calc.).

General procedure 2 (hydrolysis of methyl ester): compound 3aa-OH

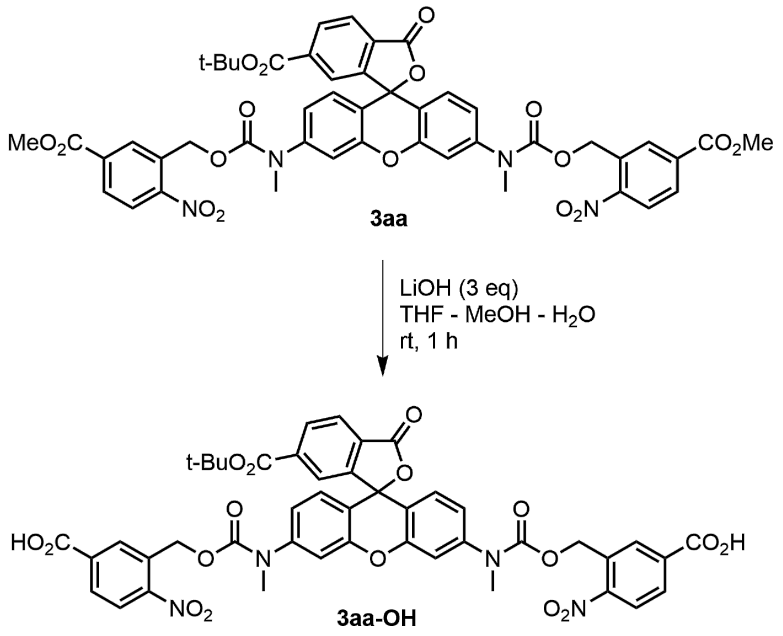

To a solution of compound **3aa** (40 mg, 42.9 µmol) in THF (400 µL) and methanol (200 µL), a solution of LiOH·H_2O (5.4 mg, 129 µmol, 3 eq) in water (85 µL) was added, and the resulting mixture was stirred vigorously at rt for 1 h. Acetic acid (200 µL) was then added, the reaction mixture was evaporated to dryness and dried in vacuo. The product was isolated by preparative HPLC (column: ThermoScientific 250×21.2 mm 5 µm Hypersil Gold C18; gradient 40/60 → 90/10 A:B, A = 0.1% v/v HCO_2H in acetonitrile, B = 0.1% v/v HCO_2H in water; detection at 220 nm), fractions containing the product were evaporated (bath temperature 30 °C), and the residue was freeze-dried from aq. dioxane to give **3aa-OH** as yellowish solid (33 mg, 85%).

^{1}H NMR (400 MHz, acetone-d_6): δ 8.31 (dd, J = 8.0, 1.3 Hz, 1H), 8.27 (br.s, 2H), 8.20 (d, J = 8.3 Hz, 2H), 8.16 (dd, J = 8.3, 1.6 Hz, 2H), 8.13 (dd, J = 8.0, 0.8 Hz, 1H), 7.96 (dd, J = 1.3, 0.8 Hz, 1H), 7.48 (d, J = 2.2 Hz, 2H), 7.23 (dd, J = 8.6, 2.2 Hz, 2H), 6.95 (d, J = 8.6 Hz, 2H), 5.67 – 5.53 (m, 4H), 3.42 (s, 6H), 1.51 (s, 9H).
^{13}C NMR (101 MHz, acetone-d_6): δ 168.5, 165.8, 164.8, 154.9, 153.7, 152.2, 151.0, 146.4, 139.4, 135.8, 133.7, 132.0, 131.1, 130.8, 130.5, 129.4, 126.00, 125.98, 125.8, 122.2, 117.0, 114.6, 83.0, 82.7, 64.6, 37.7, 28.1.
HRMS ($C_{45}H_{36}N_4O_{17}$): *m/z* (positive mode) = 905.2150 (found $[M+H]^+$), 905.2148 (calc.).

<u>General procedure 3</u> (amide coupling): compound 4a

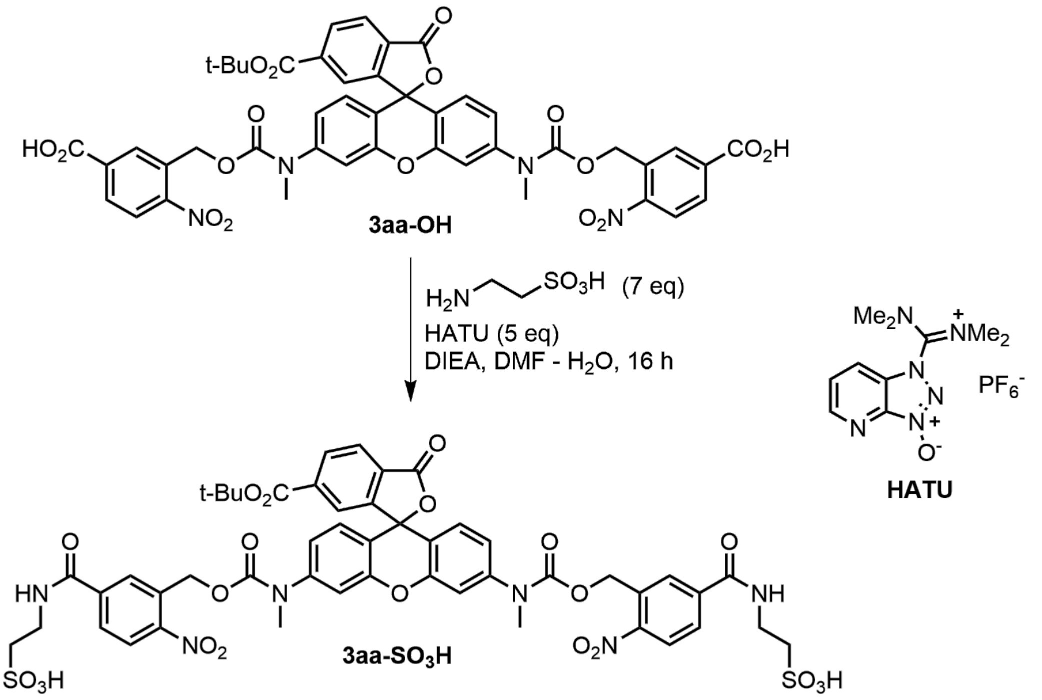

To a solution of compound **3aa-OH** (28 mg, 30.9 µmol) in DMF (500 µL) and water (100 µL), taurine (27 mg, 216 µmol, 7 eq) and *N*,*N*-ethyldiisopropylamine (DIEA; 300 µL) were added followed by HATU (59 mg, 155 µmol, 5 eq; dissolved in 200 µL DMF). The resulting mixture was stirred vigorously at rt overnight (16 h). It was then evaporated to dryness and dried in vacuo. The product was isolated by preparative HPLC (column: Interchim 250×21.2 mm 5 µm Uptisphere Strategy PhC4; gradient 20/80 → 70/30 A:B, A = 0.1% v/v HCO_2H in acetonitrile, B = 0.1% v/v HCO_2H in water; detection at 220 nm), fractions containing the product were evaporated (bath temperature 30 °C), and the residue was freeze-dried from aq. dioxane to give **3aa-SO$_3$H** as light orange solid (29 mg), which was used directly in the next step.

HRMS ($C_{49}H_{46}N_6O_{21}S_2$): *m/z* (positive mode) = 1119.2229 (found $[M+H]^+$), 1119.2230 (calc.).

General procedure 4 (hydrolysis of *tert*-butyl ester): caged dye 4aa (HCage 520)

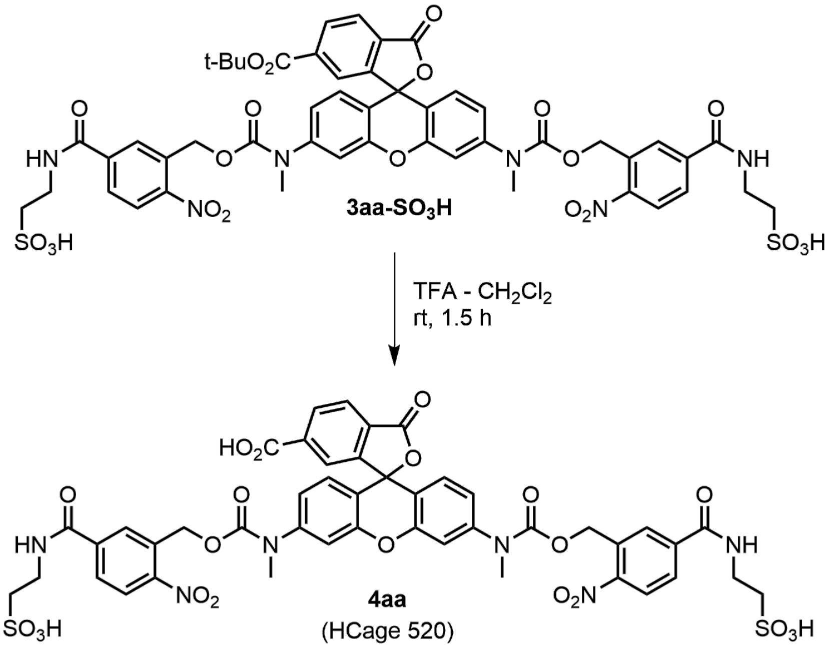

A solution of compound **4a** (28 mg, 25.0 µmol) in CH_2Cl_2 (600 µL) and TFA (300 µL) was stirred at rt for 1.5 h. The resulting mixture was diluted with CH_2Cl_2 and toluene, evaporated to dryness and dried in vacuo. The product was isolated by preparative HPLC (column: Interchim 250×21.2 mm 5 µm Uptisphere Strategy PhC4; gradient 20/80 → 60/40 A:B, A = 0.1% v/v HCO_2H in acetonitrile, B = 0.1% v/v HCO_2H in water; detection at 220 nm), fractions containing the product were evaporated (bath temperature 30 °C), and the residue was freeze-dried from aq. dioxane to give **5a** as light orange solid (15 mg, 46% over 2 steps).

^{1}H NMR (400 MHz, DMSO-d$_6$): δ 8.81 (t, J = 5.3 Hz, 2H), 8.27 (dd, J = 7.9, 1.3 Hz, 1H), 8.21 – 8.14 (m, 3H), 8.03 (br.s, 2H), 7.92 (dd, J = 8.5, 1.9 Hz, 2H), 7.87 (t, J = 1.0 Hz, 1H), 7.48 (d, J = 2.2 Hz, 2H), 7.19 (dd, J = 8.6, 2.2 Hz, 2H), 6.88 (d, J = 8.6 Hz, 2H), 5.54 – 5.44 (m, 4H), 3.51 (q, J = 6.8 Hz, 4H), 3.34 (s, 6H), 2.78 – 2.67 (t, J = 6.8 Hz, 4H).
^{13}C NMR (101 MHz, DMSO-d$_6$): δ 167.7, 166.0, 163.8, 153.9, 152.4, 150.7, 148.6, 145.0, 138.9, 137.6, 132.1, 131.3, 129.0, 128.4, 128.2, 127.4, 125.6, 125.2, 124.8, 121.5, 115.4, 113.3, 81.7, 63.8, 50.0, 37.1, 36.3.
HRMS ($C_{45}H_{38}N_6O_{21}S_2$): *m/z* (positive mode) = 1063.1604 (found $[M+H]^+$), 1063.1604 (calc.).

Compound 3ba

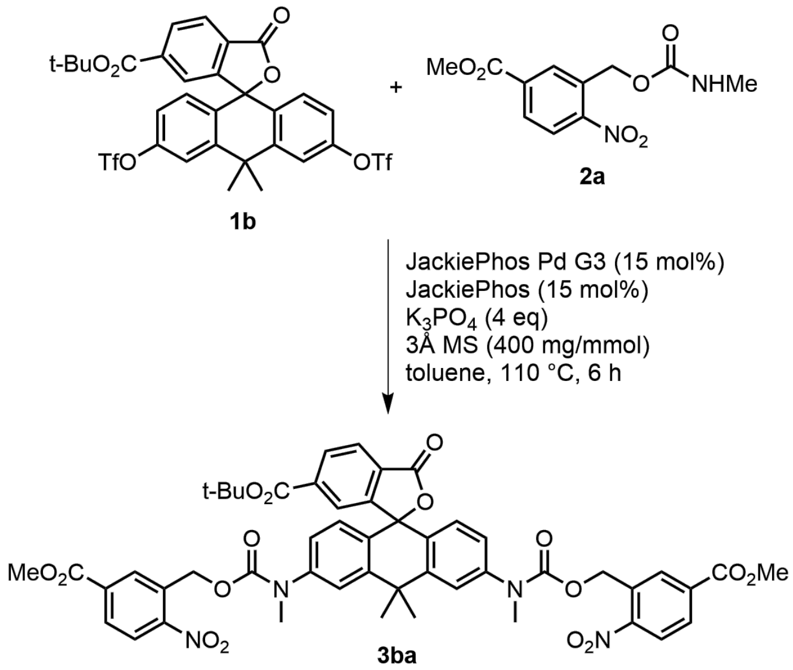

Prepared according to the general procedure 1 from 6'-(*tert*-butoxycarbonyl)carbofluorescein ditriflate [13] (**1b**; 108 mg, 0.14 mmol), compound **2a** (90 mg, 0.34 mmol, 2.4 eq), anhydrous K_3PO_4 (119 mg, 0.56 mmol, 4 eq) and 3Å molecular sieves (56 mg), JackiePhos Pd G3 precatalyst (24.5 mg, 0.021 mmol, 15 mol%) and JackiePhos ligand (16.7 mg, 0.021 mmol, 15 mol%). Yield 89 mg (66%) of **3ba** as white solid.

^{1}H NMR (400 MHz, $CDCl_3$): δ 8.20 (dd, J = 8.0, 1.3 Hz, 1H), 8.17 (br.s, 2H), 8.10 – 8.05 (m, 5H), 7.68 (br.s, 1H), 7.60 (br.s, 2H), 7.09 (dd, J = 8.5, 2.2 Hz, 2H), 6.78 (d, J = 8.5 Hz, 2H), 5.62 – 5.50 (m, 4H), 3.92 (s, 6H), 3.38 (s, 6H), 1.83 (s, 3H), 1.73 (s, 3H), 1.53 (s, 9H).
^{13}C NMR (101 MHz, $CDCl_3$): δ 169.6, 165.0, 164.3, 154.9, 154.7, 150.1, 146.0, 143.9, 138.3, 134.6, 133.0, 130.7, 130.5, 129.9, 129.2, 128.8, 125.3, 125.2, 125.1, 124.2, 85.7, 82.7, 64.1, 53.0, 38.5, 38.0, 34.8, 33.3, 28.1.
HRMS ($C_{50}H_{46}N_4O_{16}$): *m/z* (positive mode) = 959.2976 (found $[M+H]^+$), 959.2982 (calc.).

Compound 3ba-OH

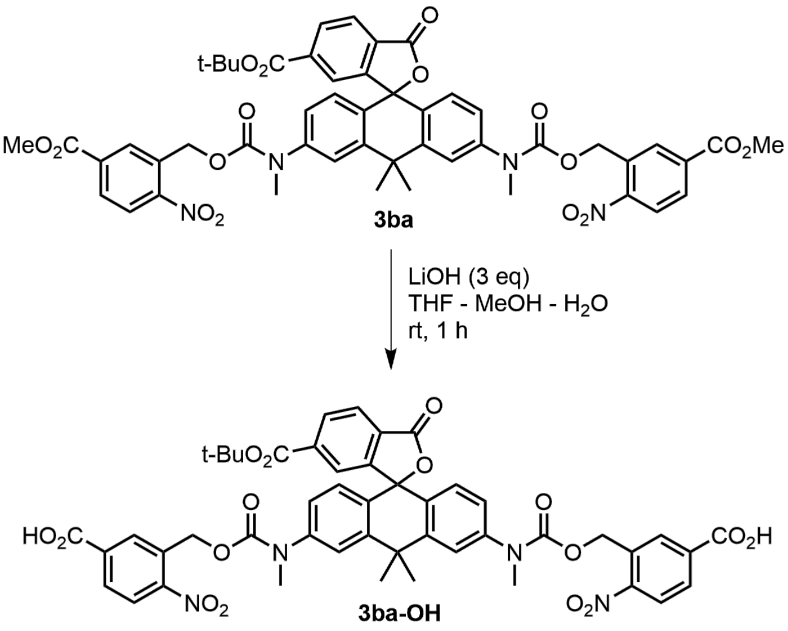

Prepared according to the general procedure 2 from compound **3ba** (40 mg, 41.7 µmol) in THF (400 µL) and methanol (200 µL) and a solution of LiOH·H_2O (5.3 mg, 125 µmol, 3 eq) in water (85 µL). Yield 33 mg (85%) of **3ba-OH** as light pink solid.

^{1}H NMR (400 MHz, acetone-d_6): δ 8.25 (dd, J = 8.0, 1.3 Hz, 1H), 8.25 (br.s, 2H), 8.21 – 8.15 (m, 4H), 8.12 (dd, J = 8.0, 0.8 Hz, 1H), 7.87 (d, J = 2.2 Hz, 2H), 7.67 (dd, J = 1.3, 0.8 Hz, 1H), 7.26 (dd, J = 8.6, 2.2 Hz, 2H), 6.87 (d, J = 8.6 Hz, 2H), 5.56 (s, 4H), 3.39 (s, 6H), 1.87 (s, 3H), 1.76 (s, 3H), 1.50 (s, 9H).

^{13}C NMR (101 MHz, acetone-d_6): δ 169.6, 165.9, 164.7, 156.1, 155.1, 151.2, 146.6, 145.1, 139.1, 135.8, 133.8, 131.4, 131.2, 130.8, 130.0, 129.3, 129.1, 126.2, 126.0, 125.3, 125.2, 125.1, 85.9, 83.0, 64.4, 39.1, 37.9, 34.6, 33.8, 28.1.

HRMS ($C_{48}H_{42}N_4O_{16}$): *m/z* (positive mode) = 931.2661 (found $[M+H]^+$), 931.2669 (calc.).

Compound 3ba-SO_3H

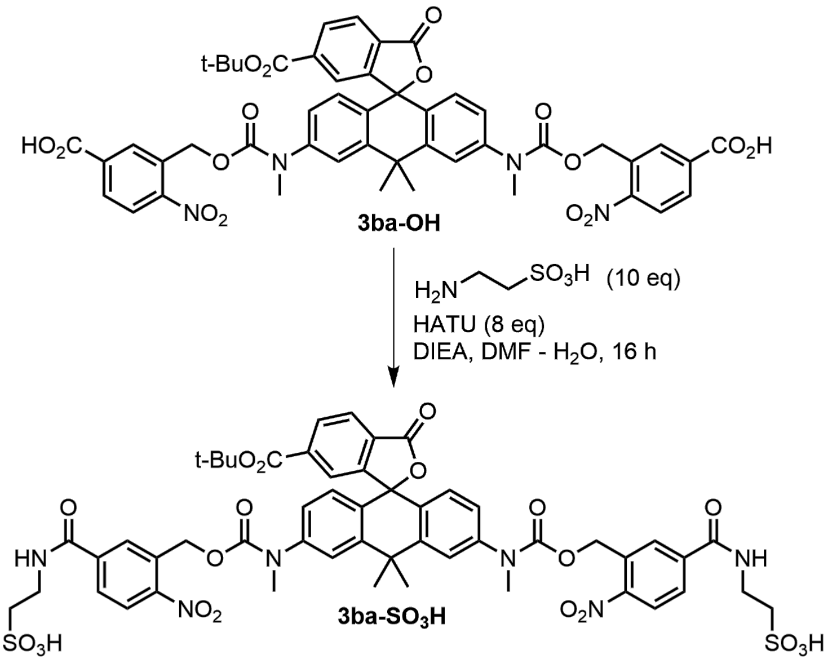

Prepared according to the general procedure 3 from a solution of compound **3ba-OH** (30 mg, 32.2 µmol) in DMF (500 µL) and water (100 µL), taurine (40 mg, 322 µmol, 10 eq) and *N*,*N*-ethyldiisopropylamine (DIEA; 350 µL) and HATU (98 mg, 258 µmol, 8 eq; dissolved in 300 µL DMF). Yield 24 mg (65%) of **3ba-SO_3H** as light pink solid.

^1H NMR (400 MHz, CD_3OD): δ 8.23 (dd, J = 8.0, 1.3 Hz, 1H), 8.14 – 8.07 (m, 3H), 8.04 (br.s, 2H), 7.92 (br.d, J = 8.5 Hz, 2H), 7.76 (br.s, 2H), 7.60 (s, 1H), 7.18 (dd, J = 8.6, 2.1 Hz, 2H), 6.81 (d, J = 8.6 Hz, 2H), 5.52 (s, 4H), 3.79 (t, J = 6.7 Hz, 4H), 3.70 (hept, J = 6.6 Hz, DIEA-H^+), 3.37 (s, 6H), 3.20 (q, J = 7.4 Hz, DIEA-H^+), 3.11 (t, J = 6.7 Hz, 4H), 1.80 (s, 3H), 1.70 (s, 3H), 1.49 (s, 9H), 1.37 – 1.32 (m, DIEA-H^+).
^{13}C NMR (101 MHz, CD_3OD): δ 171.1, 167.4, 165.3, 156.6, 156.4, 150.6, 147.2, 145.3, 140.2, 139.6, 133.9, 131.8, 130.1, 129.9, 129.7, 129.4, 128.7, 126.4, 126.2, 125.8, 125.5, 86.9, 83.6, 65.4, 55.8, 51.1, 43.8, 39.5, 38.2, 37.4, 34.8, 33.8, 28.2, 18.7, 17.3, 13.2 (including the signals of DIEA-H^+ counterion).
HRMS ($C_{52}H_{52}N_6O_{20}S_2$): *m/z* (positive mode) = 1145.2753 (found $[M+H]^+$), 1145.2751 (calc.).

Caged dye 4ba (HCage 580)

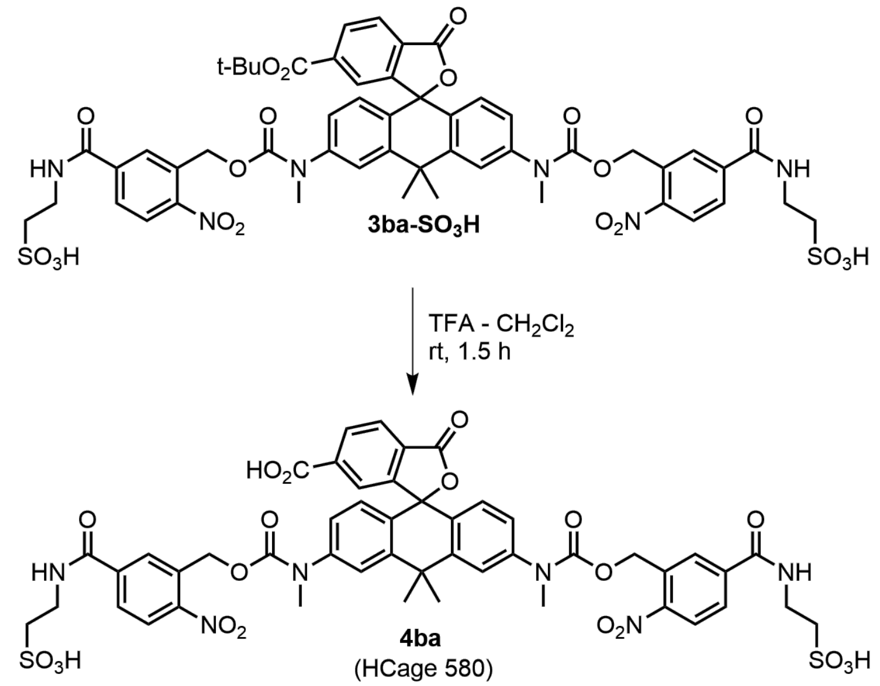

Prepared according to the general procedure 4 from compound **3ba-SO₃H** (24 mg, 21.0 µmol) in CH_2Cl_2 (600 µL) and TFA (300 µL). Yield 20 mg (88%) of **4ba** as light pink solid.

^{1}H NMR (400 MHz, DMSO-d$_6$): δ 8.84 (t, J = 5.4 Hz, 2H), 8.24 – 8.13 (m, 4H), 8.06 (s, 2H), 7.94 (d, J = 8.5 Hz, 2H), 7.81 (d, J = 2.2 Hz, 2H), 7.54 (s, 1H), 7.22 (dd, J = 8.6, 2.1 Hz, 2H), 6.74 (d, J = 8.6 Hz, 2H), 5.47 (s, 4H), 3.66 – 3.57 (m, DIEA-H$^+$), 3.54 (t, J = 7.1 Hz, 4H), 3.33 (s, 6H), 3.18 – 3.09 (m, DIEA-H$^+$), 2.76 (t, J = 7.1 Hz, 4H), 1.74 (s, 3H), 1.65 (s, 3H), 1.28 – 1.22 (m, DIEA-H$^+$).

^{13}C NMR (101 MHz, DMSO-d$_6$): δ 168.7, 166.0, 163.8, 154.7, 154.0, 148.8, 145.4, 143.7, 138.9, 137.5, 132.1, 130.8, 128.5, 128.4, 127.9, 127.5, 127.4, 125.8, 125.1, 124.4, 123.9, 84.9, 63.7, 53.6, 50.0, 41.9, 37.9, 37.4, 36.4, 33.7, 33.2, 18.1, 16.7, 12.5 (including the signals of DIEA-H$^+$ counterion).

HRMS ($C_{48}H_{44}N_6O_{20}S_2$): *m/z* (positive mode) = 1089.2129 (found [M+H]$^+$), 1089.2125 (calc.).

Compound 3ca

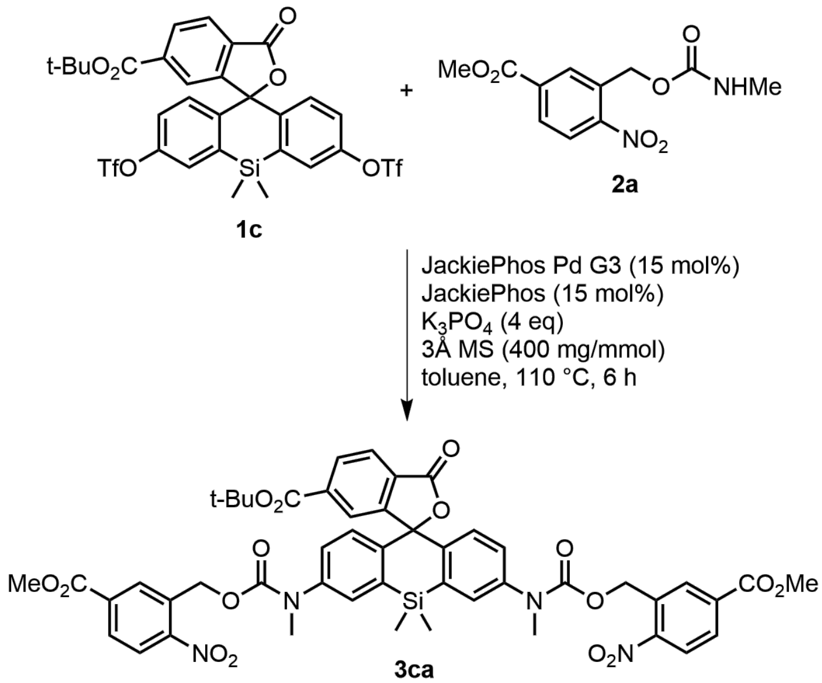

Prepared according to the general procedure 1 from 6'-(*tert*-butoxycarbonyl)silafluorescein ditriflate [14] (**1c**; 100 mg, 0.135 mmol), compound **2a** (87 mg, 0.325 mmol, 2.4 eq), anhydrous K_3PO_4 (115 mg, 0.54 mmol, 4 eq) and 3Å molecular sieves (55 mg), JackiePhos Pd G3 precatalyst (24 mg, 0.02 mmol, 15 mol%) and JackiePhos ligand (16 mg, 0.02 mmol, 15 mol%). Yield 68 mg (52%) of **3ca** as white solid.

^{1}H NMR (400 MHz, $CDCl_3$): δ 8.20 – 8.05 (m, 7H), 8.01 (dd, J = 8.1, 0.7 Hz, 1H), 7.91 (br.s, 1H), 7.62 (d, J = 2.4 Hz, 2H), 7.23 (dd, J = 8.6, 2.4 Hz, 2H), 7.16 (d, J = 8.6 Hz, 2H), 5.54 (s, 4H), 3.91 (s, 6H), 3.36 (s, 6H), 1.55 (s, 9H), 0.72 (s, 3H), 0.59 (s, 3H).
^{13}C NMR (101 MHz, $CDCl_3$): δ 169.8, 165.0, 164.1, 154.6, 154.2, 150.1, 142.7, 141.8, 137.7, 136.2, 134.6, 132.9, 131.0, 130.6, 129.9, 128.1, 127.4, 127.2, 126.2, 125.2, 125.1, 89.7, 82.7, 64.1, 53.0, 37.9, 28.2, -0.1, -0.6.
HRMS ($C_{49}H_{46}N_4O_{16}Si$): *m/z* (positive mode) = 975.2741 (found $[M+H]^+$), 975.2751 (calc.).

Compound 3ca-OH

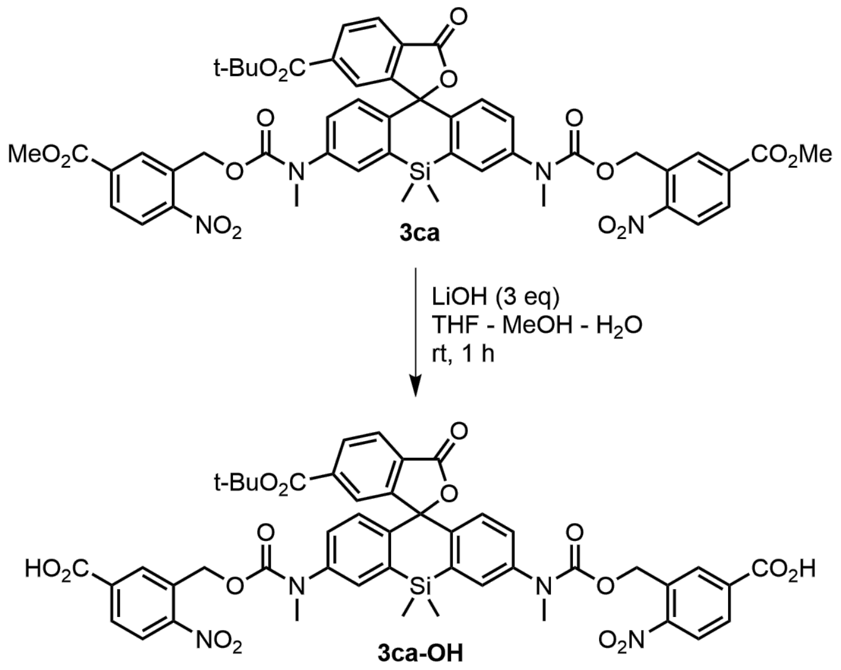

Prepared according to the general procedure 2 from a solution of compound **3ca** (20 mg, 20.5 µmol) in THF (200 µL) and methanol (100 µL) and a solution of LiOH·H_2O (2.6 mg, 61.5 µmol, 3 eq) in water (40 µL). Yield 14.5 mg (75%) of **3ca-OH** as white solid.

^{1}H NMR (400 MHz, acetone-d_6): δ 8.25 (br.s, 2H), 8.21 – 8.15 (m, 5H), 8.07 (dd, J = 8.0, 0.7 Hz, 1H), 7.90 (dd, J = 1.3, 0.7 Hz, 2H), 7.88 (d, J = 2.4 Hz, 2H), 7.41 (dd, J = 8.7, 2.4 Hz, 2H), 7.21 (d, J = 8.7 Hz, 2H), 5.54 (s, 4H), 3.36 (s, 6H), 1.52 (s, 9H), 0.74 (s, 3H), 0.60 (s, 3H).
^{13}C NMR (101 MHz, acetone-d_6): δ 169.9, 165.8, 164.6, 155.7, 155.1, 151.2, 143.9, 142.3, 138.6, 136.4, 135.8, 133.7, 131.8, 131.4, 131.3, 130.9, 128.5, 128.2, 127.8, 127.0, 126.0, 125.2, 89.9, 83.1, 64.4, 37.9, 28.1, -0.3, -0.4.
HRMS ($C_{47}H_{42}N_4O_{16}Si$): *m/z* (positive mode) = 947.2429 (found $[M+H]^+$), 947.2438 (calc.).

Compound 3ca-SO_3H

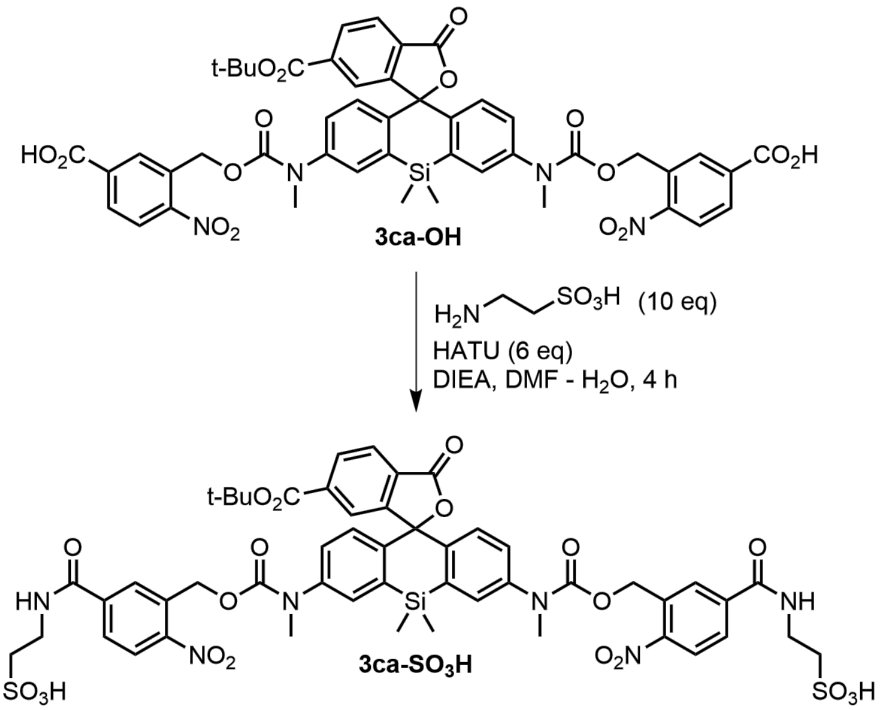

Prepared according to the general procedure 3 from compound **3ca-OH** (18.5 mg, 19.5 µmol) in DMF (300 µL) and water (100 µL), taurine (25 mg, 200 µmol, 10 eq), *N*,*N*-ethyldiisopropylamine (DIEA; 100 µL) and HATU (45 mg, 117 µmol, 6 eq; dissolved in 150 µL DMF). The product was isolated by preparative HPLC (column: Interchim 250×21.2 mm 5 µm Uptisphere Strategy PhC4; gradient 20/80 → 70/30 A:B, A = acetonitrile, B = 50 mM ammonium formate in water, pH = 3.5-4.0; detection at 220 nm), Yield 23.5 mg of **3ca-SO_3H** as pale violet solid (remainder ammonium formate).

^1H NMR (400 MHz, CD_3OD): δ 8.43 (s, 1H), 8.14 (dd, J = 8.0, 1.3 Hz, 1H), 8.12 – 7.99 (m, 5H), 7.92 (dd, J = 8.4, 1.9 Hz, 2H), 7.77 (s, 3H), 7.32 (dd, J = 8.7, 2.4 Hz, 2H), 7.17 (d, J = 8.7 Hz, 2H), 5.49 (s, 4H), 3.79 (t, J = 6.7 Hz, 4H), 3.34 (s, 6H), 3.10 (t, J = 6.7 Hz, 4H), 1.50 (s, 9H), 0.70 (s, 3H), 0.56 (s, 3H).
^{13}C NMR (101 MHz, CD_3OD): δ 171.4, 167.3, 165.2, 156.7, 156.4, 150.6, 144.1, 142.9, 140.2, 139.1, 136.7, 133.8, 132.5, 131.5, 129.8, 128.8, 128.4, 128.1, 127.2, 126.2, 125.3, 90.6, 83.7, 65.4, 51.1, 38.2, 37.4, 28.3, -0.1, -0.4.
HRMS ($C_{51}H_{52}N_6O_{20}S_2Si$): *m/z* (positive mode) = 1161.2518 (found $[M+H]^+$), 1161.2520 (calc.).

Caged dye 4ca (HCage 620)

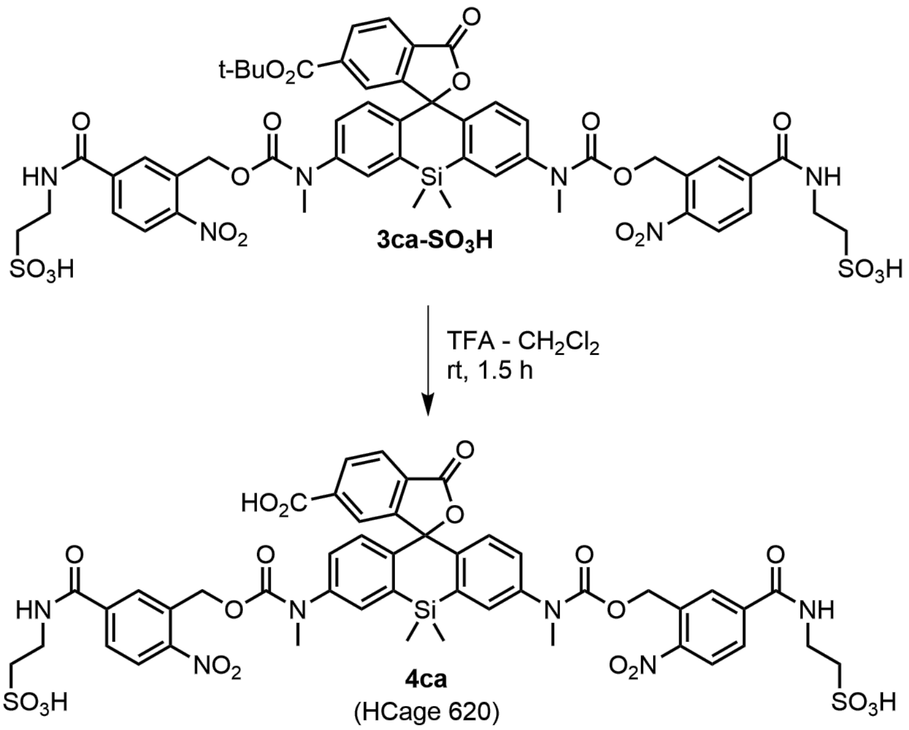

Prepared according to the general procedure 4 from compound **3ca-SO₃H** (23.5 mg, 19.5 μmol) in CH_2Cl_2 (500 μL) and TFA (250 μL). The product was isolated by preparative HPLC (column: Interchim 250×21.2 mm 5 μm Uptisphere Strategy PhC4; gradient 20/80 → 100/0 A:B, A = 0.1% v/v HCO_2H in acetonitrile, B = 0.1% v/v HCO_2H in water; detection at 220 nm) Yield 17.5 mg (81% over 2 steps) of **4ca** as bluish solid.

^{1}H NMR (400 MHz, DMSO-d$_6$): δ 8.84 (t, J = 5.3 Hz, 2H), 8.20 – 8.11 (m, 4H), 8.05 (br.s, 2H), 7.95 (dd, J = 8.4, 1.9 Hz, 2H), 7.86 – 7.79 (m, 3H), 7.37 (dd, J = 8.6, 2.5 Hz, 2H), 7.06 (d, J = 8.6 Hz, 2H), 5.46 (s, 4H), 3.62 – 3.52 (m, 6H), 2.82 – 2.71 (m, 4H), 0.64 (s, 3H), 0.52 (s, 3H).
^{13}C NMR (101 MHz, DMSO-d$_6$): δ 168.8, 165.9, 163.8, 154.0, 153.9, 148.7, 142.6, 140.6, 138.9, 136.9, 135.4, 132.0, 130.8, 130.7, 128.5, 127.5, 127.3, 127.2, 126.8, 126.6, 125.2, 124.3, 89.0, 63.7, 50.0, 37.3, 36.4, -0.6, -1.3.
HRMS ($C_{47}H_{44}N_6O_{20}S_2Si$): *m/z* (positive mode) = 1105.1897 (found $[M+H]^+$), 1105.1894 (calc.).

Compound 3cb

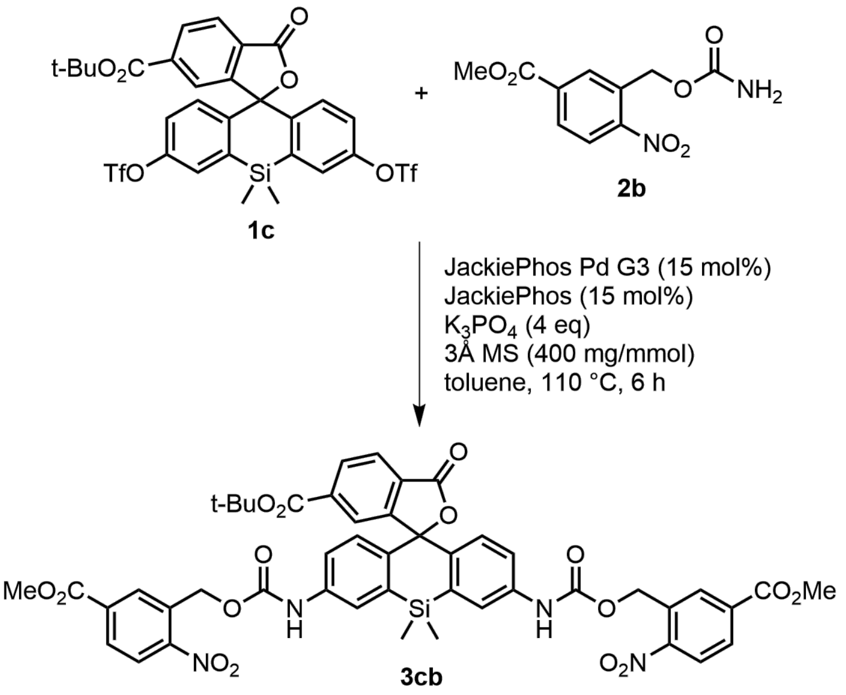

Prepared according to the general procedure 1 from 6'-(*tert*-butoxycarbonyl)silafluorescein ditriflate [14] (**1c**; 100 mg, 0.135 mmol), compound **2b** (83 mg, 0.325 mmol, 2.4 eq), anhydrous K_3PO_4 (115 mg, 0.54 mmol, 4 eq) and 3Å molecular sieves (55 mg), JackiePhos Pd G3 precatalyst (24 mg, 0.02 mmol, 15 mol%) and JackiePhos ligand (16 mg, 0.02 mmol, 15 mol%). Yield 85 mg (66%) of **3cb** as white solid.

^{1}H NMR (400 MHz, $CDCl_3$): δ 8.30 (t, J = 1.1 Hz, 2H), 8.15 – 8.09 (m, 5H), 7.99 (dd, J = 8.0, 0.7 Hz, 1H), 7.80 (br.s, 2H), 7.76 (dd, J = 1.3, 0.7 Hz, 1H), 7.30 (dd, J = 8.8, 2.5 Hz, 2H), 7.14 (br.s, 2H), 7.07 (d, J = 8.8 Hz, 2H), 5.60 (s, 4H), 3.95 (s, 6H), 1.53 (s, 9H), 0.70 (s, 3H), 0.58 (s, 3H).

^{13}C NMR (101 MHz, $CDCl_3$): δ 170.2, 165.1, 164.0, 155.2, 152.6, 150.0, 139.1, 137.8, 137.4, 135.9, 134.7, 132.7, 130.4, 130.1, 127.7, 127.4, 126.1, 125.3, 124.5, 123.7, 120.5, 89.8, 82.7, 63.4, 53.1, 28.2, -0.26, -0.34.

HRMS ($C_{47}H_{42}N_4O_{16}Si$): *m/z* (positive mode) = 947.2438 (found $[M+H]^+$), 947.2438 (calc.).

Compound 3cb-OH

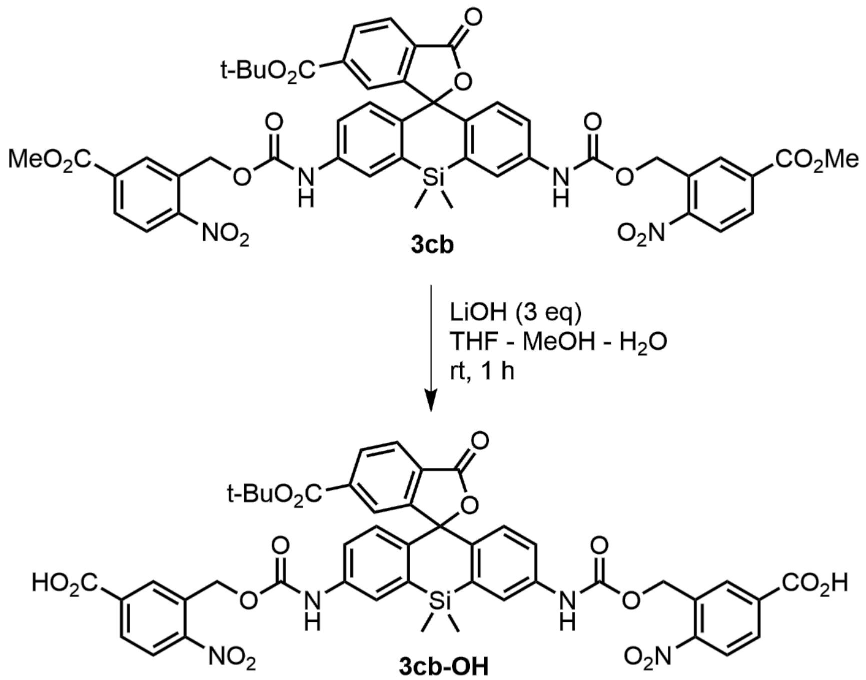

Prepared according to the general procedure 2 from a solution of compound **3cb** (40 mg, 42.2 µmol) in THF (400 µL) and methanol (200 µL) and a solution of $LiOH\cdot H_2O$ (5.3 mg, 127 µmol, 3 eq) in water (85 µL). Yield 30 mg (77%) of **3cb-OH** as white solid.

^{1}H NMR (400 MHz, acetone-d_6): δ 9.29 (br.s, 2H), 8.39 (dd, J = 1.8, 0.8 Hz, 2H), 8.25 (d, J = 8.3 Hz, 2H), 8.21 (dd, J = 8.3, 2.0 Hz, 2H), 8.17 (dd, J = 8.0, 1.3 Hz, 1H), 8.09 – 8.02 (m, 3H), 7.81 (d, J = 0.6 Hz, 1H), 7.57 (dd, J = 8.8, 2.5 Hz, 2H), 7.12 (d, J = 8.8 Hz, 2H), 5.63 (s, 4H), 1.53 (s, 9H), 0.75 (s, 3H), 0.61 (s, 3H).
^{13}C NMR (101 MHz, acetone-d_6): δ 170.1, 165.9, 164.5, 156.4, 153.8, 151.0, 139.7, 139.3, 138.5, 136.01, 135.95, 133.8, 131.03, 131.01, 130.9, 128.5, 128.0, 126.8, 126.1, 124.9, 124.2, 121.1, 90.1, 82.9, 63.5, 28.1, -0.2, -0.3.
HRMS ($C_{45}H_{38}N_4O_{16}Si$): *m/z* (positive mode) = 919.2124 (found $[M+H]^+$), 919.2125 (calc.).

Compound 3cb-SO_3H

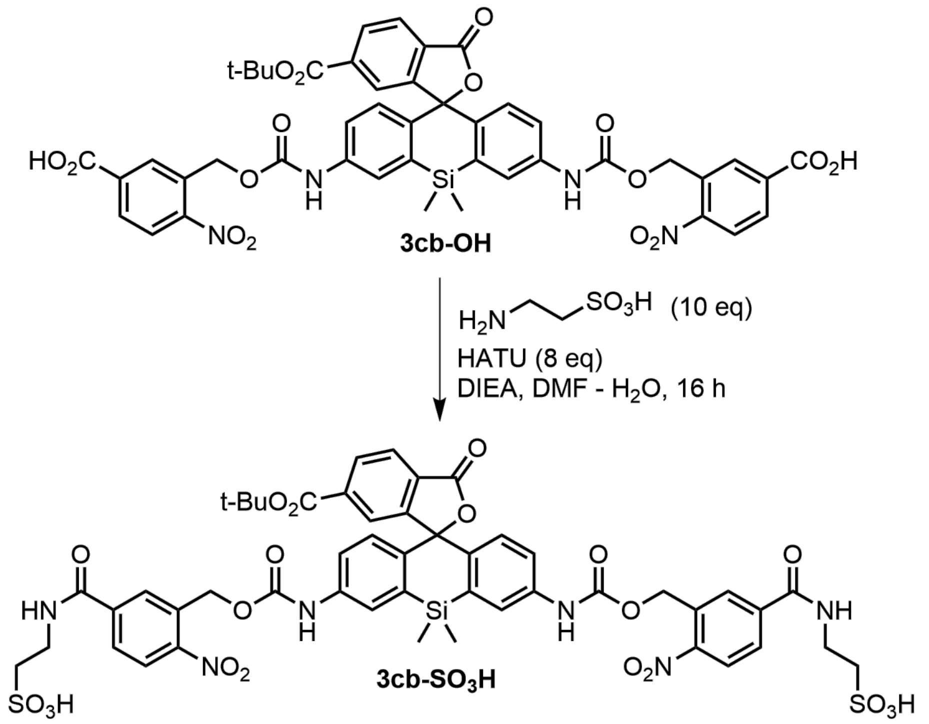

Prepared according to the general procedure 3 from compound **3cb-OH** (28 mg, 30.5 µmol) in DMF (500 µL) and water (150 µL), taurine (38 mg, 306 µmol, 10 eq), *N*,*N*-ethyldiisopropylamine (DIEA; 350 µL) and HATU (92 mg, 244 µmol, 8 eq; dissolved in 300 µL DMF). Yield 15.5 mg (45%) of **3cb-SO_3H** as pale violet solid.

^{1}H NMR (400 MHz, CD_3OD): δ 8.26 (br.s, 2H), 8.19 (d, J = 8.5 Hz, 2H), 8.15 (dd, J = 8.1, 1.3 Hz, 1H), 8.02 (dd, J = 8.1, 0.7 Hz, 1H), 7.95 (dd, J = 8.4, 1.8 Hz, 2H), 7.92 (d, J = 2.3 Hz, 2H), 7.74 (app.t, J = 1.0 Hz, 1H), 7.49 (dd, J = 8.8, 2.4 Hz, 2H), 7.00 (d, J = 8.8 Hz, 2H), 5.58 (s, 4H), 3.82 (br.t, J = 6.4 Hz, 4H), 3.70 (hept, J = 6.6 Hz, DIEA-H^+), 3.18 (q, J = 7.4 Hz, DIEA-H^+), 3.13 (br.t, J = 6.4 Hz, 4H), 1.53 (s, 9H), 1.36 – 1.30 (m, DIEA-H^+), 0.73 (s, 3H), 0.62 (s, 3H).
^{13}C NMR (101 MHz, CD_3OD): δ 171.61, 167.58, 165.2, 157.0, 154.9, 150.2, 140.5, 140.0, 139.4, 139.0, 136.7, 134.4, 131.3, 129.1, 128.9, 128.6, 128.0, 127.0, 126.3, 125.3, 124.7, 121.4, 91.3, 83.7, 64.1, 55.8, 51.1, 43.8, 37.4, 28.3, 18.7, 17.3, 13.2, -0.25, -0.27 (including the signals of DIEA-H^+ counterion).
HRMS ($C_{49}H_{48}N_6O_{20}S_2Si$): *m/z* (positive mode) = 1133.2208 (found $[M+H]^+$), 1133.2207 (calc.).

Caged dye 4cb

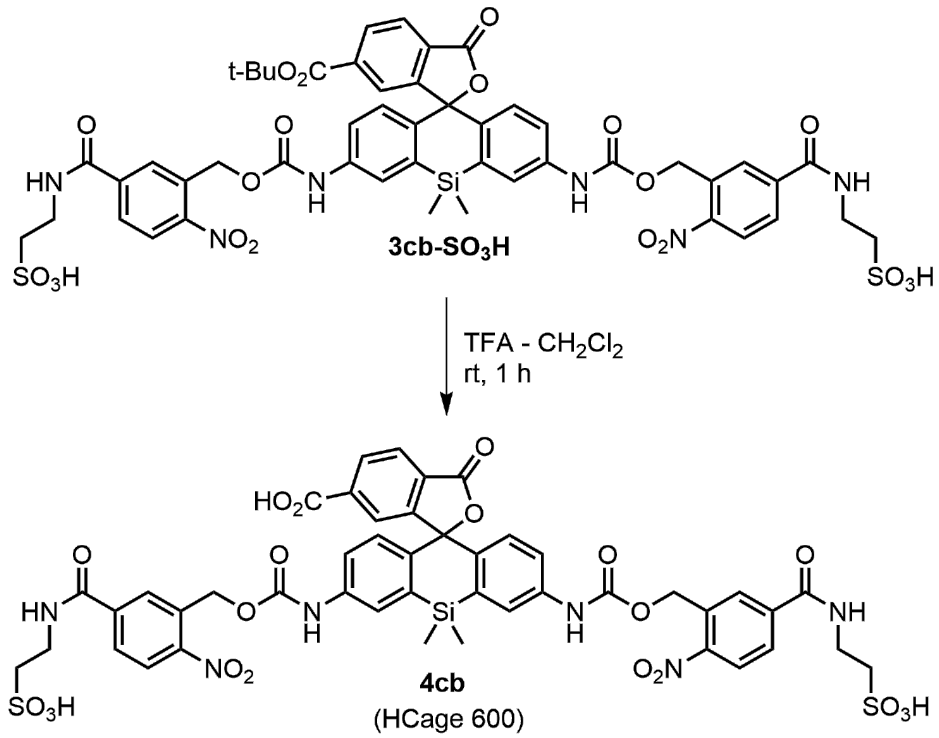

Prepared according to the general procedure 4 from compound **3cb-SO$_3$H** (15 mg, 13.3 µmol) in CH_2Cl_2 (600 µL) and TFA (300 µL). Yield 9.3 mg (65%) of **4cb** as light violet solid.

^{1}H NMR (400 MHz, DMSO-d$_6$): δ 10.08 (s, 2H), 8.84 (t, J = 5.3 Hz, 2H), 8.28 – 8.20 (m, 4H), 8.14 (dd, J = 8.0, 1.3 Hz, 1H), 8.09 (d, J = 7.7 Hz, 1H), 8.01 – 7.92 (m, 4H), 7.72 (s, 1H), 7.52 (dd, J = 8.8, 2.4 Hz, 2H), 6.99 (d, J = 8.8 Hz, 2H), 5.53 (s, 4H), 3.57 (q, J = 6.9 Hz, 4H), 2.76 (t, J = 6.9 Hz, 4H), 0.66 (s, 3H), 0.56 (s, 3H).

^{13}C NMR (101 MHz, DMSO-d$_6$): δ 169.1, 165.9, 164.0, 154.7, 152.8, 148.4, 139.2, 138.7, 137.4, 136.8, 134.9, 132.5, 130.4, 128.3, 127.4, 127.2, 126.9, 126.4, 125.3, 124.0, 123.3, 120.1, 89.2, 62.6, 50.0, 36.4, -0.5, -0.9.

HRMS ($C_{45}H_{40}N_6O_{20}S_2Si$): *m/z* (positive mode) = 1077.1583 (found [M+H]$^+$), 1077.1581 (calc.).

Synthesis of photoactivatable labels

HCage 520 NHS ester (4aa-NHS)

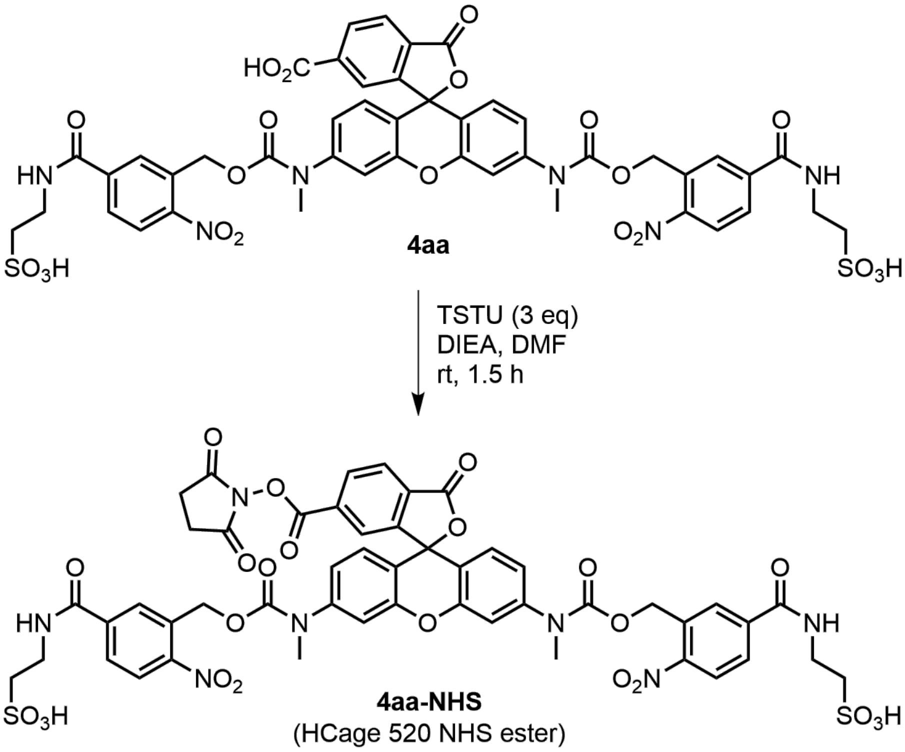

A solution of compound **4aa** (8 mg, 7.5 µmol) and *N*,*N*-ethyldiisopropylamine (DIEA; 60 µL) in DMF (150 µL) was treated with *N*,*N*,*N'*,*N'*-tetramethyl-*O*-(*N*-succinimidyl)uronium tetrafluoroborate (TSTU; 6.8 mg, 22.6 µmol in 50 µL DMF). The reaction mixture was stirred at rt for 1.5 h, the solvents were evaporated to dryness and the residue was dried in vacuo. The product was isolated by preparative HPLC (column: Interchim 250×21.2 mm 5 µm Uptisphere Strategy PhC4; gradient 20/80 → 60/40 A:B, A = 0.1% v/v HCO_2H in acetonitrile, B = 0.1% v/v HCO_2H in water; detection at 220 nm), fractions containing the product were evaporated (bath temperature 30 °C), and the residue was freeze-dried from aq. dioxane to give **4aa-NHS** as light orange solid (7 mg, 81%).

HRMS ($C_{49}H_{41}N_7O_{23}S_2$): *m/z* (positive mode) = 1160.1767 (found $[M+H]^+$), 1160.1768 (calc.).

HCage 580 NHS ester (4ba-NHS)

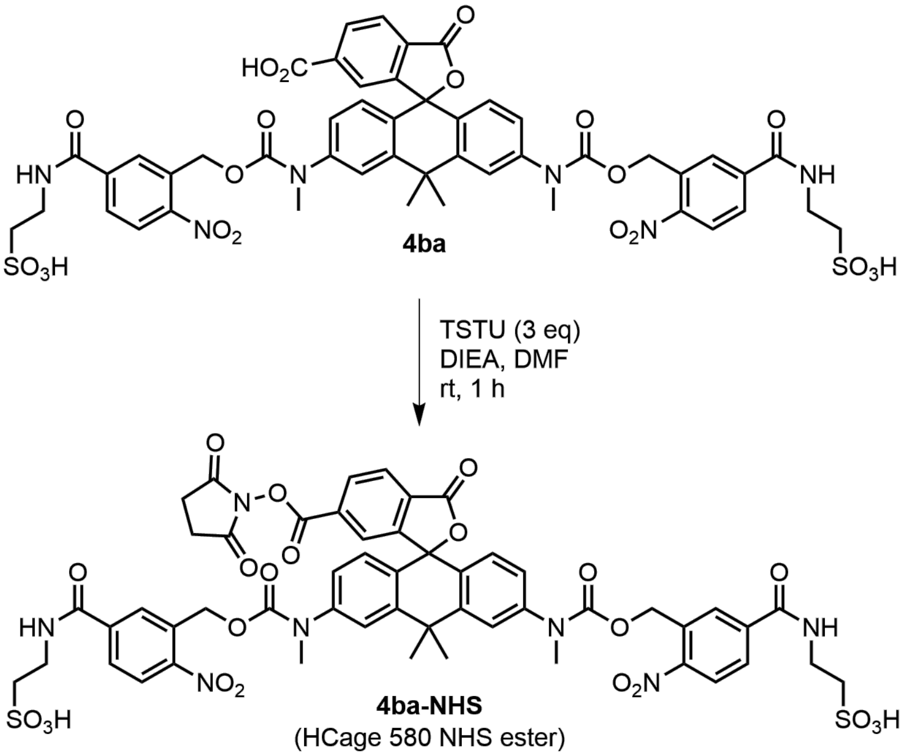

A solution of compound **4ba** (7 mg, 6.43 μmol) and *N*,*N*-ethyldiisopropylamine (DIEA; 50 μL) in DMF (150 μL) was treated with *N*,*N*,*N'*,*N'*-tetramethyl-*O*-(*N*-succinimidyl)uronium tetrafluoroborate (TSTU; 5.8 mg, 19.4 μmol in 50 μL DMF). The reaction mixture was stirred at rt for 1 h, the solvents were evaporated to dryness and the residue was dried in vacuo. The product was isolated by preparative HPLC (column: Interchim 250×21.2 mm 5 μm Uptisphere Strategy PhC4; gradient 20/80 → 60/40 A:B, A = 0.1% v/v HCO_2H in acetonitrile, B = 0.1% v/v HCO_2H in water; detection at 220 nm), fractions containing the product were evaporated (bath temperature 30 °C), and the residue was freeze-dried from aq. dioxane to give **4ba-NHS** as light pink solid (9.3 mg, ~100%, bis-DIEA salt).

HRMS ($C_{52}H_{47}N_7O_{22}S_2$): *m/z* (positive mode) = 1186.2287 (found $[M+H]^+$), 1186.2288 (calc.).

HCage 600 NHS ester (4cb-NHS)

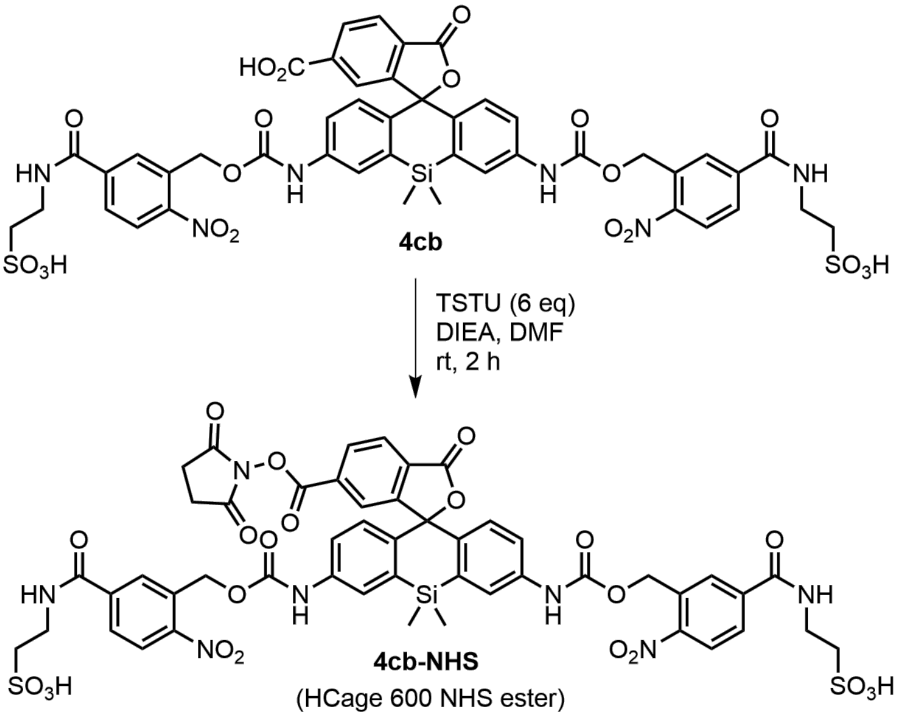

A solution of compound **4cb** (6.5 mg, 4.6 µmol) and *N*,*N*-ethyldiisopropylamine (DIEA; 50 µL) in DMF (150 µL) was treated with *N*,*N*,*N′*,*N′*-tetramethyl-*O*-(*N*-succinimidyl)uronium tetrafluoroborate (TSTU; 4.2 mg, 14 µmol in 40 µL DMF). The reaction mixture was stirred at rt for 1 h, at which time the LC/MS analysis showed incomplete conversion, so another portion of TSTU (4.2 mg, 14 µmol in 40 µL DMF) was added and the mixture was stirred for another 1 h period. The solvents were evaporated to dryness and the residue was dried in vacuo. The product was isolated by preparative HPLC (column: Interchim 250×21.2 mm 5 µm Uptisphere Strategy PhC4; gradient 20/80 → 60/40 A:B, A = 0.1% v/v HCO_2H in acetonitrile, B = 0.1% v/v HCO_2H in water; detection at 220 nm), fractions containing the product were evaporated (bath temperature 30 °C), and the residue was freeze-dried from aq. dioxane to give **4cb-NHS** as light violet solid (7.2 mg, ~100%, bis-DIEA salt).

HRMS ($C_{49}H_{43}N_7O_{22}S_2Si$): *m/z* (positive mode) = 1174.1745 (found $[M+H]^+$), 1174.1745 (calc.).

HCage 620 NHS ester (4ca-NHS)

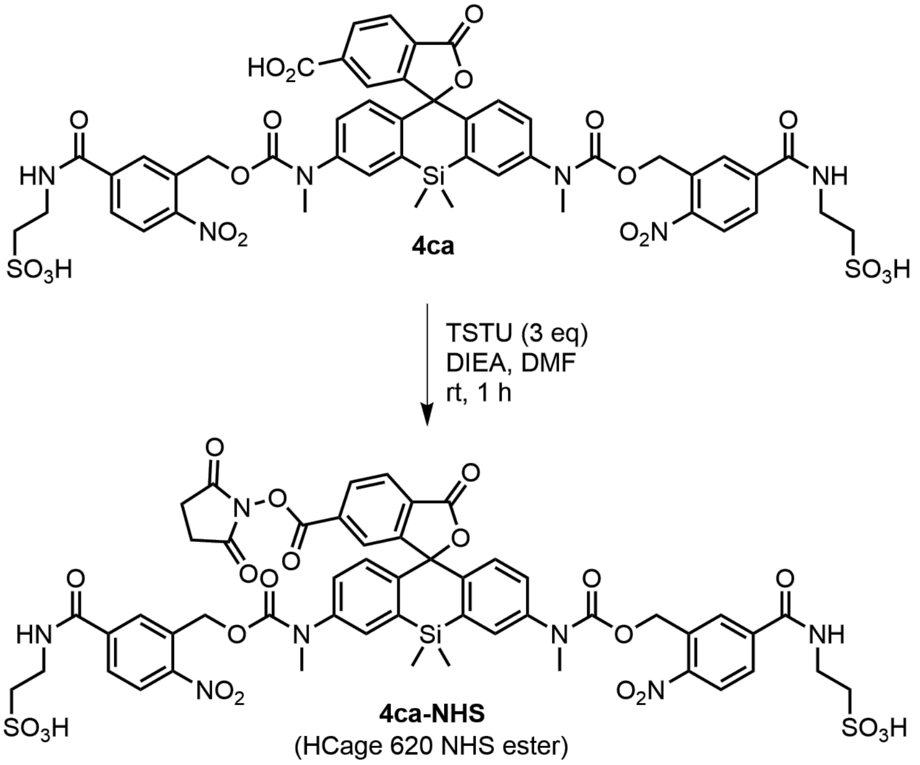

A solution of compound **4ca** (4.4 mg, 4 µmol) and *N*,*N*-ethyldiisopropylamine (DIEA; 40 µL) in DMF (150 µL) was treated with *N*,*N*,*N′*,*N′*-tetramethyl-*O*-(*N*-succinimidyl)uronium tetrafluoroborate (TSTU; 3.7 mg, 12 µmol in 40 µL DMF). The reaction mixture was stirred at rt for 1 h, the solvents were evaporated to dryness and the residue was dried in vacuo. The product was isolated by preparative HPLC (column: Interchim 250×21.2 mm 5 µm Uptisphere Strategy PhC4; gradient 20/80 → 60/40 A:B, A = 0.1% v/v HCO_2H in acetonitrile, B = 0.1% v/v HCO_2H in water; detection at 220 nm), fractions containing the product were evaporated (bath temperature 30 °C), and the residue was freeze-dried from aq. dioxane to give **4ca-NHS** as white solid (3.5 mg, 73%).

HRMS ($C_{51}H_{45}N_7O_{22}S_2Si$): *m/z* (positive mode) = 1202.2062 (found $[M+H]^+$), 1202.2058 (calc.).

HCage 620 maleimide (4ca-maleimide)

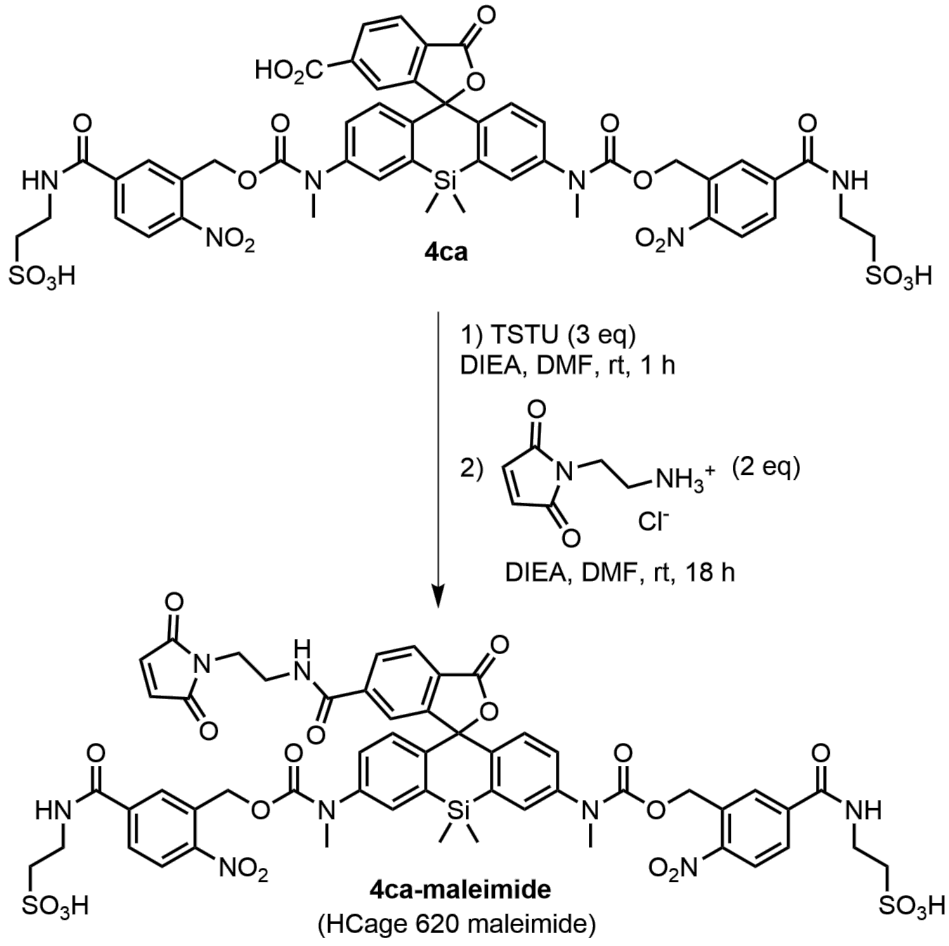

A solution of compound **4ca** (5 mg, 4.53 µmol) and *N*,*N*-ethyldiisopropylamine (DIEA; 45 µL) in DMF (100 µL) was treated with *N*,*N*,*N′*,*N′*-tetramethyl-*O*-(*N*-succinimidyl)uronium tetrafluoroborate (TSTU; 4.1 mg, 13.6 µmol in 40 µL DMF). The reaction mixture was stirred at rt for 1 h, and DIEA (45 µL) was added followed by the solution of 1-(2-aminoethyl)maleimide hydrochloride (1.6 mg, 9.06 µmol in 30 µL DMF). The mixture was left stirring at rt overnight (18 h), the solvents were then evaporated to dryness and the residue was dried in vacuo. The product was isolated by preparative HPLC (column: Interchim 250×21.2 mm 5 µm Uptisphere Strategy PhC4; gradient 20/80 → 60/40 A:B, A = acetonitrile, B = 50 mM triethylammonium bicarbonate in water, pH = 7.0-7.5; detection at 220 nm), fractions containing the product were evaporated (bath temperature 30 °C), and the residue was freeze-dried from aq. dioxane to give **4ca-maleimide** as yellowish solid (3.8 mg, 68%).

HRMS ($C_{53}H_{50}N_8O_{21}S_2Si$): *m/z* (positive mode) = 1227.2375 (found $[M+H]^+$), 1227.2374 (calc.).

HCage 600 HaloTag ligand (4cb-Halo)

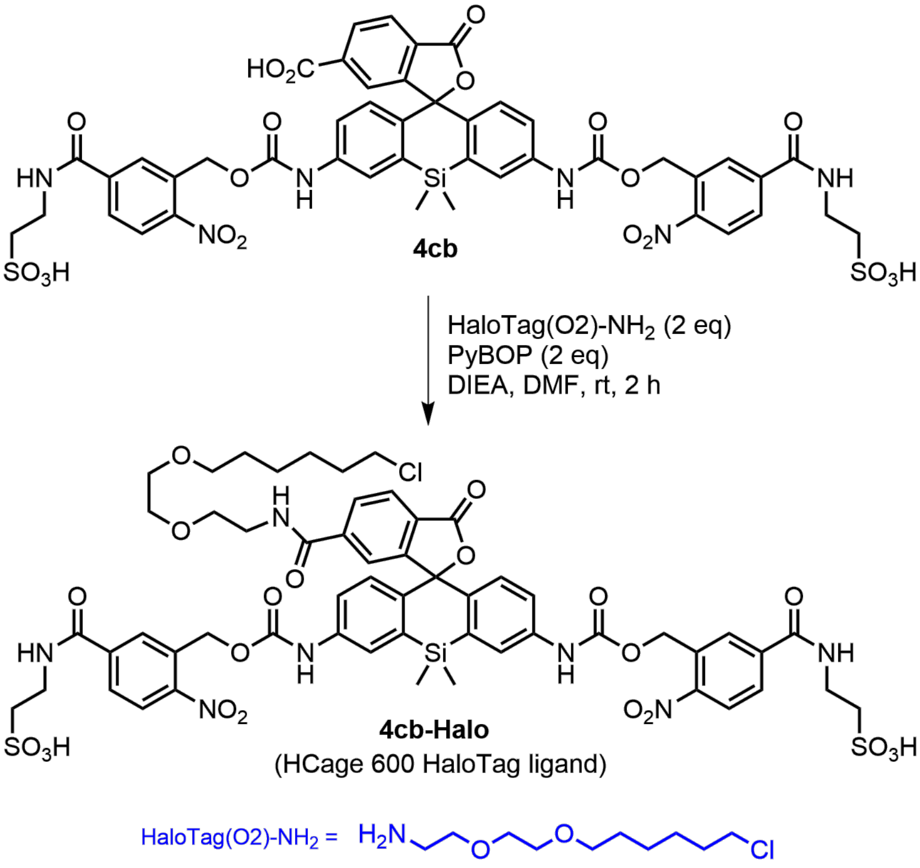

A solution of compound **4cb** (4 mg, 3.71 µmol) and *N*,*N*-ethyldiisopropylamine (DIEA; 30 µL) in DMF (100 µL) was treated with HaloTag(O2)-NH_2 ligand [15] (1.7 mg, 7.43 µmol in 25 µL DMF) and (benzotriazol-1-yloxy)tripyrrolidinophosphonium hexafluorophosphate (PyBOP; 3.9 mg, 7.43 µmol in 30 µL DMF). The resulting light yellow clear solution was stirred at rt for 2 h, the solvents were then evaporated to dryness and the residue was dried in vacuo. The product was isolated by preparative HPLC (column: Interchim 250×21.2 mm 5 µm Uptisphere Strategy PhC4; gradient 20/80 → 70/30 A:B, A = acetonitrile, B = 50 mM ammonium formate in water, pH = 3.5-4.0; detection at 220 nm), fractions containing the product were evaporated (bath temperature 30 °C), and the residue was freeze-dried from aq. dioxane to give **4cb-Halo** as white solid (4 mg, 84%).

HRMS ($C_{55}H_{60}ClN_7O_{21}S_2Si$): *m/z* (positive mode) = 1282.2810 (found $[M+H]^+$), 1282.2814 (calc.).

HCage 620 HaloTag ligand (4ca-Halo)

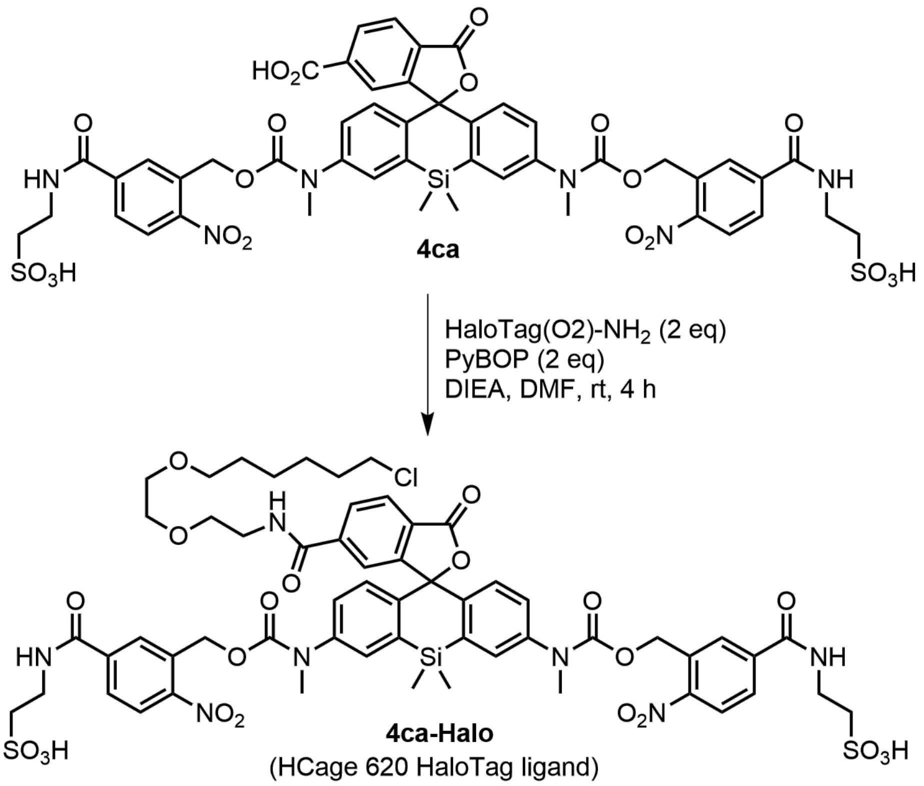

A solution of compound **4ca** (5 mg, 4.53 µmol) and *N*,*N*-ethyldiisopropylamine (DIEA; 30 µL) in DMF (100 µL) was treated with HaloTag(O2)-NH_2 ligand (2 mg, 9.1 µmol in 25 µL DMF) and (benzotriazol-1-yloxy)tripyrrolidinophosphonium hexafluorophosphate (PyBOP; 4.7 mg, 9.1 µmol in 30 µL DMF). The resulting light yellow clear solution was stirred at rt for 4 h, the solvents were then evaporated to dryness and the residue was dried in vacuo. The product was isolated by preparative HPLC (column: Interchim 250×21.2 mm 5 µm Uptisphere Strategy PhC4; gradient 20/80 → 70/30 A:B, A = acetonitrile, B = 50 mM ammonium formate in water, pH = 3.5-4.0; detection at 220 nm), fractions containing the product were evaporated (bath temperature 30 °C), and the residue was freeze-dried from aq. dioxane to give **4ca-Halo** as white solid (4.9 mg, 83%).

HRMS ($C_{57}H_{64}ClN_7O_{21}S_2Si$): *m/z* (positive mode) = 1310.3126 (found $[M+H]^+$), 1310.3127 (calc.).

HCage 620 SNAP-tag ligand (4ca-BG)

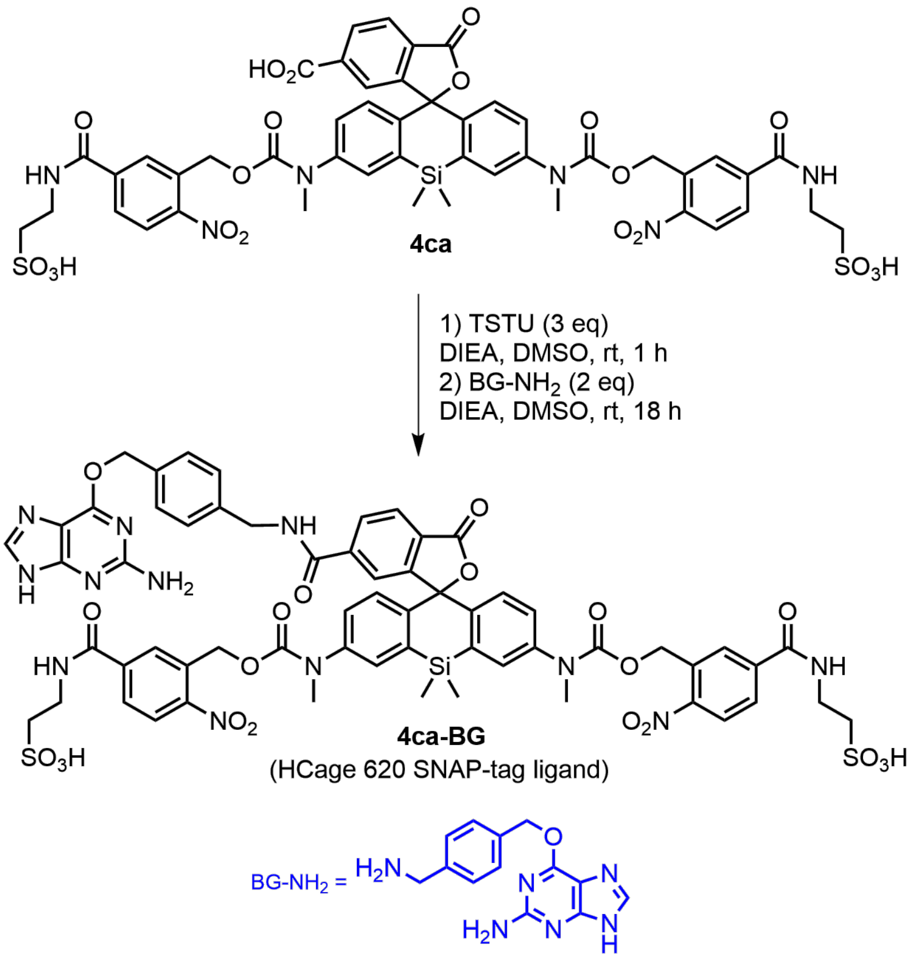

A solution of compound **4ca** (5 mg, 4.53 µmol) and *N*,*N*-ethyldiisopropylamine (DIEA; 45 µL) in DMSO (100 µL) was treated with *N*,*N*,*N'*,*N'*-tetramethyl-*O*-(*N*-succinimidyl)uronium tetrafluoroborate (TSTU; 4.1 mg, 13.6 µmol in 40 µL DMF). The reaction mixture was stirred at rt for 1 h, and DIEA (45 µL) was added followed by the solution of 6-((4-(aminomethyl)benzyloxy)-7*H*-purin-2-amine [16] (BG-NH$_2$; 2.4 mg, 9.06 µmol in 70 µL DMSO). The mixture was left stirring at rt overnight (18 h), the solvents were then evaporated to dryness and the residue was dried in vacuo. The product was isolated by preparative HPLC (column: Interchim 250×21.2 mm 5 µm Uptisphere Strategy PhC4; gradient 20/80 → 60/40 A:B, A = acetonitrile, B = 50 mM triethylammonium bicarbonate in water, pH = 7.0-7.5; detection at 220 nm), fractions containing the product were evaporated (bath temperature 30 °C), and the residue was freeze-dried from aq. dioxane to give **4ca-BG** as white solid (3.2 mg, 52%).

HRMS ($C_{60}H_{56}N_{12}O_{20}S_2Si$): *m/z* (positive mode) = 1357.3021 (found $[M+H]^+$), 1357.3017 (calc.).

Preparation of 600SiR dye and 600SiR-Halo ligand

Compound S2

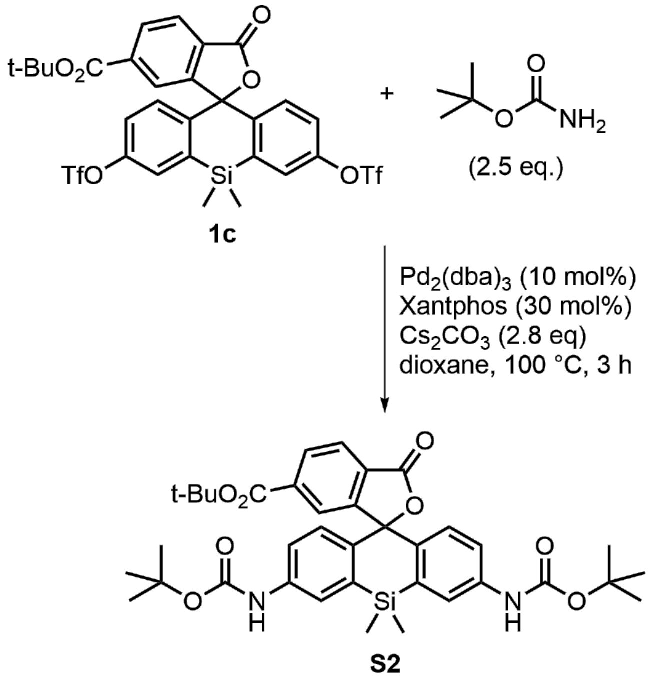

6'-(*tert*-Butoxycarbonyl)silafluorescein ditriflate [14] (**1c**; 148 mg, 0.2 mmol), *tert*-butyl carbamate (59 mg, 0.5 mmol, 2.5 eq), Cs_2CO_3 (183 mg, 0.56 mmol, 2.8 eq), $Pd_2(dba)_3$ (18 mg, 0.02 mmol, 10 mol%) and Xantphos ligand (35 mg, 0.06 mmol, 30 mol%) were loaded in a dry 10 mL tube, anhydrous 1,4-dioxane (1.5 mL) was added, the reaction mixture was degassed and stirred at 100 °C (bath temperature) for 3 h. Afterwards, the mixture was diluted with EtOAc, poured into brine (40 mL), extracted with EtOAc (3×25 mL), the combined extracts were dried over Na_2SO_4, filtered and evaporated on Celite. The product was isolated by flash chromatography on Biotage Isolera system (12 g Interchim SiHP 30 µm cartridge, gradient 10% to 50% EtOAc/hexane) and freeze-dried from dioxane to yield 94 mg (70%) of **S2** as white solid, containing ~30 mol% *tert*-butyl carbamate, which was used in the next step without further purification.

^{1}H NMR (400 MHz, $CDCl_3$): δ 8.11 (dd, J = 8.0, 1.3 Hz, 1H), 7.97 (dd, J = 8.1, 0.7 Hz, 1H), 7.77 – 7.70 (m, 3H), 7.23 (dd, J = 8.8, 2.5 Hz, 2H), 7.04 (d, J = 8.8 Hz, 2H), 6.62 (s, 2H), 1.53 (s, 9H), 1.51 (s, 18H), 0.71 (s, 3H), 0.60 (s, 3H).
^{13}C NMR (101 MHz, $CDCl_3$): δ 170.4, 164.1, 155.6, 152.7, 138.24, 138.19, 137.7, 135.6, 130.3, 127.7, 127.3, 126.0, 124.5, 123.3, 120.2, 90.0, 82.6, 81.0, 67.2, 28.5, 28.2, -0.16, -0.17.
HRMS ($C_{37}H_{44}N_2O_8Si$): *m/z* (positive mode) = 673.2938 (found $[M+H]^+$), 673.2940 (calc.).

600SiR dye

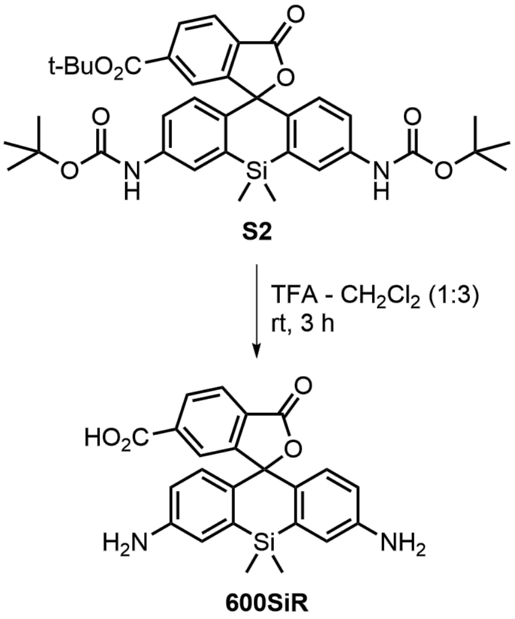

To the solution of crude **S2** (90 mg, 0.134 mmol) in CH_2Cl_2 (3 mL), trifluoroacetic acid (1 mL) was added, and the resulting red fluorescent solution was stirred at rt for 3 h. The reaction mixture was diluted with toluene and evaporated to dryness, and the product was isolated by flash chromatography on Biotage Isolera system (24 g Interchim NH2 30 µm cartridge, gradient 0% to 40% A/B, A = 0.1% HCO_2H in water, B = 0.1% HCO_2H in acetonitrile, flow rate 25 mL/min) and freeze-dried from dioxane to yield 55 mg (81%) of **600SiR** as light blue solid, containing 1 eq./eq. 1,4-dioxane.

^1H NMR (400 MHz, CD_3CN): δ 8.15 (dd, J = 8.0, 1.3 Hz, 1H), 7.96 (dd, J = 8.0, 0.8 Hz, 1H), 7.77 (dd, J = 1.3, 0.7 Hz, 1H), 7.00 (d, J = 2.6 Hz, 2H), 6.65 (d, J = 8.6 Hz, 2H), 6.52 (dd, J = 8.6, 2.6 Hz, 2H), 3.70 (br.s, 4H), 0.57 (s, 3H), 0.48 (s, 3H).
^{13}C NMR (101 MHz, CD_3CN): δ 170.5, 166.8, 156.1, 148.6, 137.3, 136.9, 133.1, 131.1, 130.3, 129.0, 126.6, 126.0, 120.1, 116.7, 92.4, 0.0, -1.3.
HRMS ($C_{23}H_{20}N_2O_4Si$): *m/z* (positive mode) = 417.1266 (found $[M+H]^+$), 417.1265 (calc.).
UV-Vis (PBS, pH 7.4): absorption, λ_{max}: 590 nm; emission, λ_{max} (Φ): 611 nm (0.51), excitation at 540 nm.
UV-Vis (0.1% TFA in ethanol): absorption, λ_{max} (ε): 608 nm (68000 $M^{-1}cm^{-1}$); emission, λ_{max} (Φ): 626 nm (0.59), excitation at 540 nm.

600SiR-Halo ligand

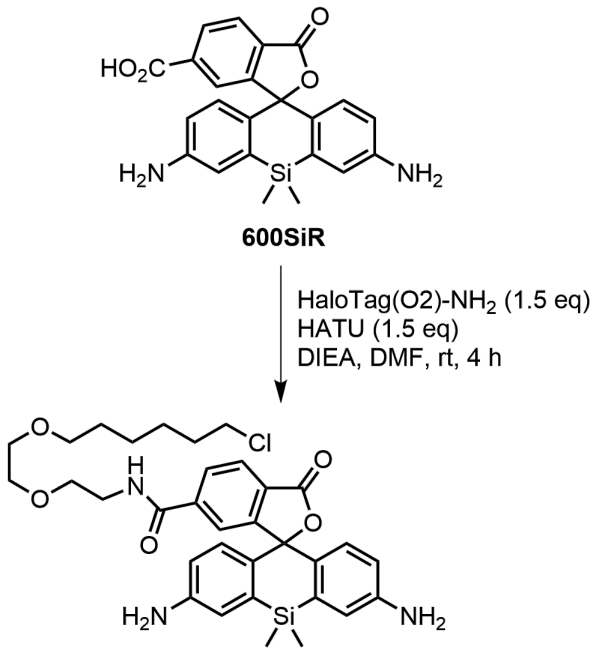

600SiR-Halo

A solution of **600SiR** dye (10 mg, 24 µmol) and *N*,*N*-ethyldiisopropylamine (DIEA; 100 µL) in DMF (100 µL) was treated with HaloTag(O2)-NH_2 ligand (8.1 mg, 36 µmol in 100 µL DMF) and 1-[bis(dimethylamino)methylene]-1*H*-1,2,3-triazolo[4,5-*b*]pyridinium 3-oxid hexafluorophosphate (HATU; 14 mg, 36 µmol in 100 µL DMF). The resulting solution was stirred at rt for 2 h, the solvents were then evaporated to dryness and the residue was dried in vacuo. The product was isolated by preparative HPLC (column: ThermoScientific 250×21.2 mm 5 µm Hypersil Gold C18; gradient 30/70 → 70/30 A:B, A = 0.1% HCO_2H in acetonitrile, B = 0.1% HCO_2H in water; detection at 220 and 600 nm), fractions containing the product were evaporated (bath temperature 40 °C), and the residue was freeze-dried from dioxane to give **600SiR-Halo** as white solid (8.5 mg, 57%).

HRMS ($C_{33}H_{40}ClN_3O_5Si$): *m/z* (positive mode) = 622.2494 (found $[M+H]^+$), 622.2499 (calc.).

Supplementary references

[1] Wilhelm, J.; Kühn, S.; Tarnawski, M.; Gotthard, G.; Tünnermann, J.; Tänzer, T.; Karpenko, J.; Mertes, N.; Xue, L.; Uhrig, U.; Reinstein, J.; Hiblot, J.; Johnsson, K. Kinetic and structural characterization of the self-labeling protein tags HaloTag7, SNAP-tag, and CLIP-tag. *Biochemistry* **2021**, *60(33)*, 2560–2575.

[2] Schnitzbauer, J.; Strauss, M.T.; Schlichthaerle, T.; Schueder, F.; Jungmann, R. Super-resolution microscopy with DNA-PAINT. *Nat. Protoc.* **2017**, *12*, 1198–1228.

[3] Butkevich, A.N.; Ta, H.; Ratz, M.; Stoldt, S.; Jakobs, S.; Belov, V.N.; Hell, S.W. Two-color 810 nm STED nanoscopy of living cells with endogenous SNAP-tagged fusion proteins. *ACS Chem. Biol.* **2018**, *13(2)*, 475–480.

[4] Thevathasan, J.V.; Kahnwald, M.; Cieśliński, K.; Hoess, P.; Peneti, S.K.; Reitberger, M.; Heid, D.; Kasuba, K.C.; Hoerner, S.J.; Li, Y.; Wu, Y.-L.; Mund, M.; Matti, U.; Pereira, P.M.; Henriques, R.; Nijmeijer, B.; Kueblbeck, M.; Sabinina, V.J.; Ellenberg, J.; Ries, J. Nuclear pores as versatile reference standards for quantitative superresolution microscopy. *Nat. Methods* **2019**, *16(10)*, 1045–1053.

[5] Otsuka, S.; Bui, K.H.; Schorb, M.; Hossain, M.J.; Politi, A.Z.; Koch, B.; Eltsov, M.; Beck, M.; Ellenberg, J. Nuclear pore assembly proceeds by an inside-out extrusion of the nuclear envelope. *eLife* **2016**, *5*, e19071.

[6] D'Este, E.; Kamin, D.; Gottfert, F.; El-Hady, A.; Hell, S.W. STED nanoscopy reveals the ubiquity of subcortical cytoskeleton periodicity in living neurons. *Cell Rep.* **2015**, *10(8)*, 1246–1251.

[7] Gwosch, K.C.; Pape, J.K.; Balzarotti, F.; Hoess, P.; Ellenberg, J.; Ries, J.; Hell, S.W. MINFLUX nanoscopy delivers 3D multicolor nanometer resolution in cells. *Nat. Methods* **2020**, *17*, 217–224.

[8] Weber, M.; Leutenegger, M.; Stoldt, S.; Jakobs, S.; Mihaila, T.S.; Butkevich, A.N.; Hell, S.W. MINSTED fluorescence localization and nanoscopy. *Nat. Photonics* **2021**, *15*, 361–366.

[9] Arganda-Carreras, I.; Sorzano, C.O.S.; Marabini, R.; Carazo, J.M.; Ortiz-de-Solorzano, C.; Kybic, J. Consistent and elastic registration of histological sections using vector-spline regularization. In: Beichel R.R., Sonka M. (eds) Computer Vision Approaches to Medical Image Analysis. CVAMIA 2006. Lecture Notes in Computer Science, **2006**, vol 4241. Springer, Berlin, Heidelberg. DOI: 10.1007/11889762_8.

[10] Kaushik, S.; Prokop, Z.; Damborsky, J.; Chaloupkova, R. Kinetics of binding of fluorescent ligands to enzymes with engineered access tunnels. *FEBS J.* **2017**, *284(1)*, 134–148.

[11] Morin, M.D.; Wang, Y.; Jones, B.T.; Su, L.; Surakattula, M.M.R.P.; Berger, M.; Huang, H.; Beutler, E.K.; Zhang, H.; Beutler, B.; Boger, D.L. Discovery and structure-activity relationships of the neoseptins: a new class of toll-like receptor-4 (TLR4) agonists. *J. Med. Chem.* **2016**, *59(10)*, 4812–4830.

[12] Grimm, J.B.; English, B.P.; Chen, J.; Slaughter, J.P.; Zhang, Z.; Revyakin, A.; Patel, R.; Macklin, J.J.; Normanno, D.; Singer, R.H.; Lionnet, T.; Lavis, L.D. A general method to improve fluorophores for live-cell and single-molecule microscopy. *Nat. Methods* **2015**, *12*, 244–250.
[13] Butkevich, A. N.; Mitronova, G. Y.; Sidenstein, S. C.; Klocke, J. L.; Kamin, D.; Meineke, D. N. H.; D'Este, E.; Kraemer, P.-T.; Danzl, J. G.; Belov, V. N.; Hell, S. W. Fluorescent rhodamines and fluorogenic carbopyronines for super-resolution STED microscopy in living cells. *Angew. Chem. Int. Ed.* **2016**, *55*, 3290-3294.
[14] Butkevich, A. N.; Belov, V. N.; Kolmakov, K.; Sokolov, V. V.; Shojaei, H.; Sidenstein, S. C.; Kamin, D.; Matthias, J.; Vlijm, R.; Engelhardt, J.; Hell, S. W. Hydroxylated fluorescent dyes for live-cell labeling: synthesis, spectra and super-resolution STED microscopy. *Chem. Eur. J.* **2017**, *23*, 12114–12119.
[15] Singh, V.; Wang, S.; Kool, E.T. Genetically encoded multispectral labeling of proteins with polyfluorophores on a DNA backbone. *J. Am. Chem. Soc.* **2013**, *135(16)*, 6184–6191.
[16] Kobayashi, T.; Komatsu, T.; Kamiya, M.; Campos, C.; González-Gaitán, M.; Terai, T.; Hanaoka, K.; Nagano, T.; Urano, Y. Highly activatable and environment-insensitive optical highlighters for selective spatiotemporal imaging of target proteins. *J. Am. Chem. Soc.* **2012**, *134(27)*, 11153–11160.

NMR spectra

1H (400.15 MHz, CDCl3)

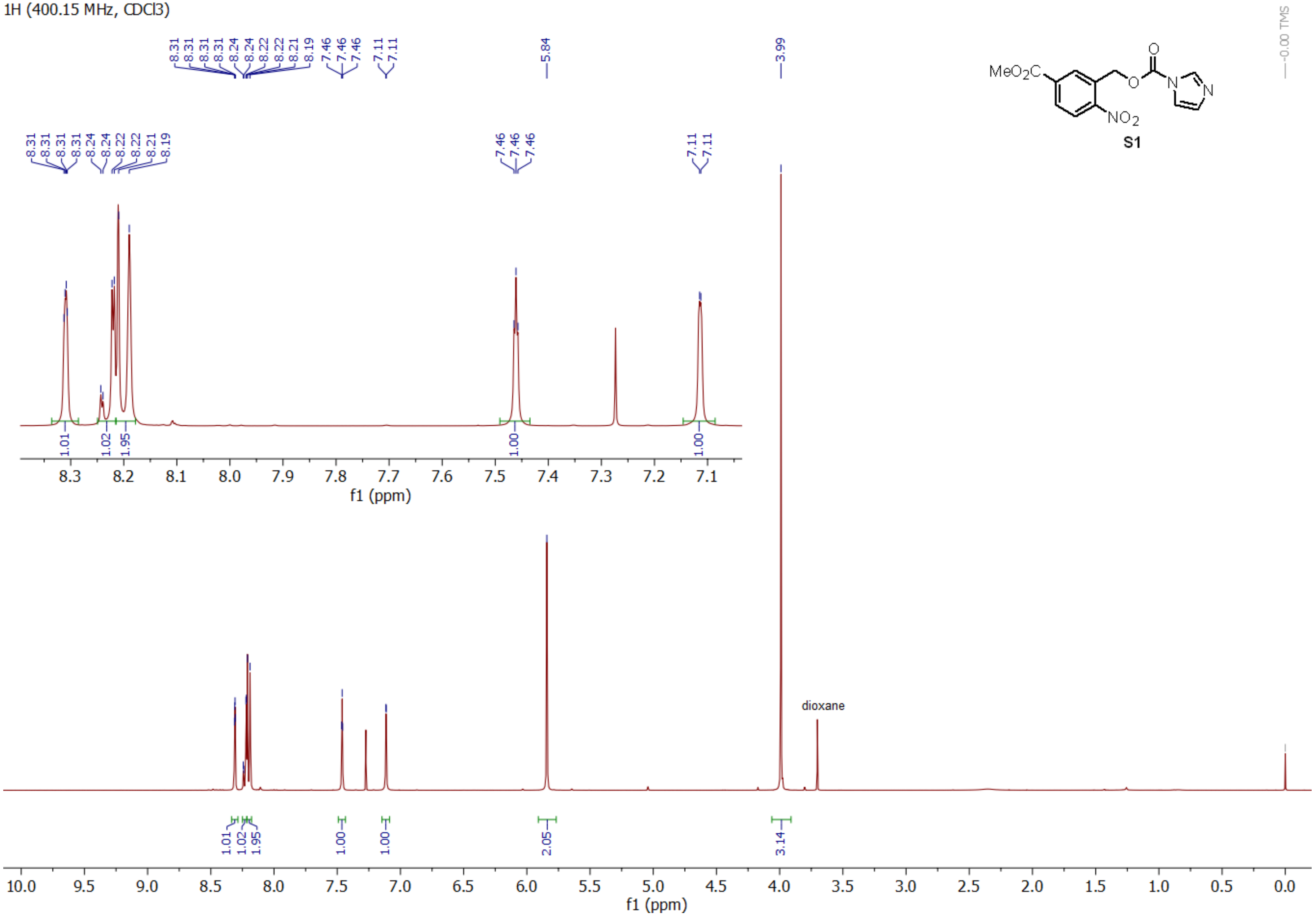

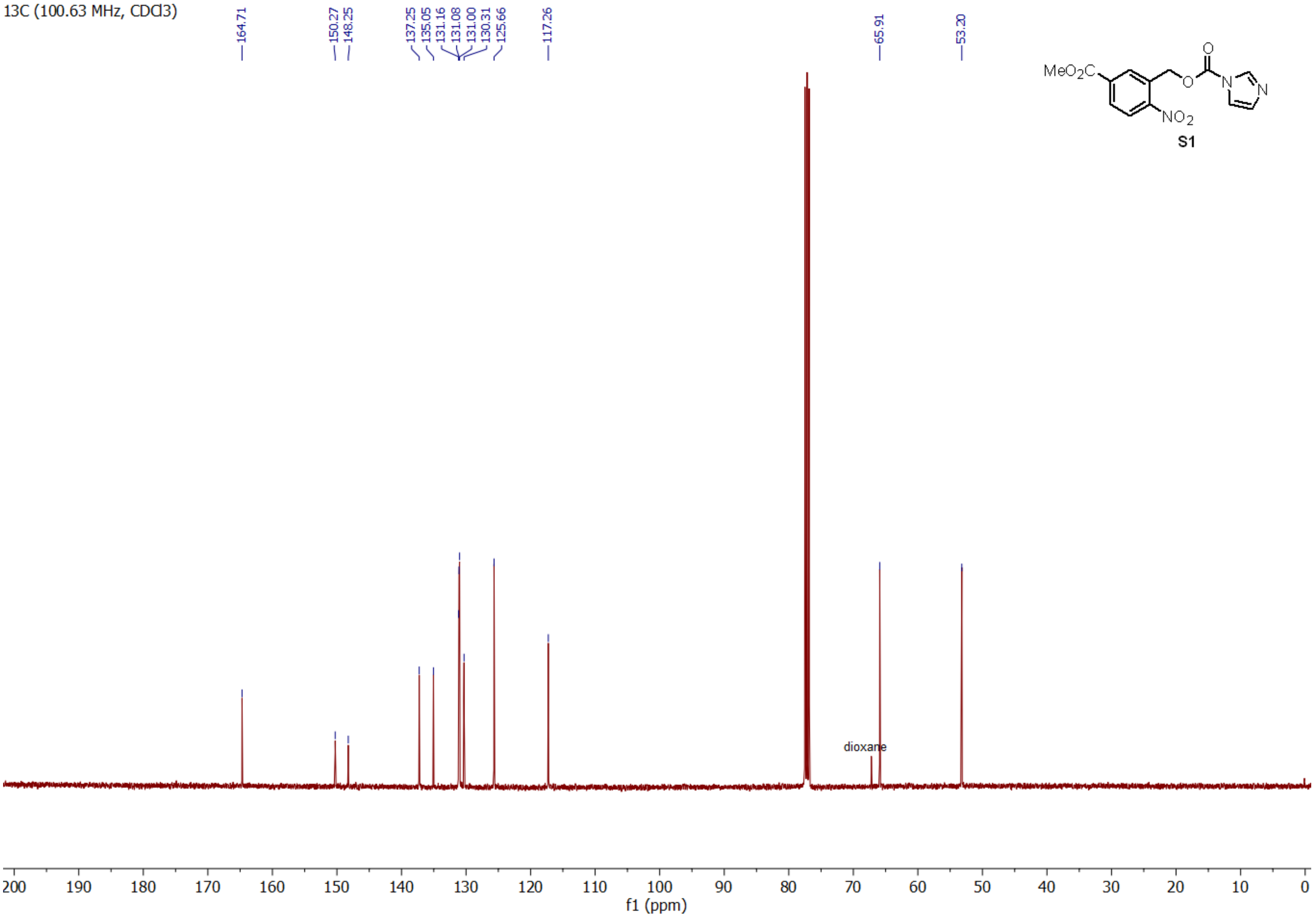
13C (100.63 MHz, CDCl3)
164.71
150.27
148.25
137.25
135.05
131.16
131.08
131.00
130.31
125.66
117.26
65.91
53.20
MeO2C
O
O
N
N
NO2
S1
dioxane
200 190 180 170 160 150 140 130 120 110 100 90 80 70 60 50 40 30 20 10 0
f1 (ppm)

1H (400.15 MHz, CDCl3)

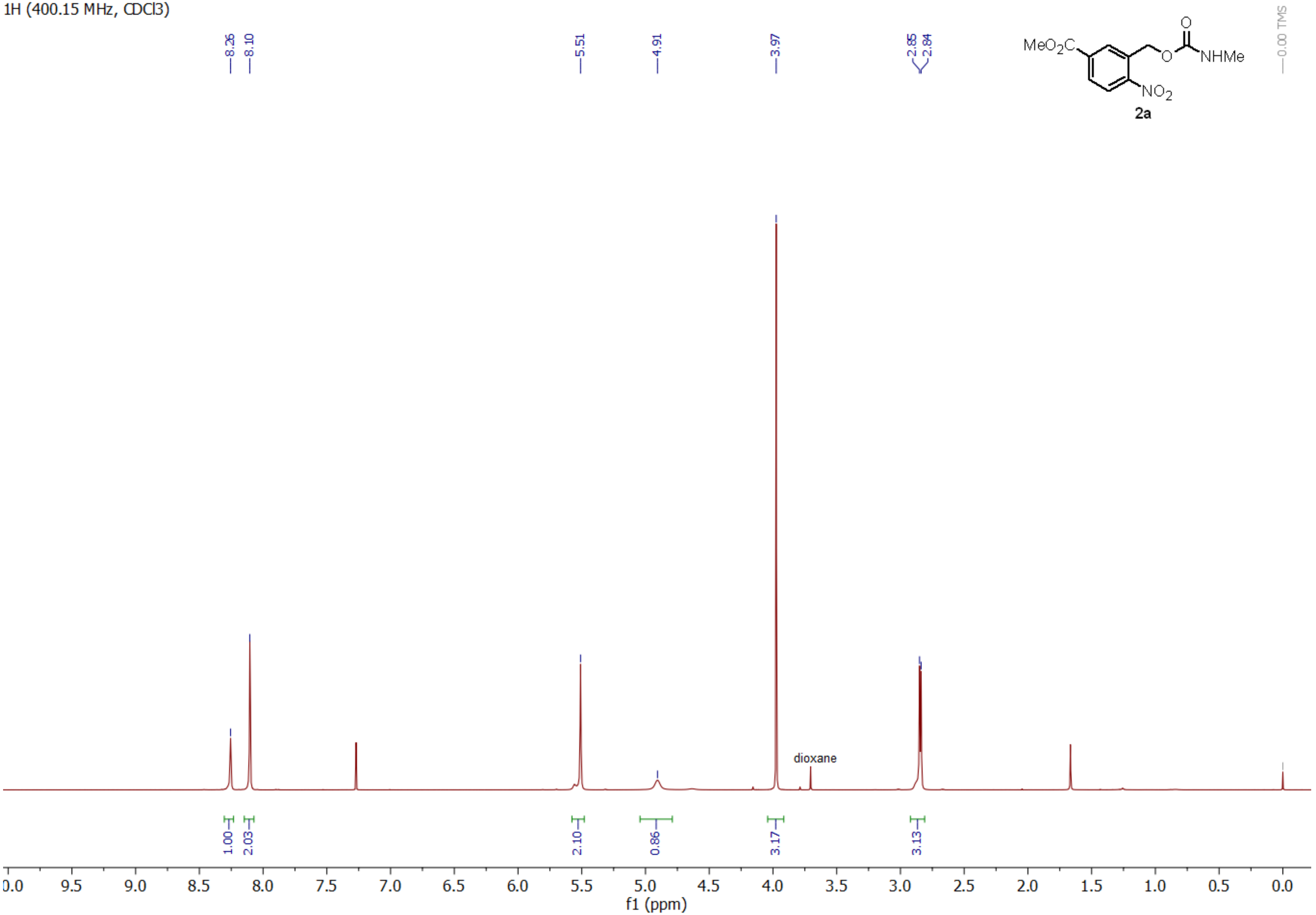

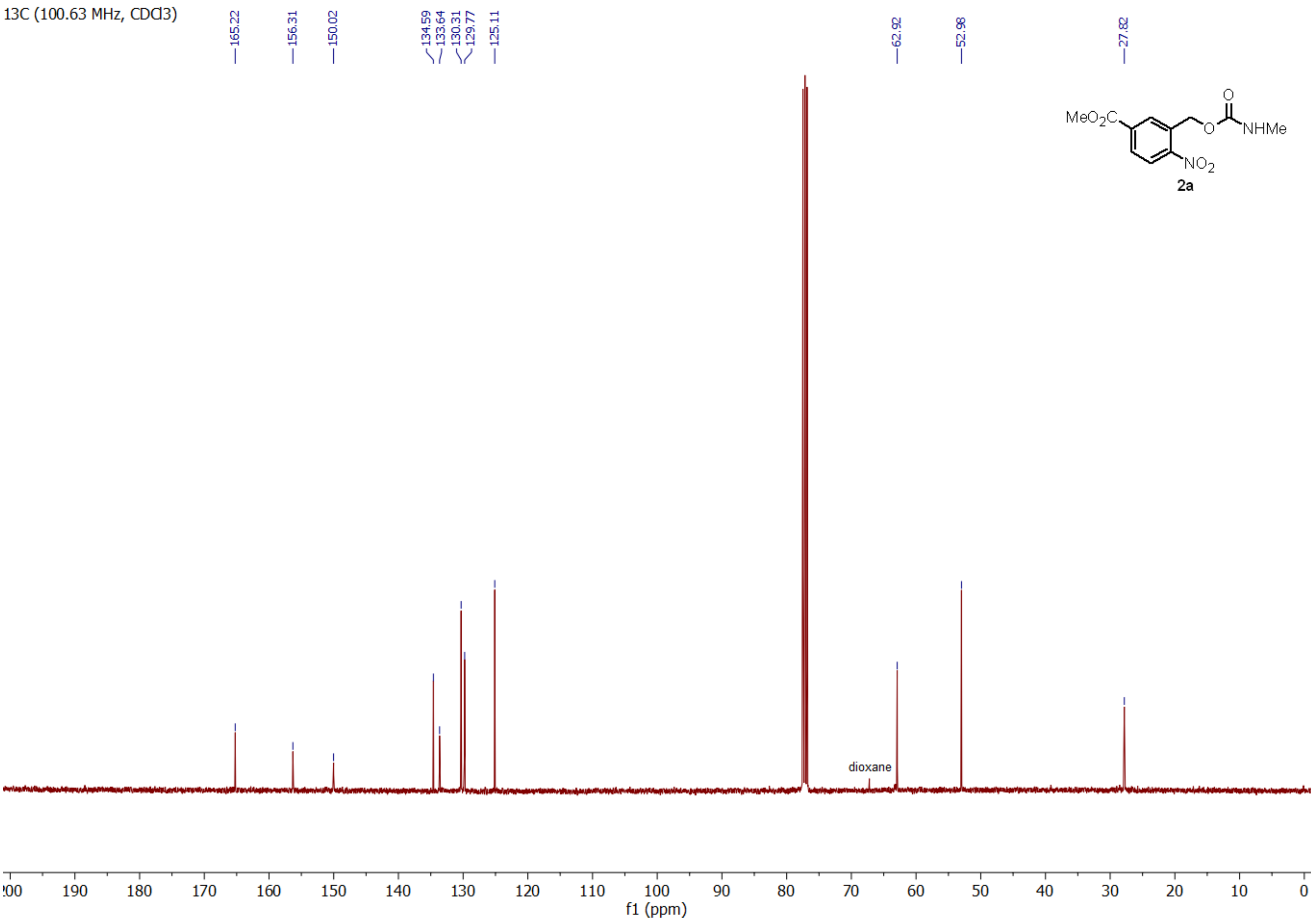

13C (100.63 MHz, CDCl3)
165.22
156.31
150.02
134.59
133.64
130.31
129.77
125.11
62.92
52.98
27.82
MeO2C
O
O
NHMe
NO2
2a
dioxane
200
190
180
170
160
150
140
130
120
110
100
90
80
70
60
50
40
30
20
10
0
f1 (ppm)

1H (400.15 MHz, DMSO)

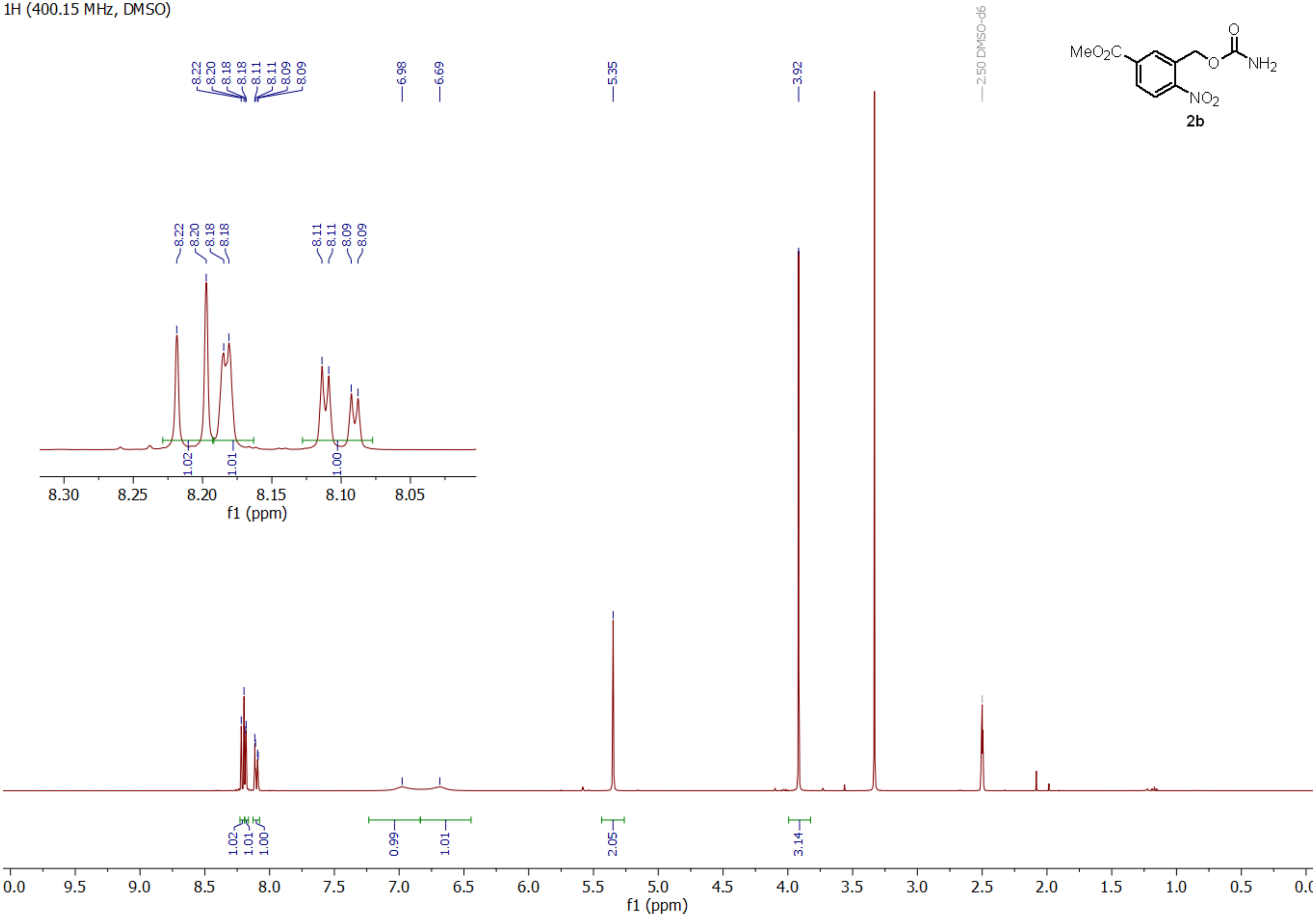

2b

13C (100.63 MHz, DMSO)

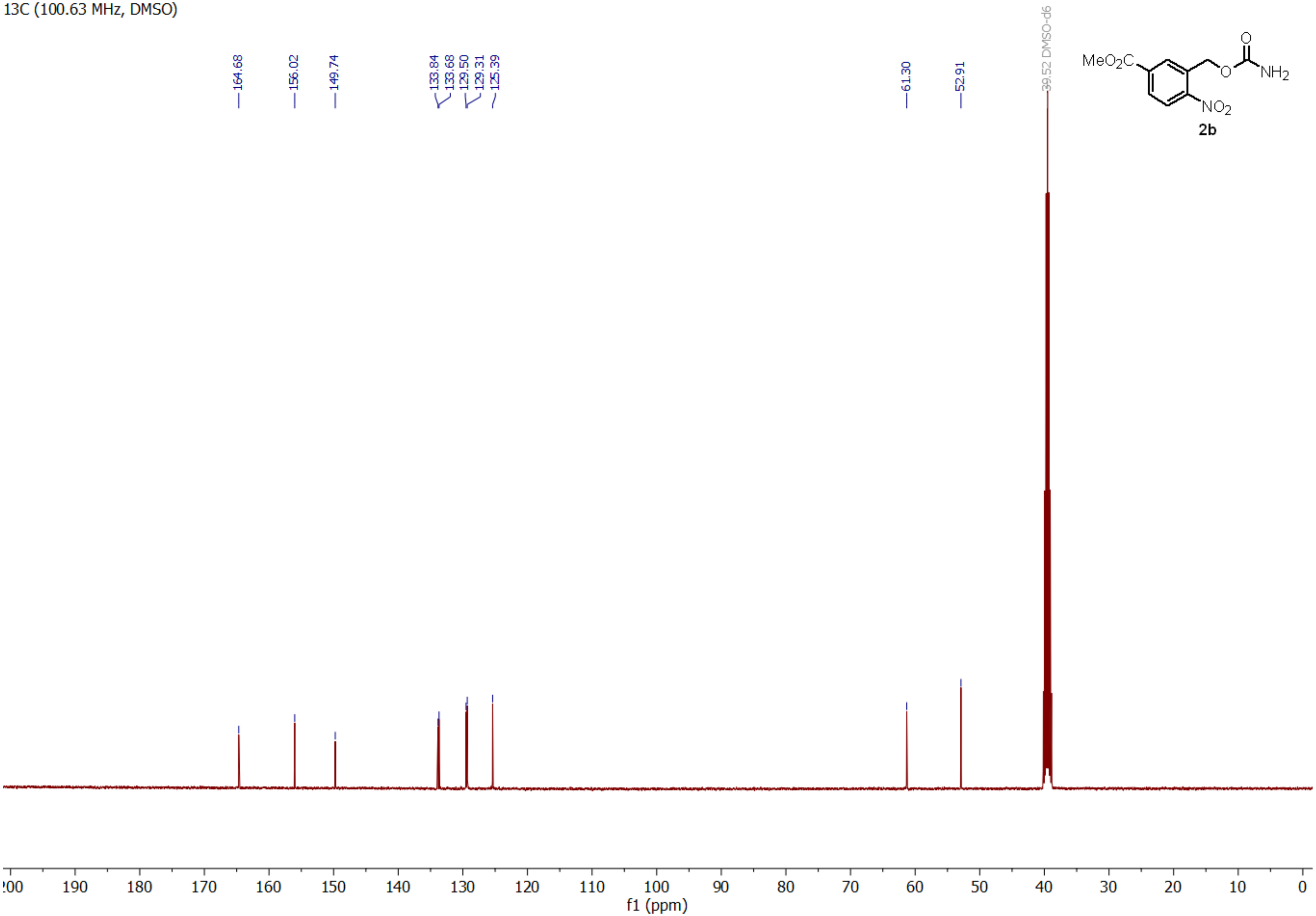

2b

1H (400.15 MHz, CDCl3)

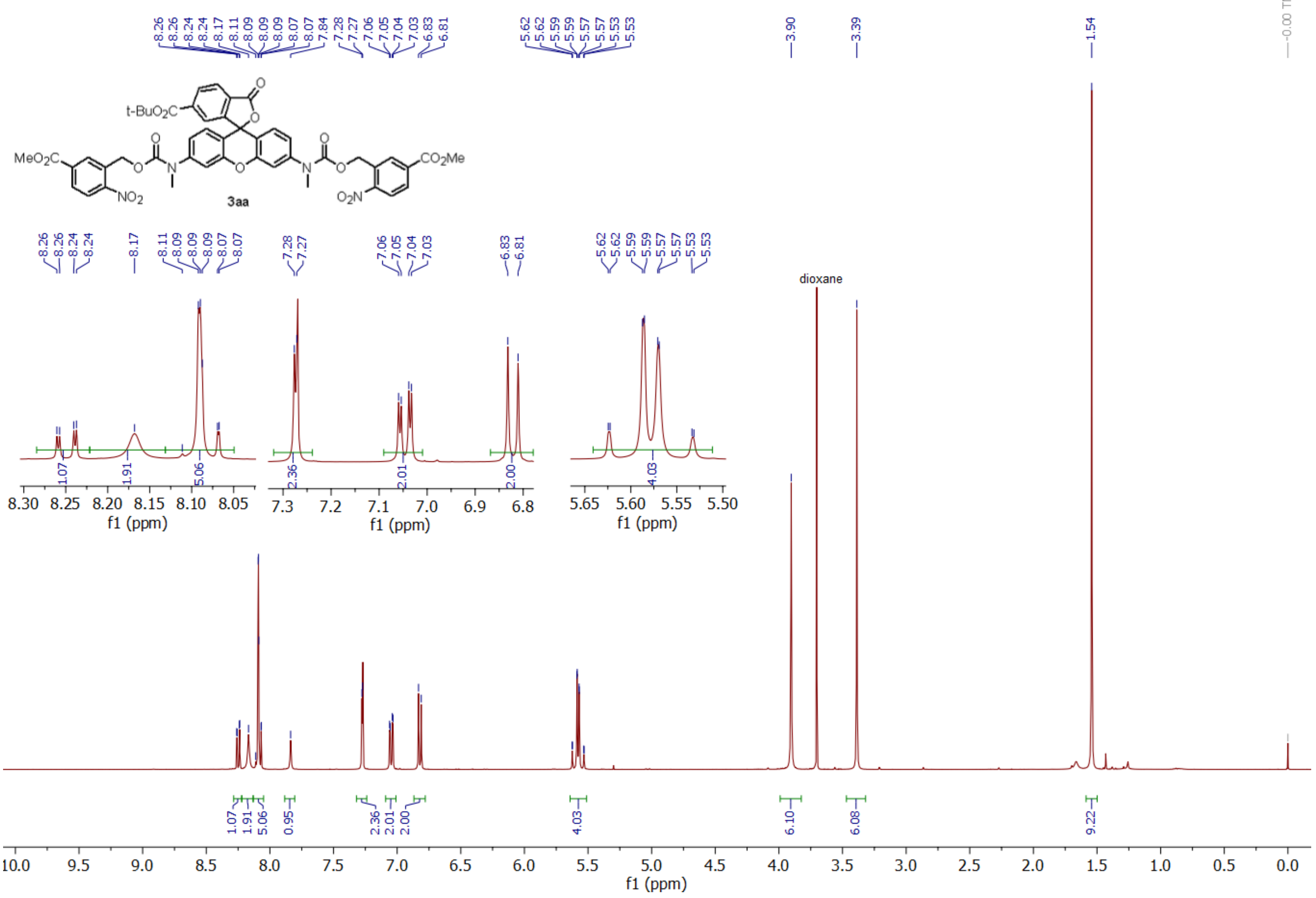

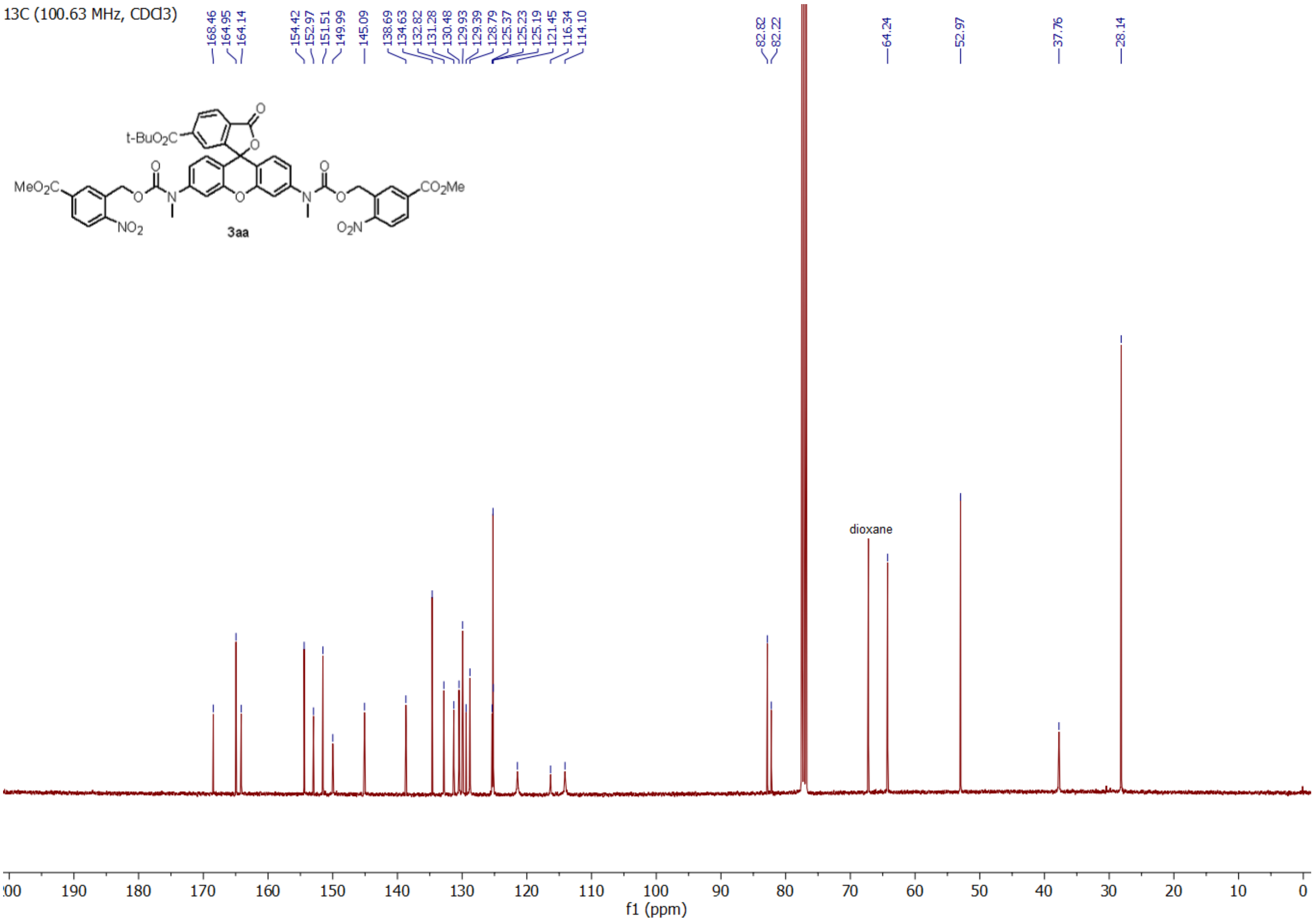
13C (100.63 MHz, CDCl3)
168.46
164.95
164.14
154.42
152.97
151.51
149.99
145.09
138.69
134.63
132.82
131.28
130.48
129.93
129.39
128.79
125.37
125.23
125.19
121.45
116.34
114.10
82.82
82.22
64.24
52.97
37.76
28.14
t-BuO2C
MeO2C
CO2Me
NO2
O2N
3aa
dioxane
f1 (ppm)

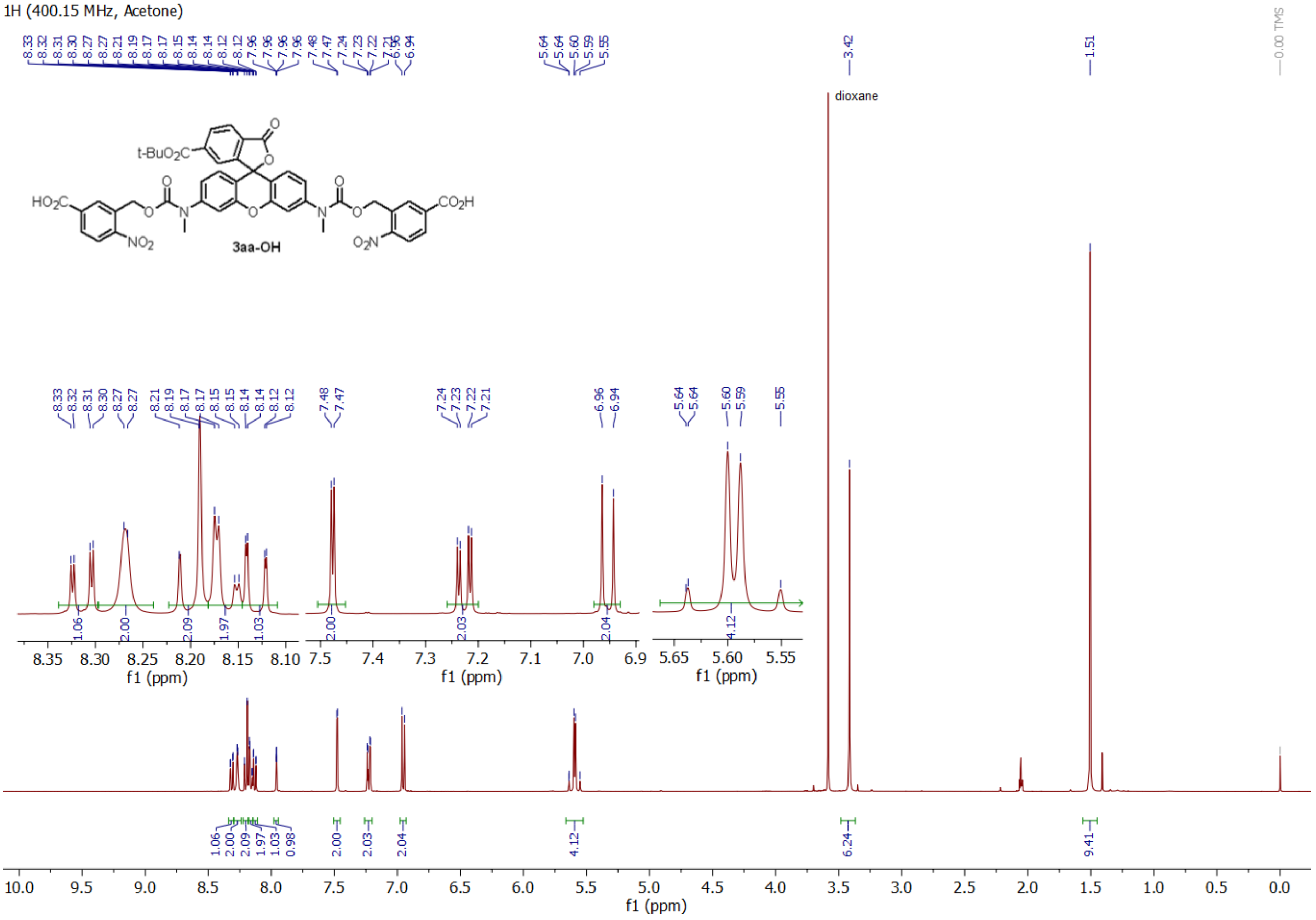
1H (400.15 MHz, Acetone)
t-BuO2C
HO2C
CO2H
NO2
O2N
3aa-OH
dioxane
TMS
f1 (ppm)

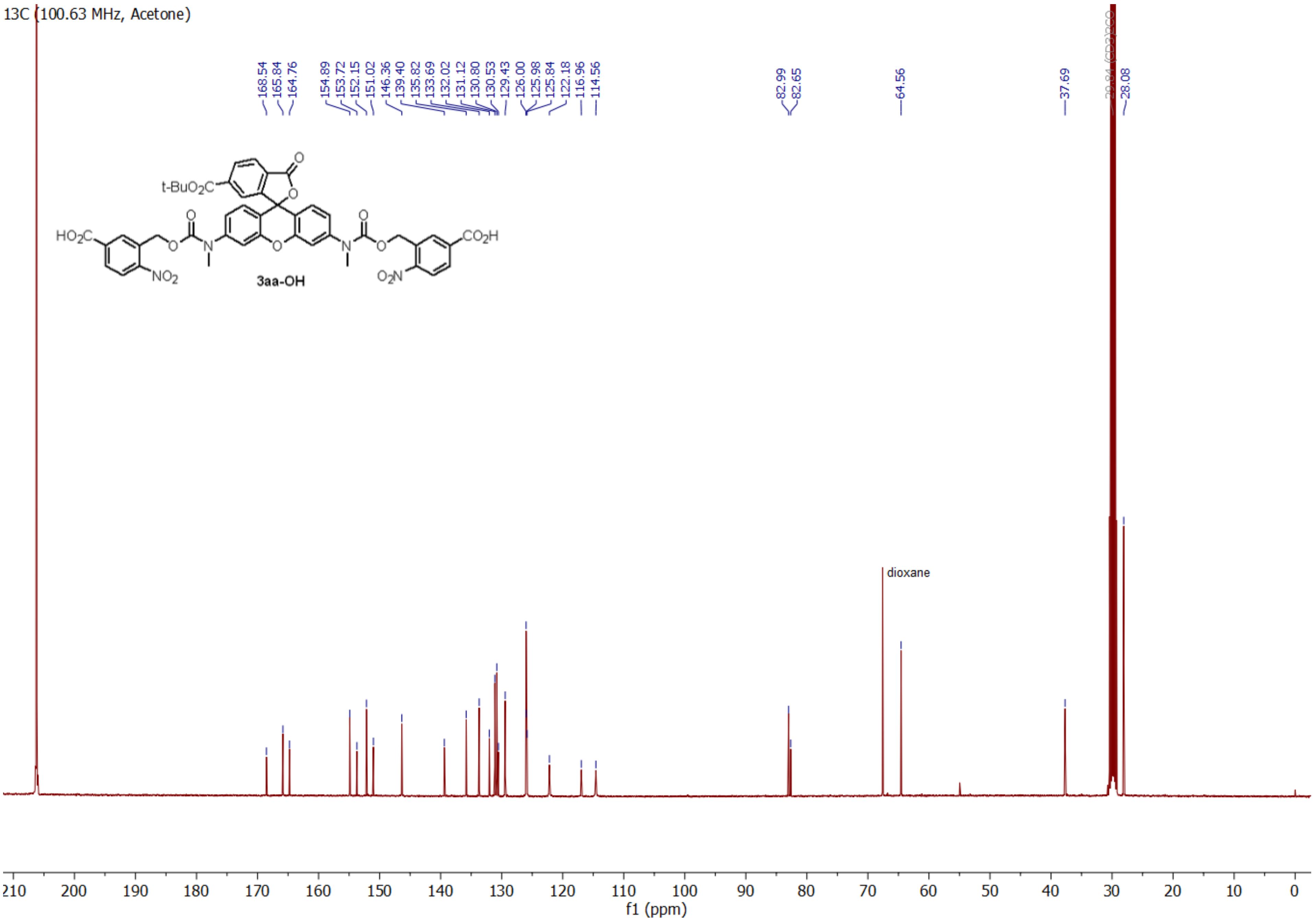

13C (100.63 MHz, Acetone)
168.54
165.84
164.76
154.89
153.72
152.15
151.02
146.36
139.40
135.82
133.69
132.02
131.12
130.80
130.53
129.43
126.00
125.98
125.84
122.18
116.96
114.56
82.99
82.65
64.56
37.69
29.84 (CD3)2CO
28.08
t-BuO2C
HO2C
CO2H
NO2
O2N
3aa-OH
dioxane
f1 (ppm)

1H (400.15 MHz, DMSO)

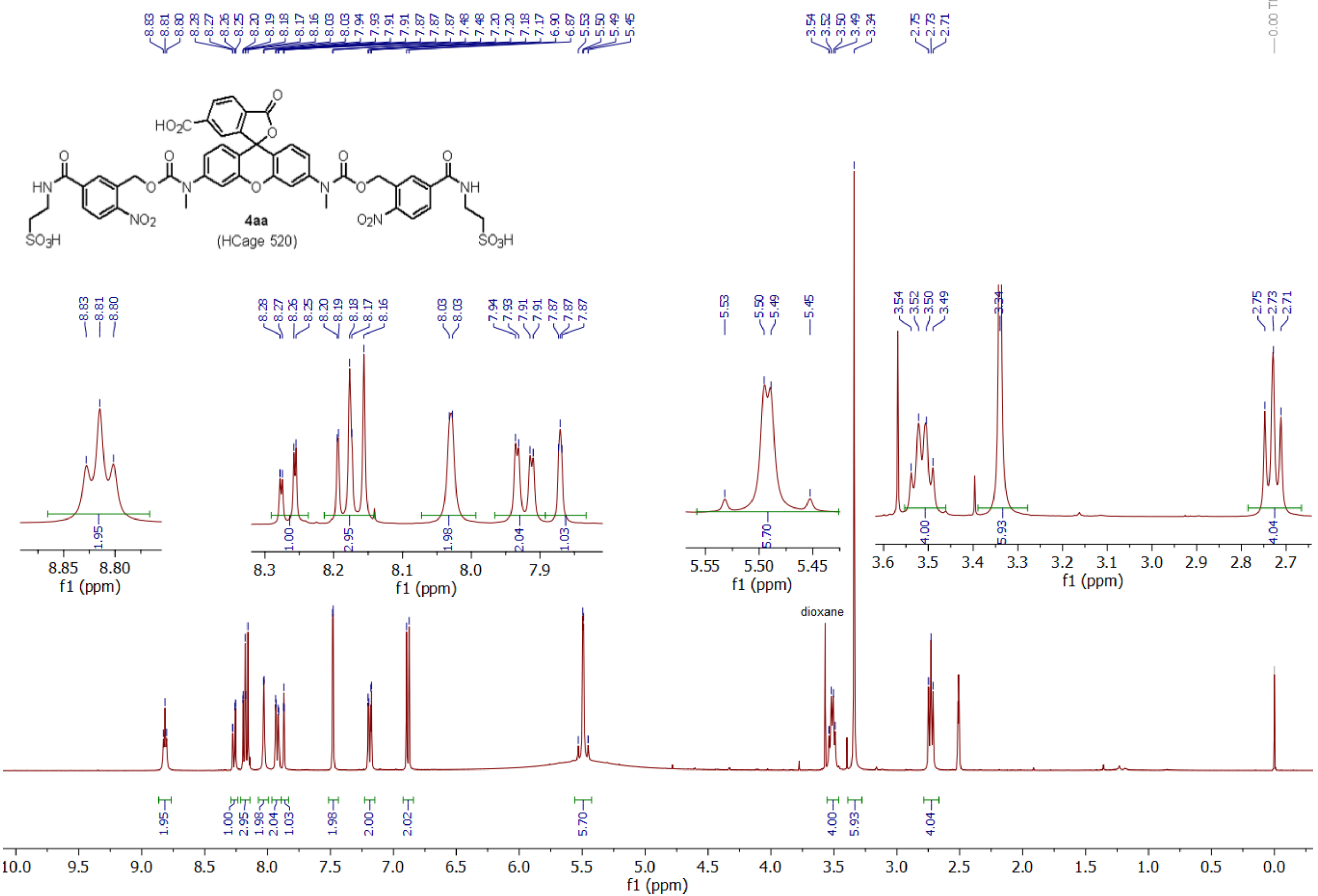

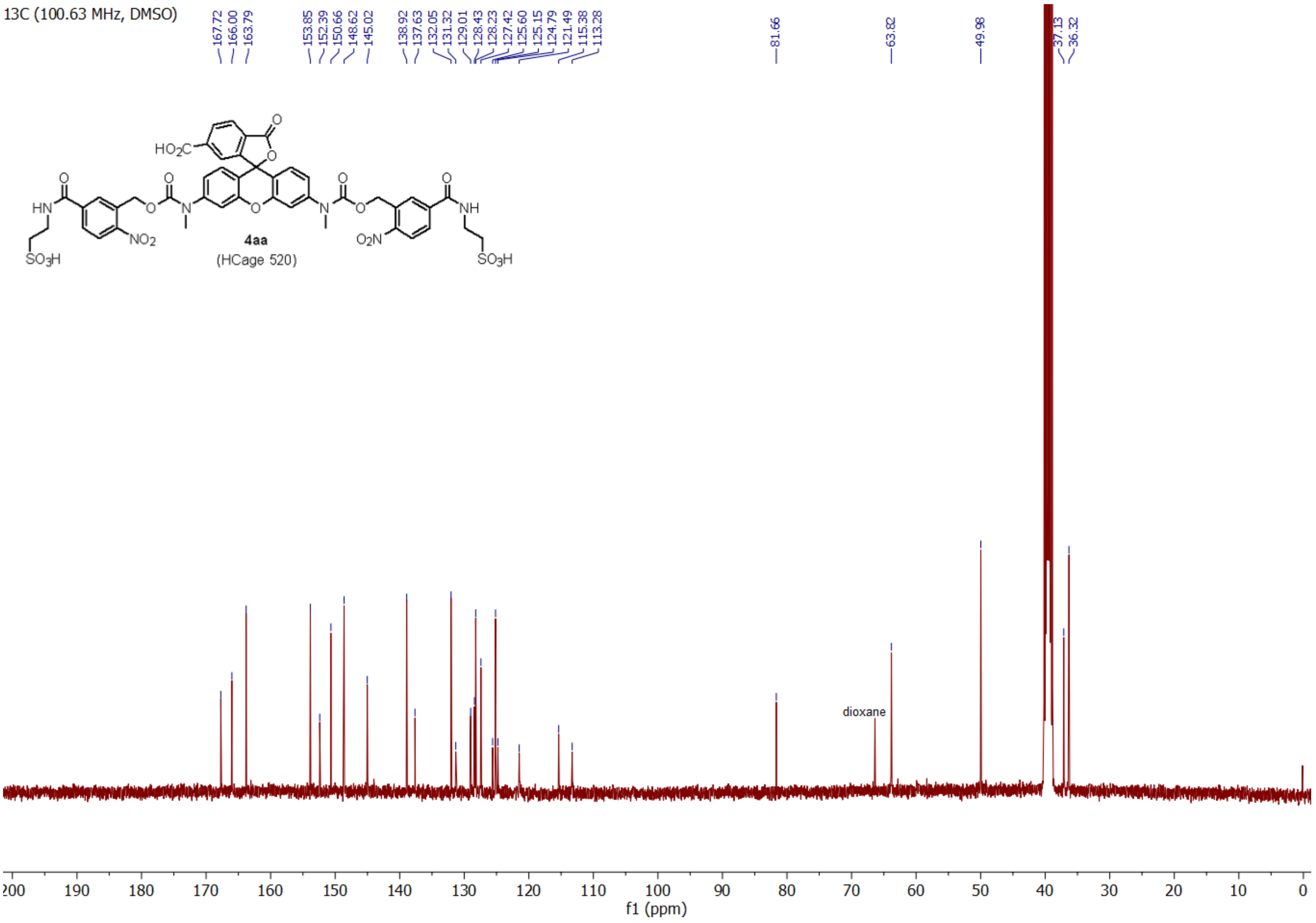
13C (100.63 MHz, DMSO)
167.72
166.00
163.79
153.85
152.39
150.66
148.62
145.02
138.92
137.63
132.05
131.32
129.01
128.43
128.23
127.42
125.60
125.15
124.79
121.49
115.38
113.28
81.66
63.82
49.98
37.13
36.32
HO2C
O
HN
NO2
SO3H
N
NH
O2N
4aa
(HCage 520)
dioxane
200
190
180
170
160
150
140
130
120
110
100
90
80
70
60
50
40
30
20
10
0
f1 (ppm)

1H (400.15 MHz, CDCl3)

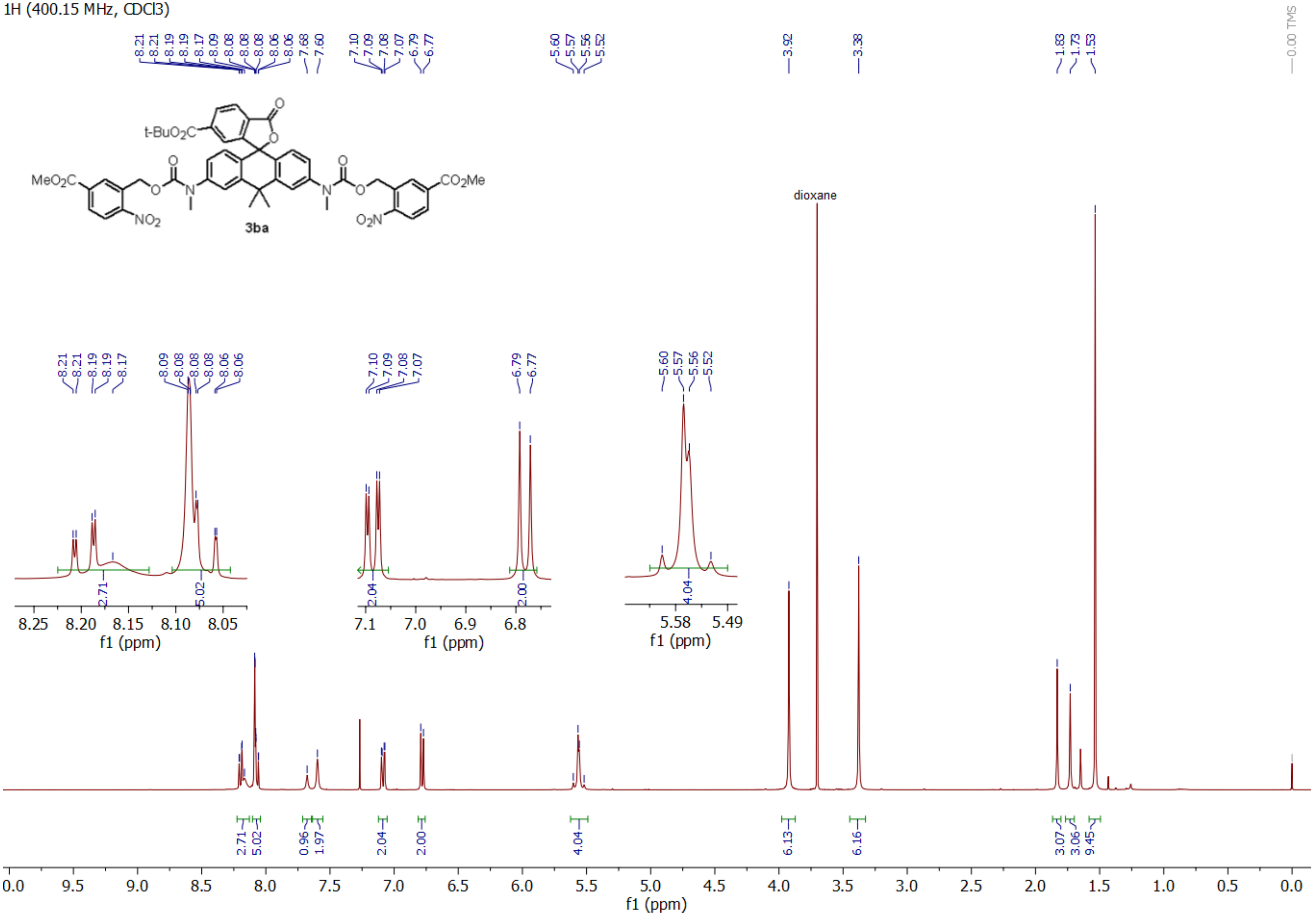

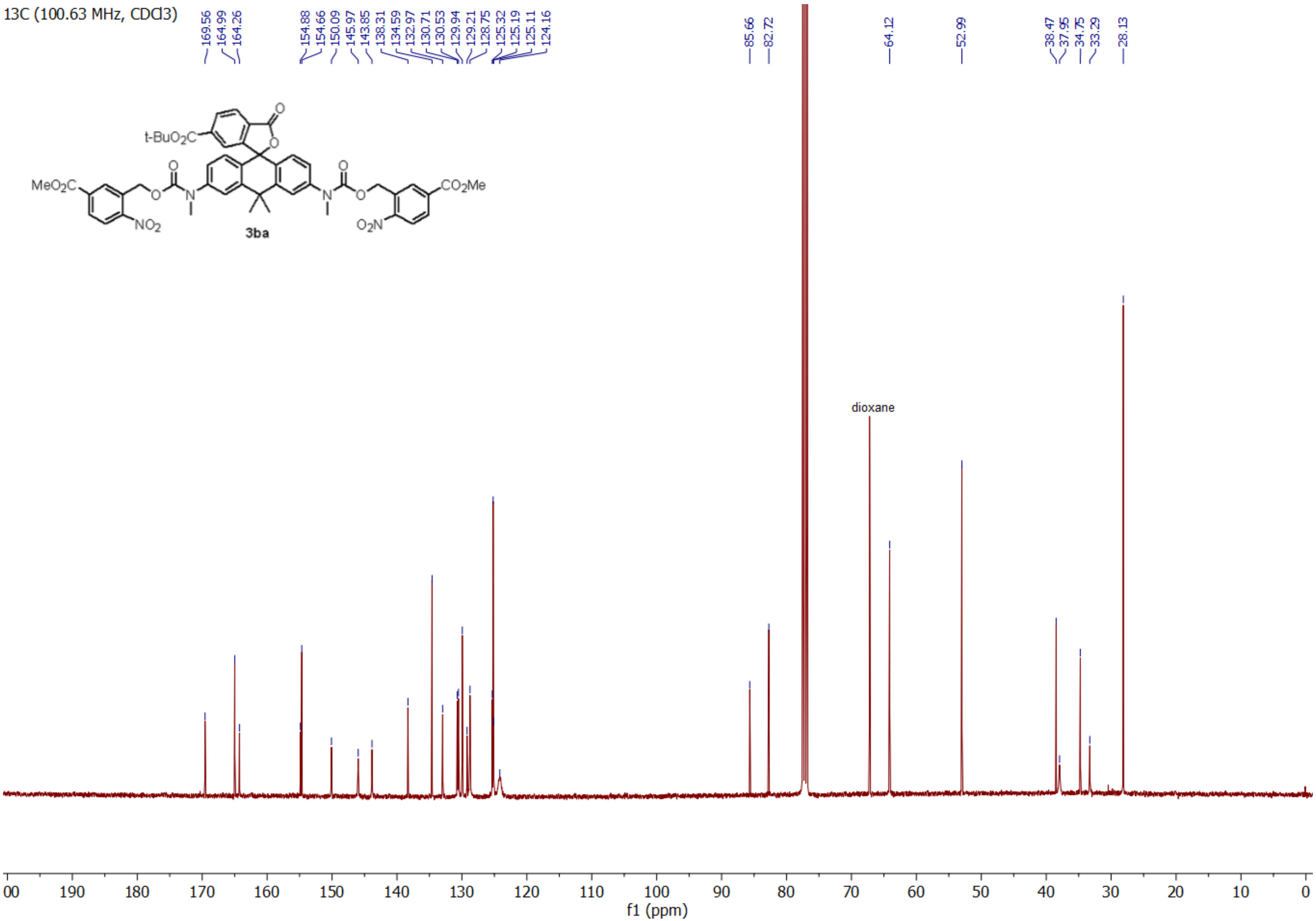
13C (100.63 MHz, CDCl3)
169.56
164.99
164.26
154.88
154.66
150.09
145.97
143.85
138.31
134.59
132.97
130.71
130.53
129.94
129.21
128.75
125.32
125.19
125.11
124.16
85.66
82.72
64.12
52.99
38.47
37.95
34.75
33.29
28.13
t-BuO2C
MeO2C
CO2Me
NO2
O2N
3ba
dioxane
f1 (ppm)

1H (400.15 MHz, Acetone)

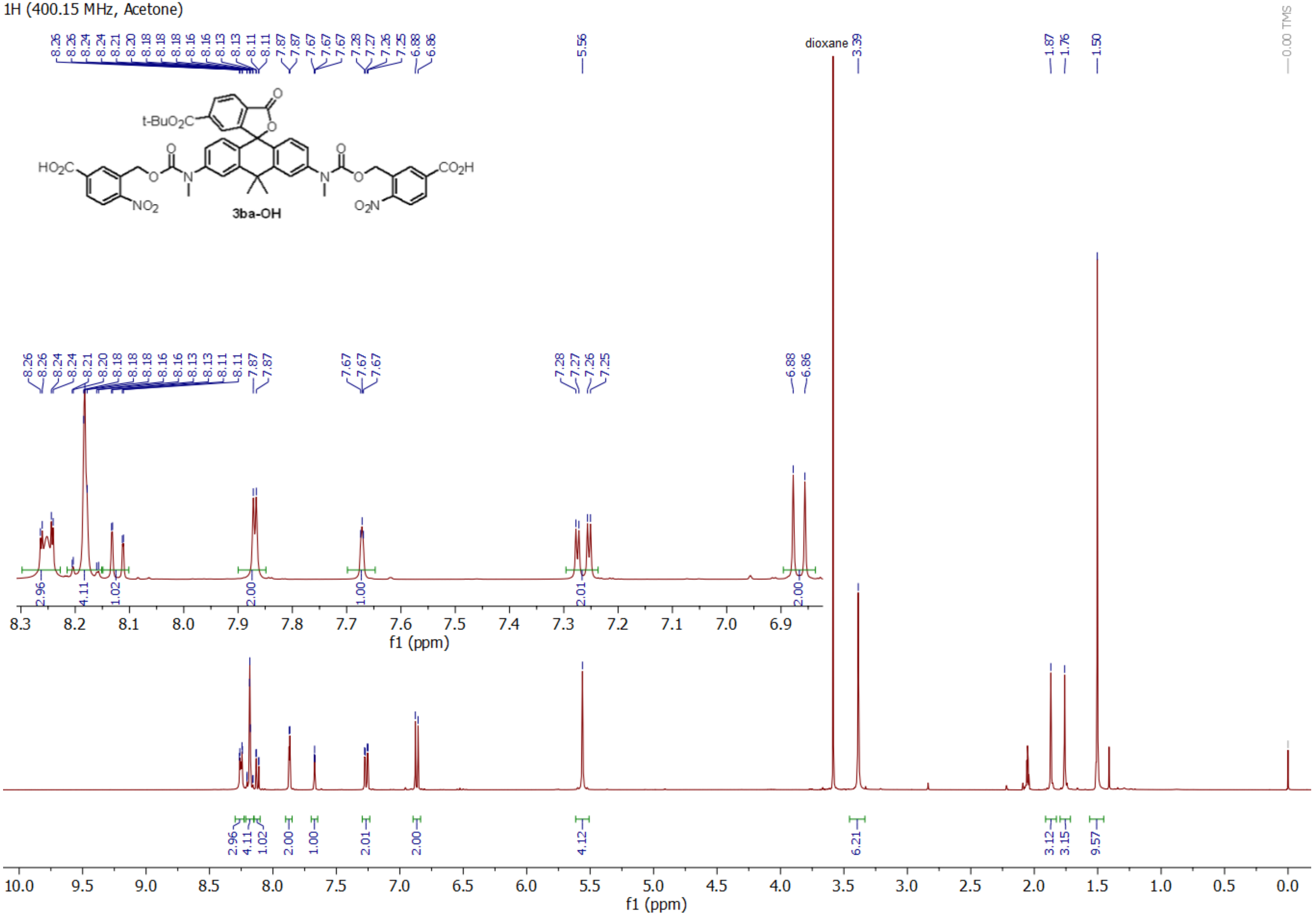

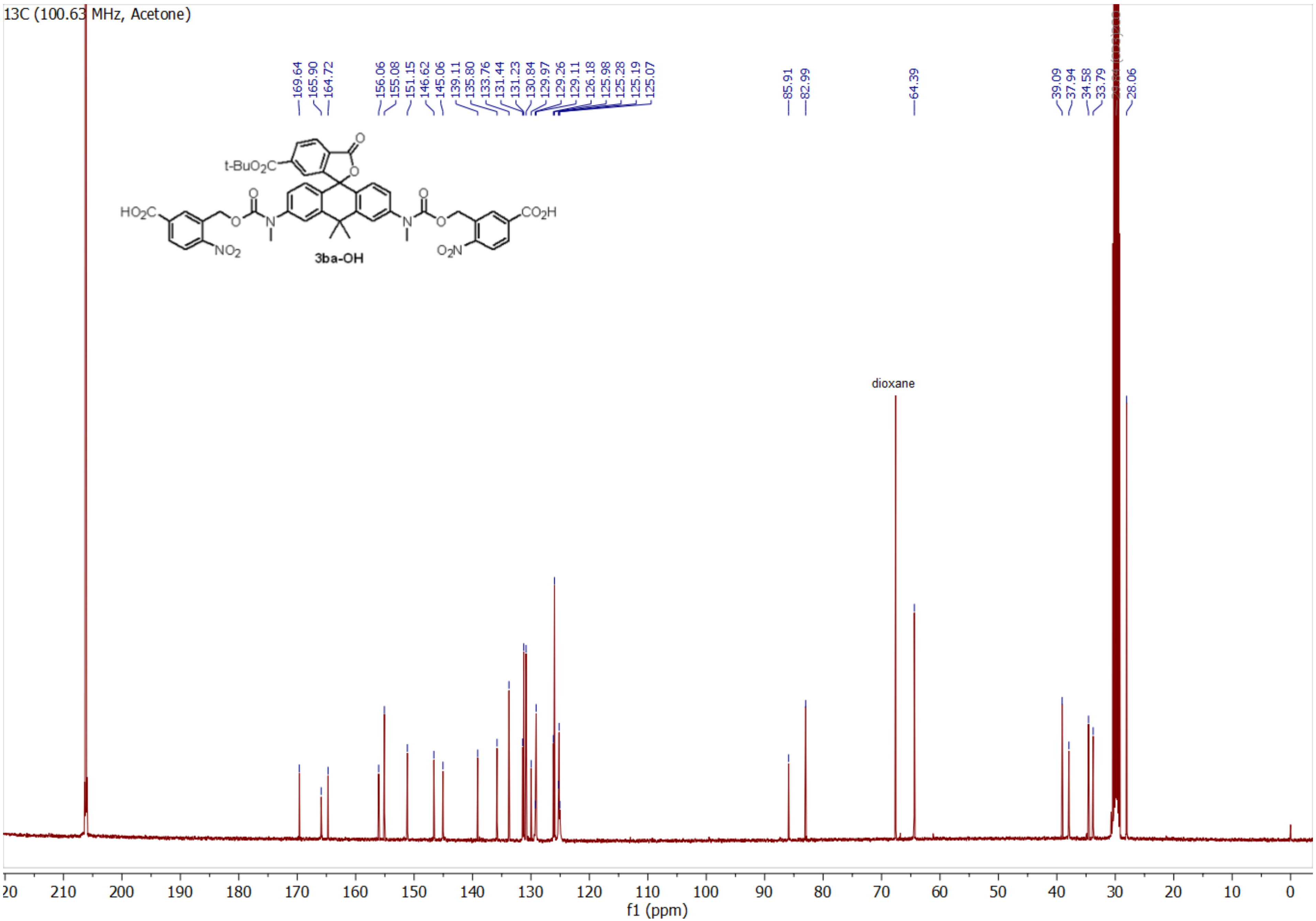
13C (100.63 MHz, Acetone)
169.64
165.90
164.72
156.06
155.08
151.15
146.62
145.06
139.11
135.80
133.76
131.44
131.23
130.84
129.97
129.26
129.11
126.18
125.98
125.28
125.19
125.07
85.91
82.99
64.39
39.09
37.94
34.58
33.79
28.06
t-BuO2C
HO2C
CO2H
NO2
O2N
3ba-OH
dioxane
f1 (ppm)

1H (400.15 MHz, MeOD)

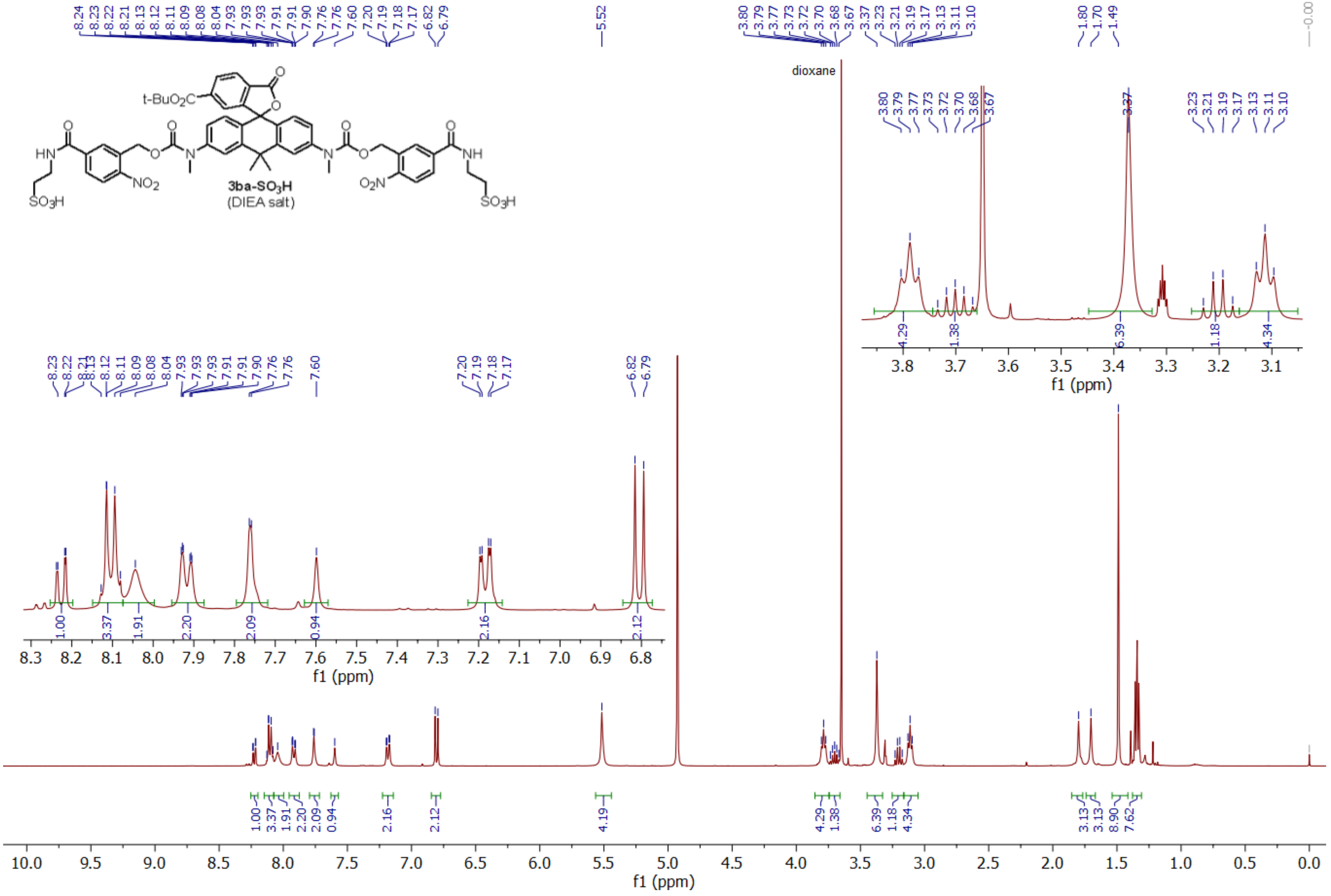

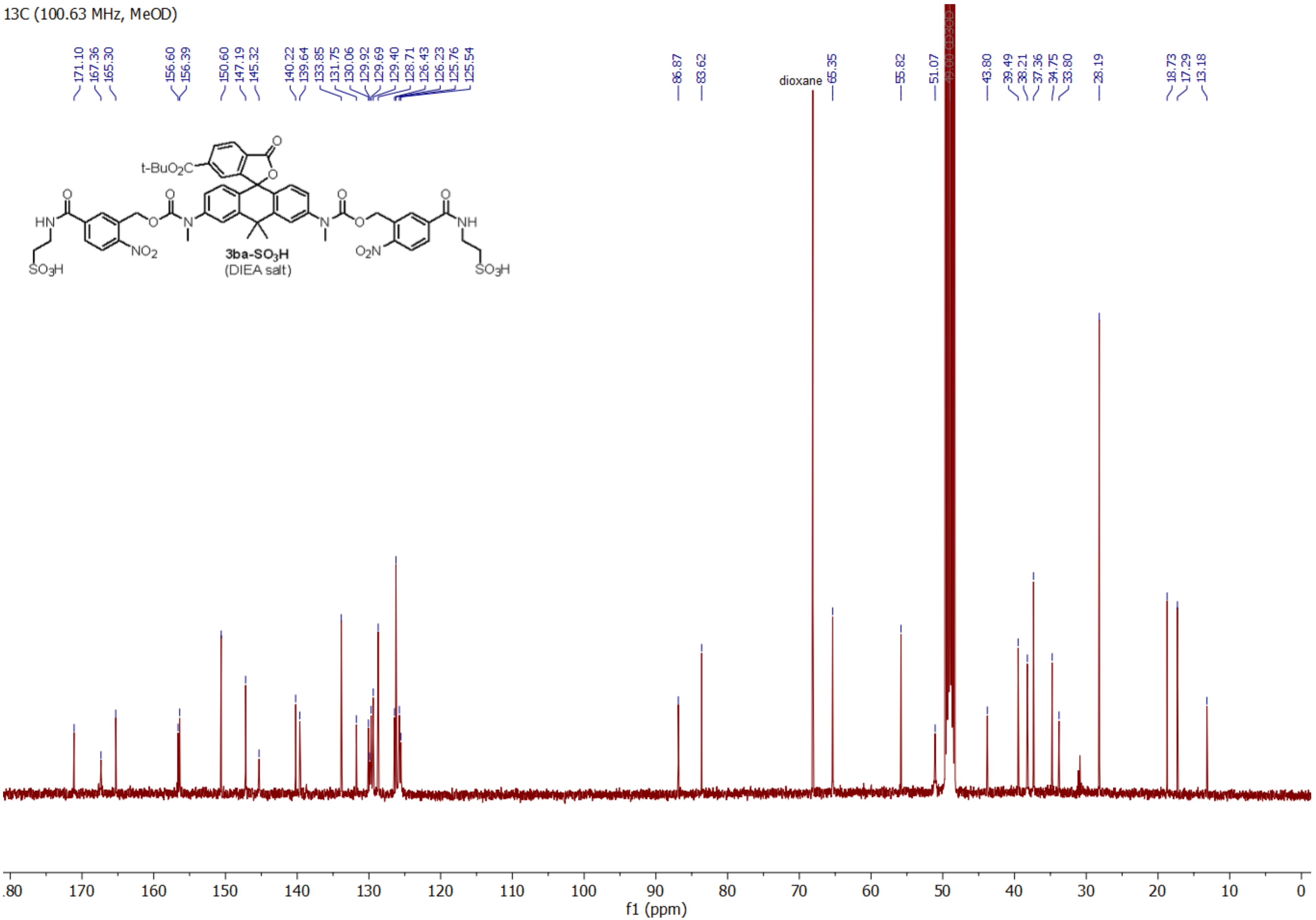
13C (100.63 MHz, MeOD)
171.10
167.36
165.30
156.60
156.39
150.60
147.19
145.32
140.22
139.64
133.85
131.75
130.06
129.92
129.69
129.40
128.71
126.43
126.23
125.76
125.54
86.87
83.62
dioxane
65.35
55.82
51.07
49.00 CD3OD
43.80
39.49
38.21
37.36
34.75
33.80
28.19
18.73
17.29
13.18
t-BuO2C
HN
SO3H
NO2
3ba-SO3H
(DIEA salt)
O2N
NH
SO3H
f1 (ppm)

1H (400.15 MHz, DMSO)

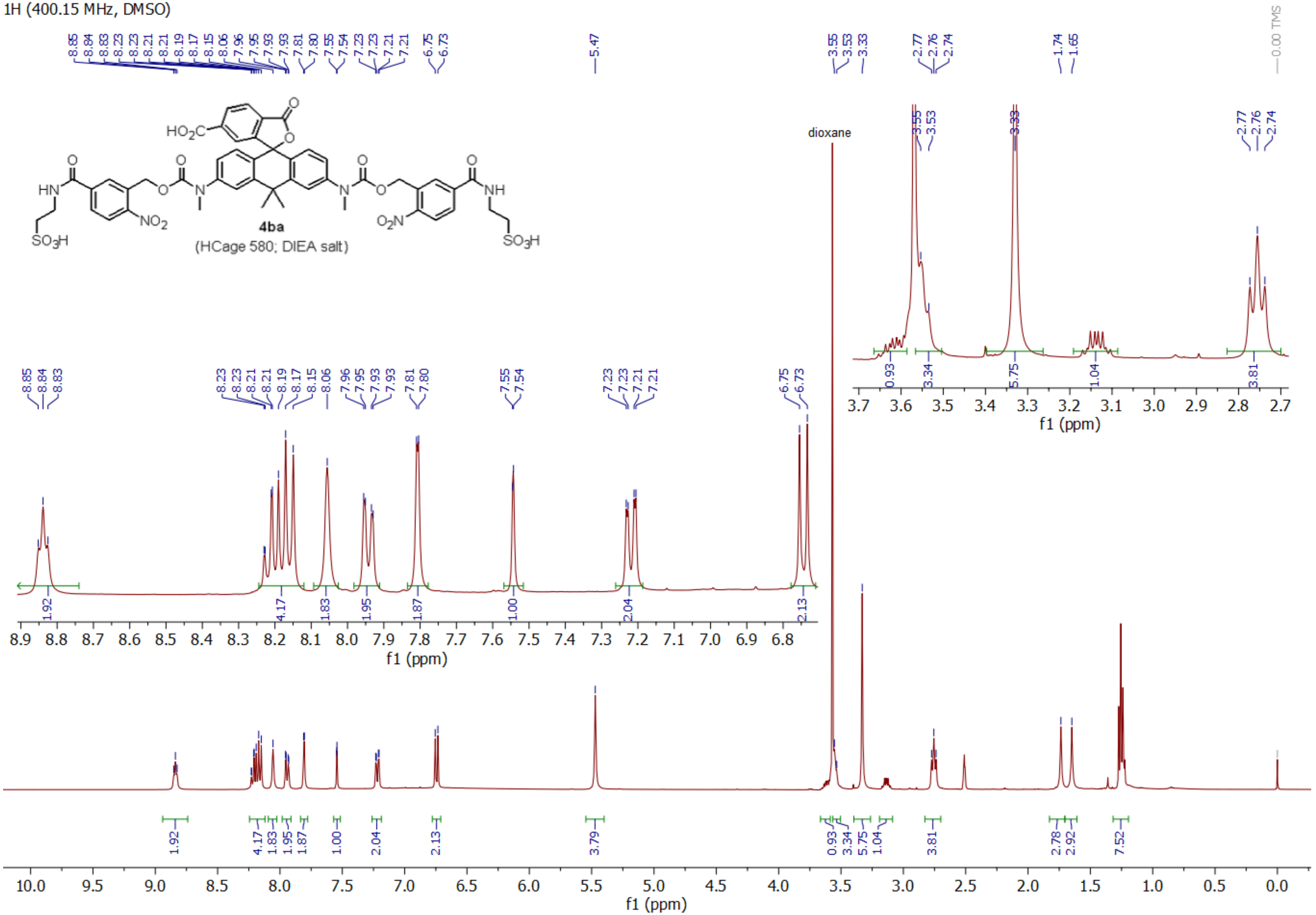

13C (100.63 MHz, DMSO)

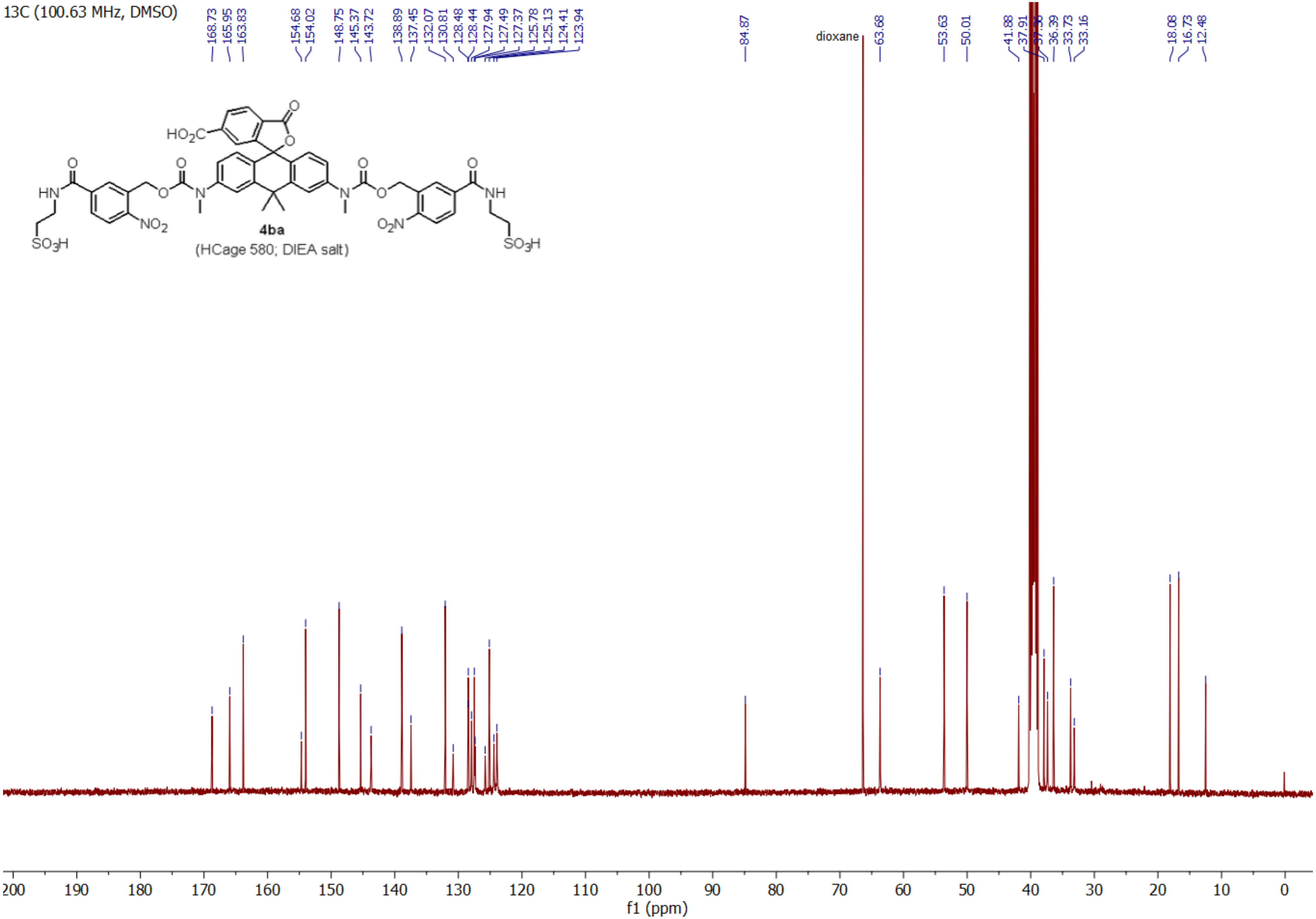

4ba
(HCage 580; DIEA salt)

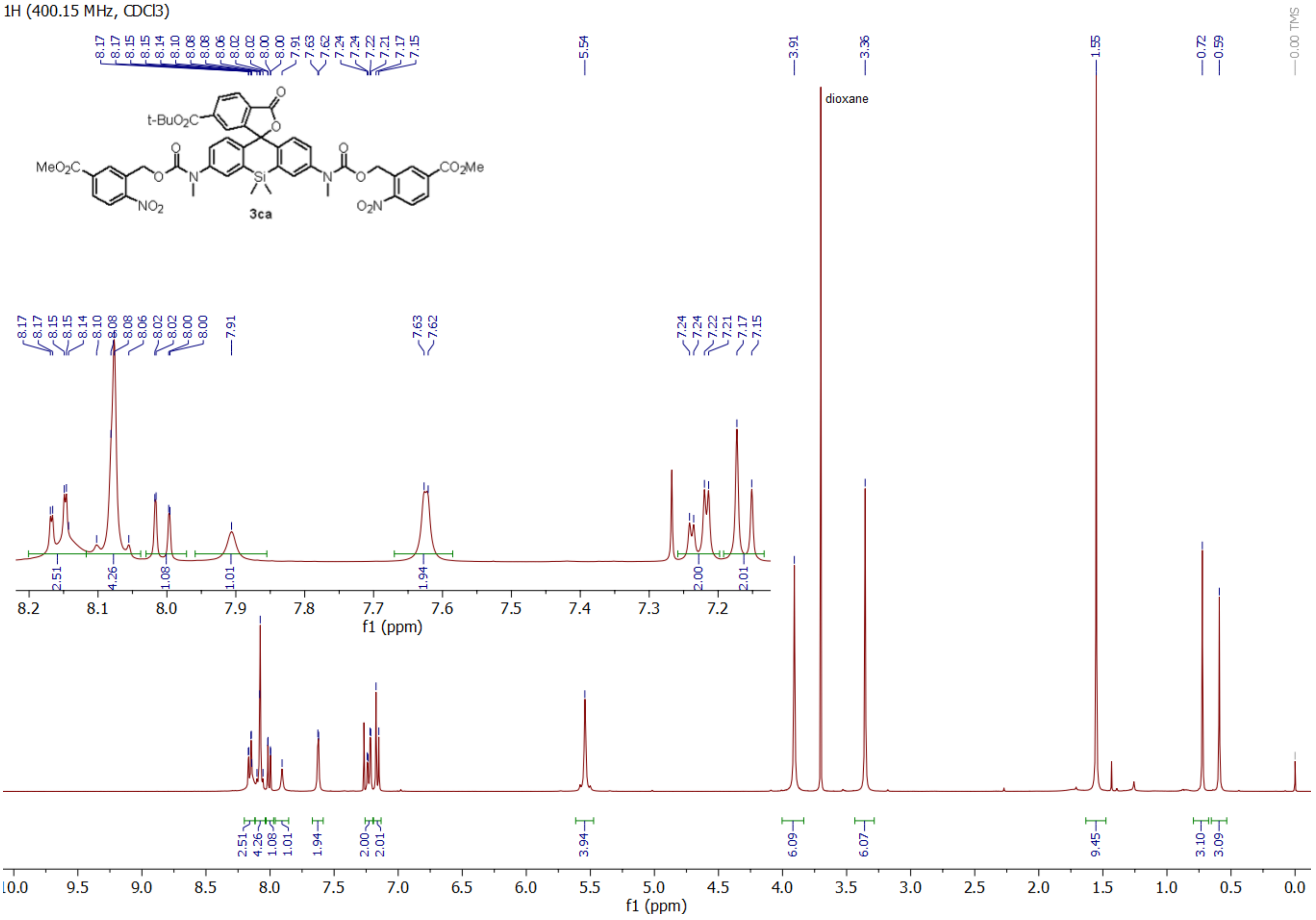
1H (400.15 MHz, CDCl3)
8.17 8.17 8.15 8.15 8.14 8.10 8.08 8.08 8.06 8.02 8.02 8.00 8.00 7.91 7.63 7.62 7.24 7.24 7.22 7.21 7.17 7.15
5.54
3.91
3.36
1.55
0.72
0.59
0.00 TMS
dioxane
t-BuO2C
MeO2C
CO2Me
NO2
O2N
Si
N
O
3ca
2.51 4.26 1.08 1.01 1.94 2.00 2.01 3.94 6.09 6.07 9.45 3.10 3.09
f1 (ppm)

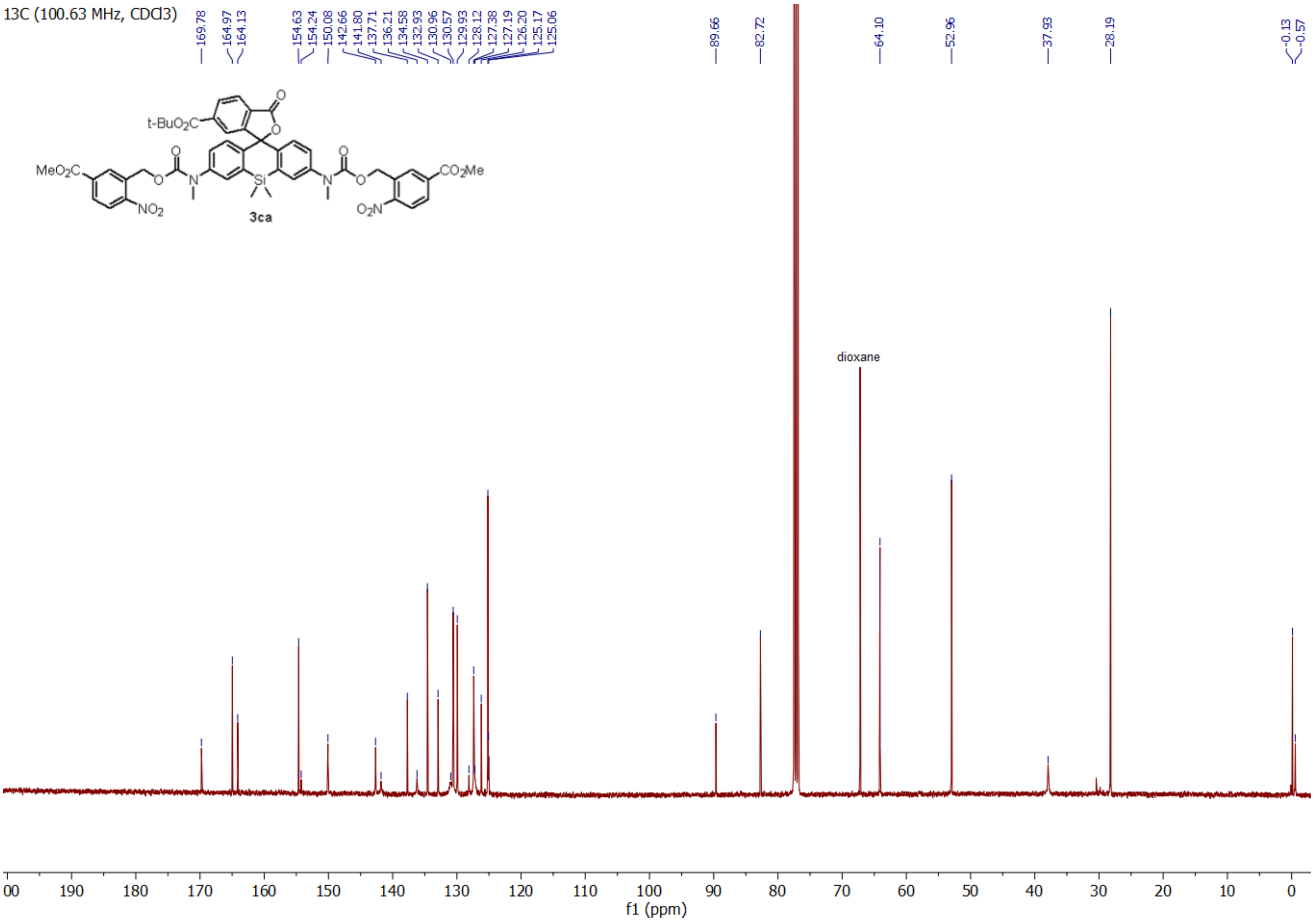
13C (100.63 MHz, CDCl3)
169.78
164.97
164.13
154.63
154.24
150.08
142.66
141.80
137.71
136.21
134.58
132.93
130.96
130.57
129.93
128.12
127.38
127.19
126.20
125.17
125.06
89.66
82.72
64.10
52.96
37.93
28.19
-0.13
-0.57
t-BuO2C
MeO2C
CO2Me
NO2
O2N
Si
3ca
dioxane
f1 (ppm)

1H (400.15 MHz, Acetone)

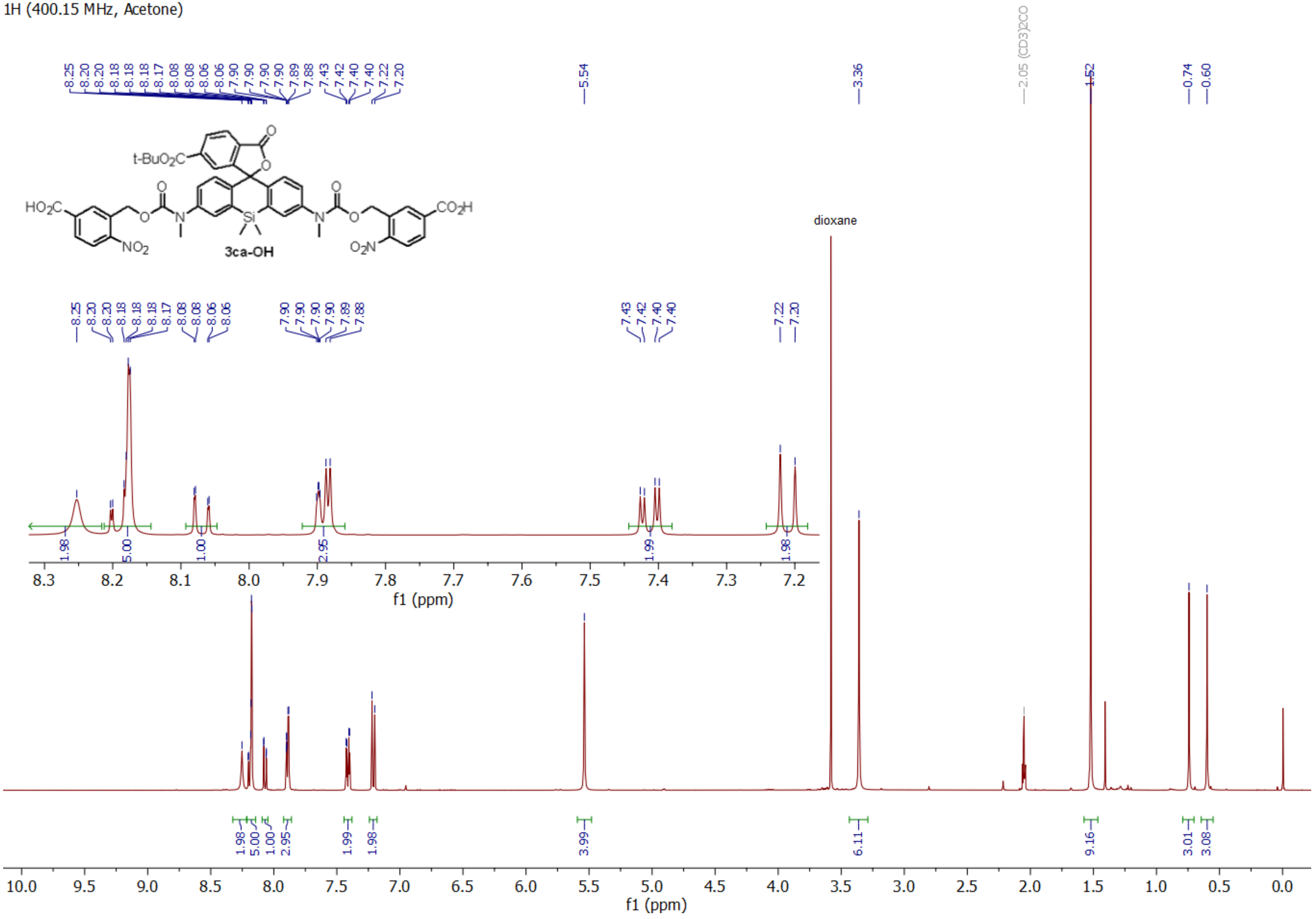

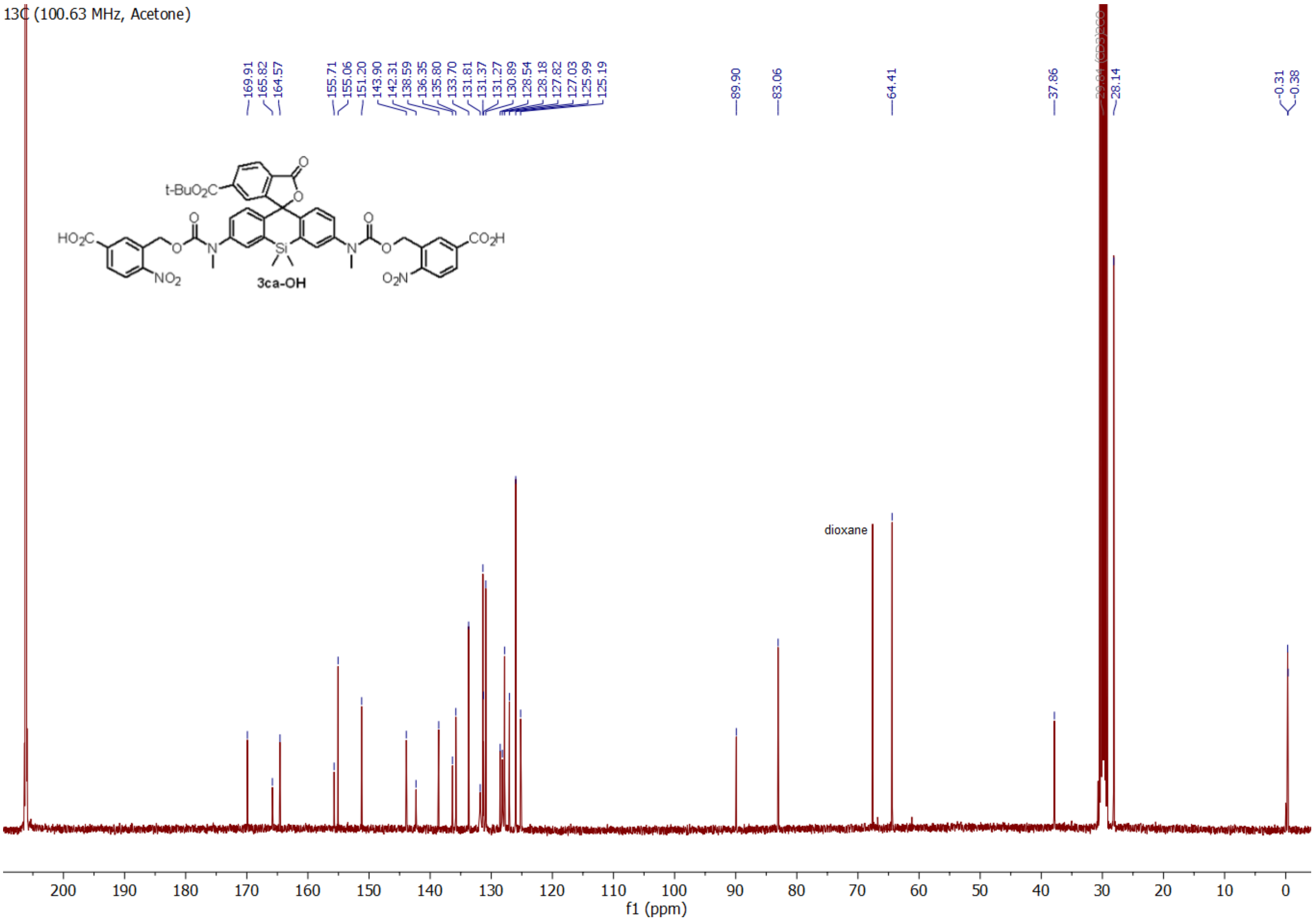

13C (100.63 MHz, Acetone)
169.91
165.82
164.57
155.71
155.06
151.20
143.90
142.31
138.59
136.35
135.80
133.70
131.81
131.37
131.27
130.89
128.54
128.18
127.82
127.03
125.99
125.19
89.90
83.06
64.41
37.86
29.84 (CD3)2CO
28.14
-0.31
-0.38
t-BuO2C
HO2C
CO2H
NO2
O2N
Si
3ca-OH
dioxane
f1 (ppm)

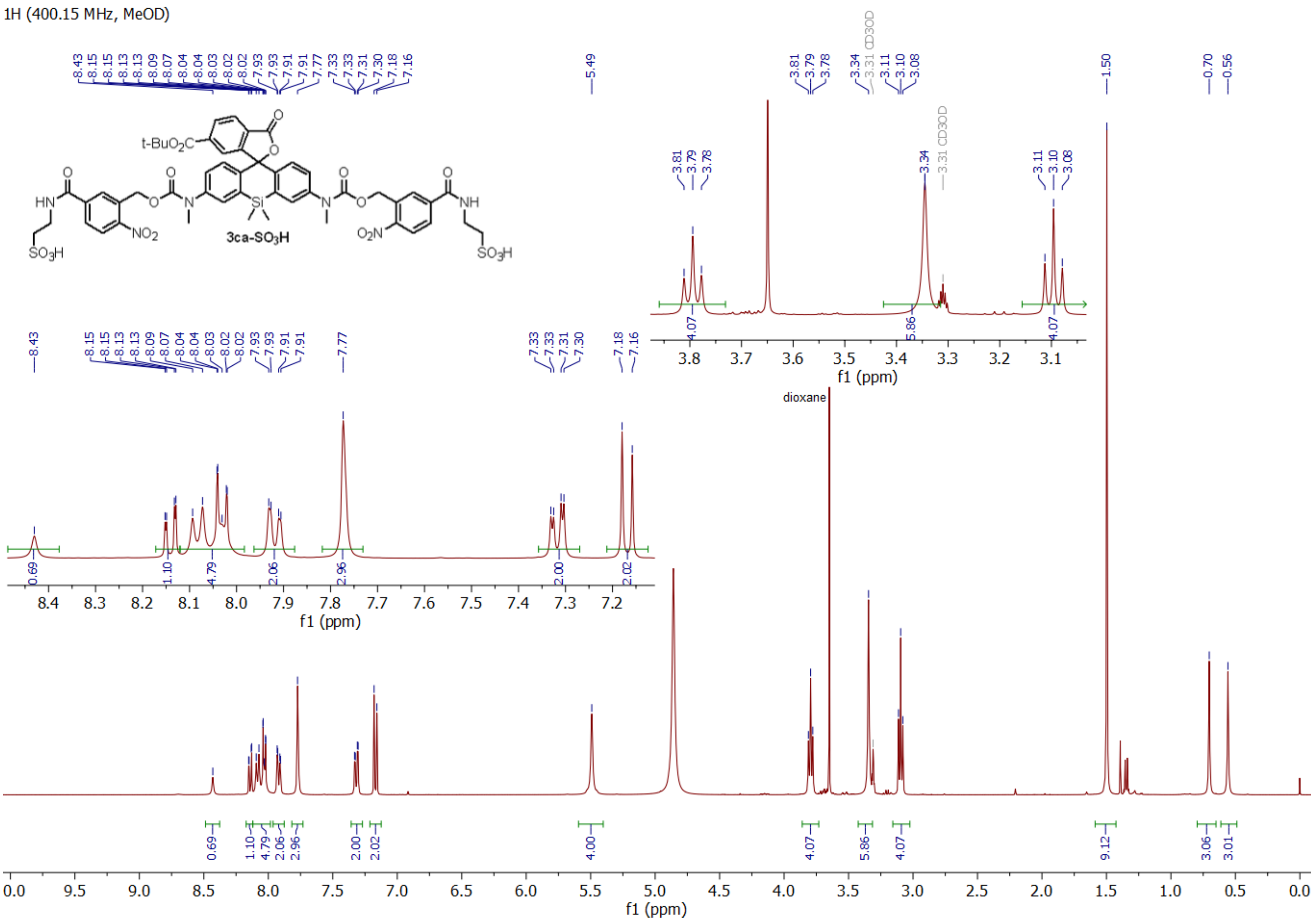

1H (400.15 MHz, MeOD)
3ca-SO3H
t-BuO2C
HN
NH
SO3H
NO2
O2N
Si
dioxane
CD3OD
f1 (ppm)

13C (100.63 MHz, MeOD)

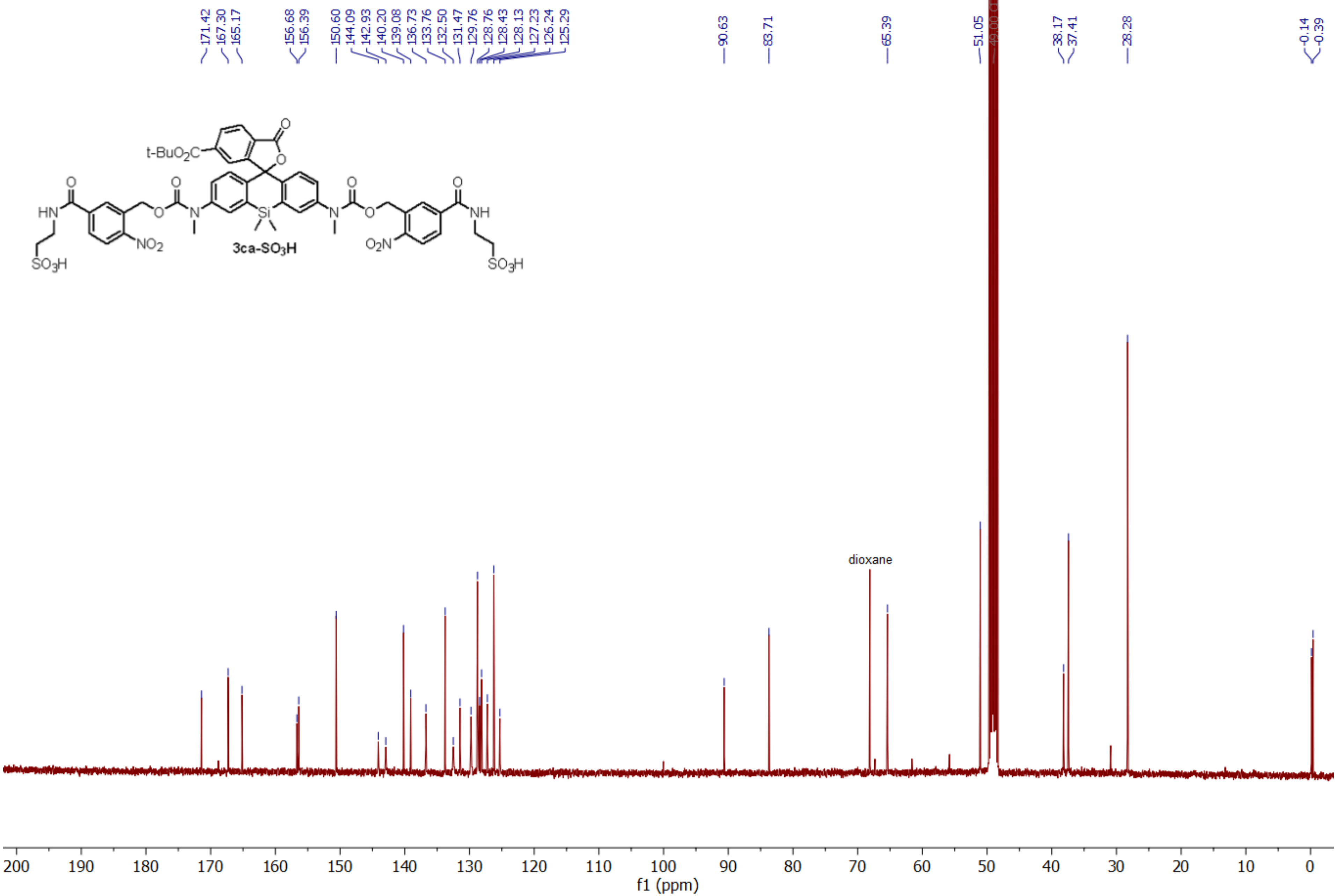

1H (400.15 MHz, DMSO)

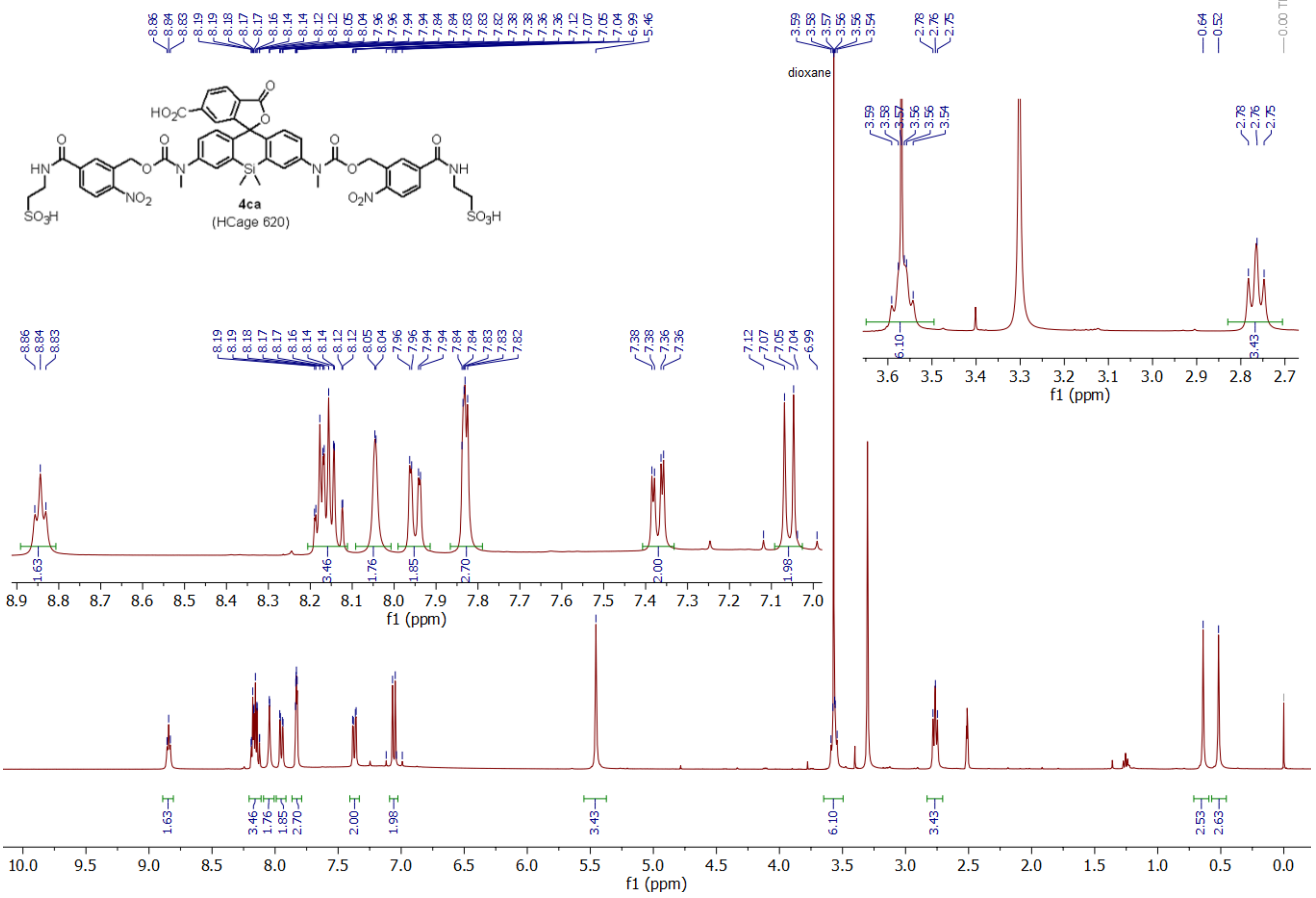

13C (100.63 MHz, DMSO)

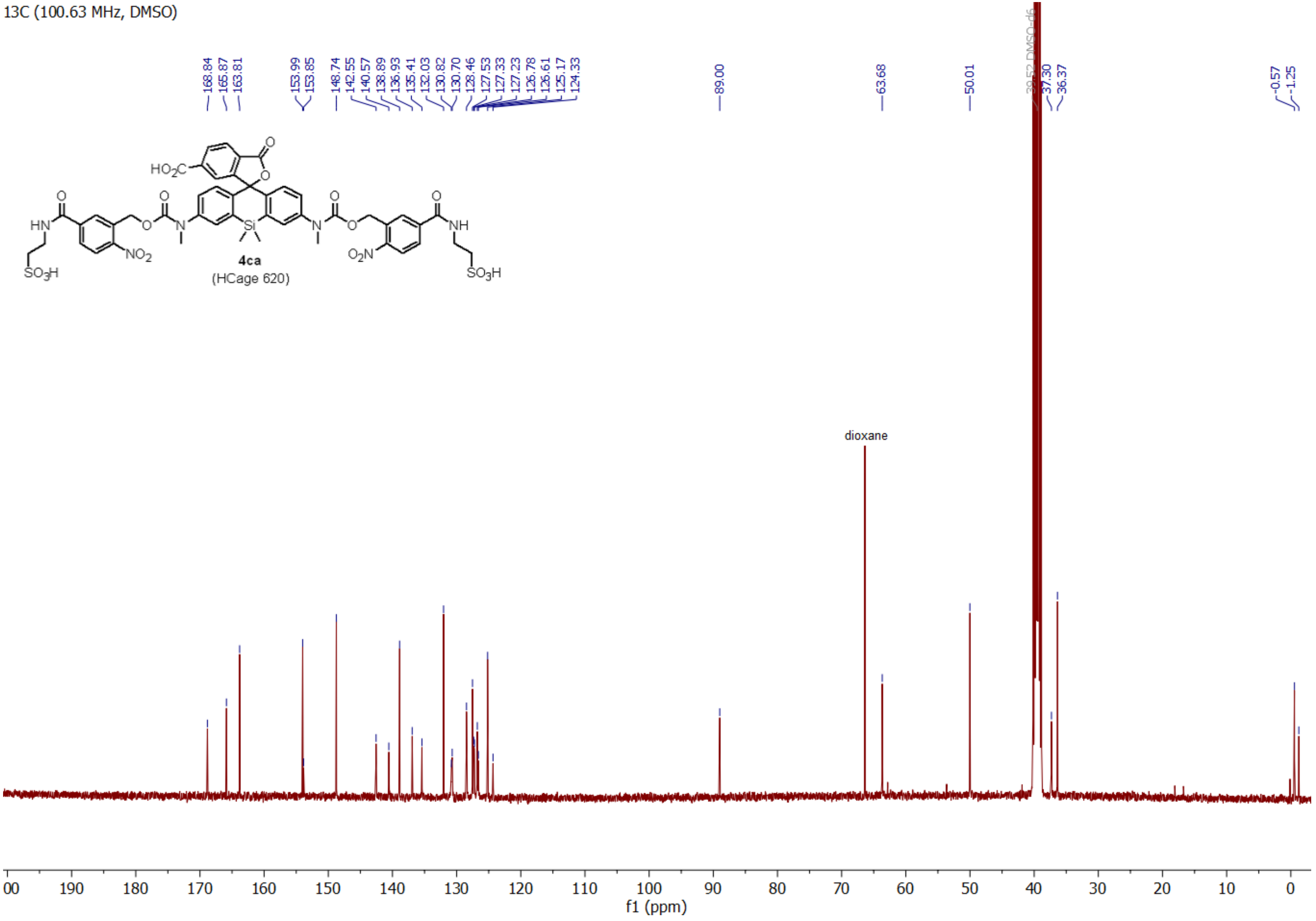

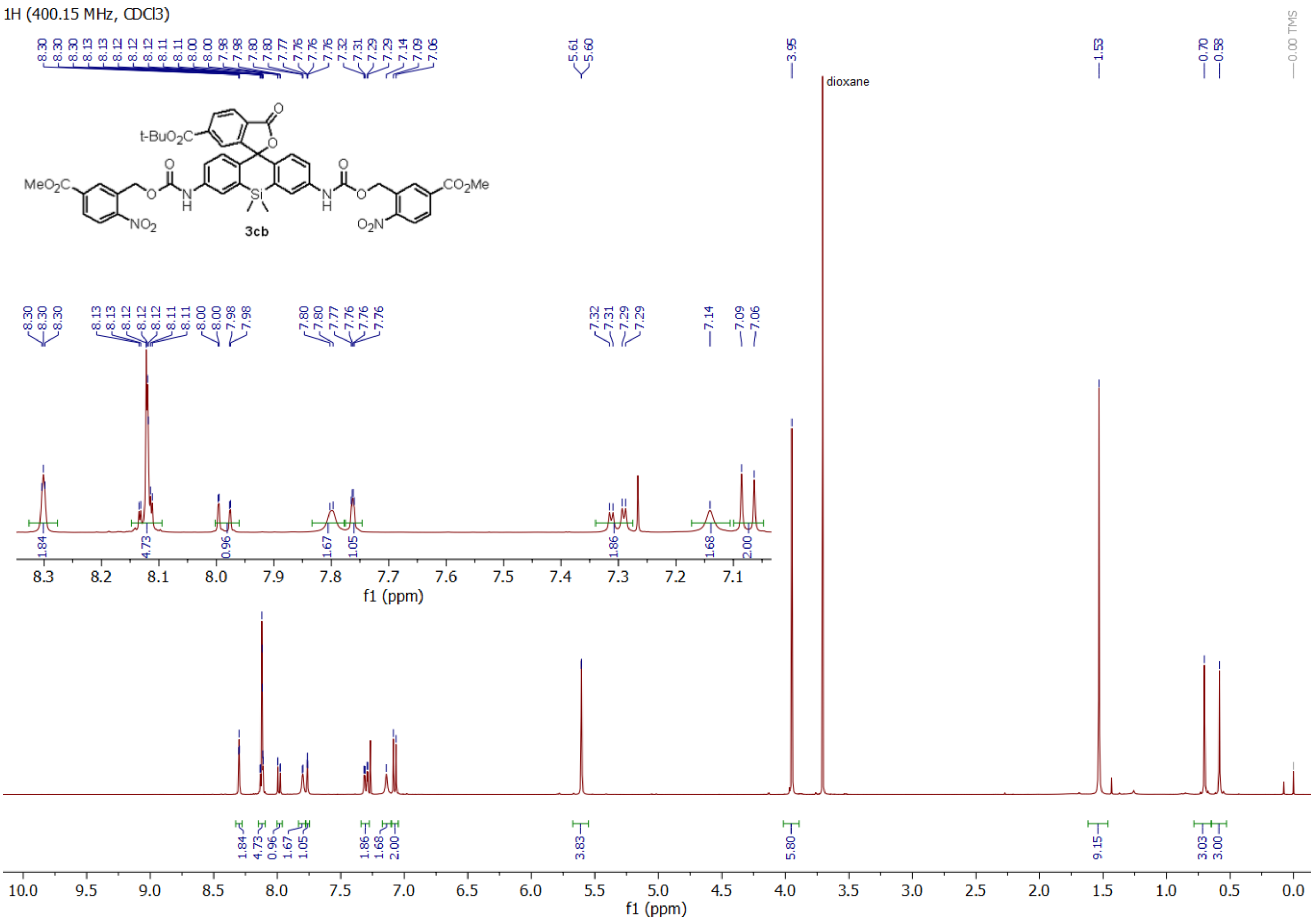
1H (400.15 MHz, CDCl3)
dioxane
t-BuO2C
MeO2C
CO2Me
NO2
O2N
Si
3cb
0.00 TMS
f1 (ppm)
f1 (ppm)

13C (100.63 MHz, CDCl3)

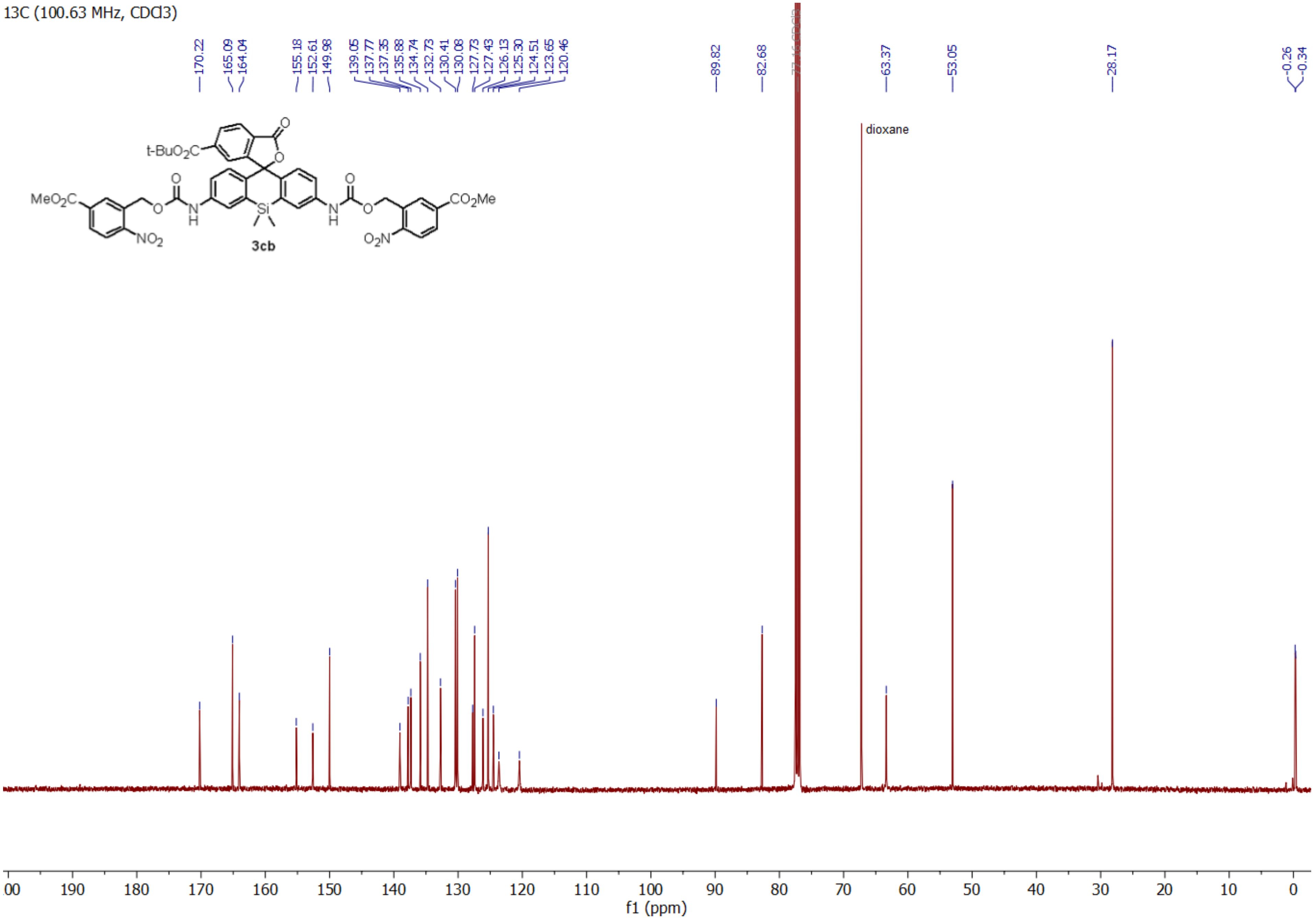

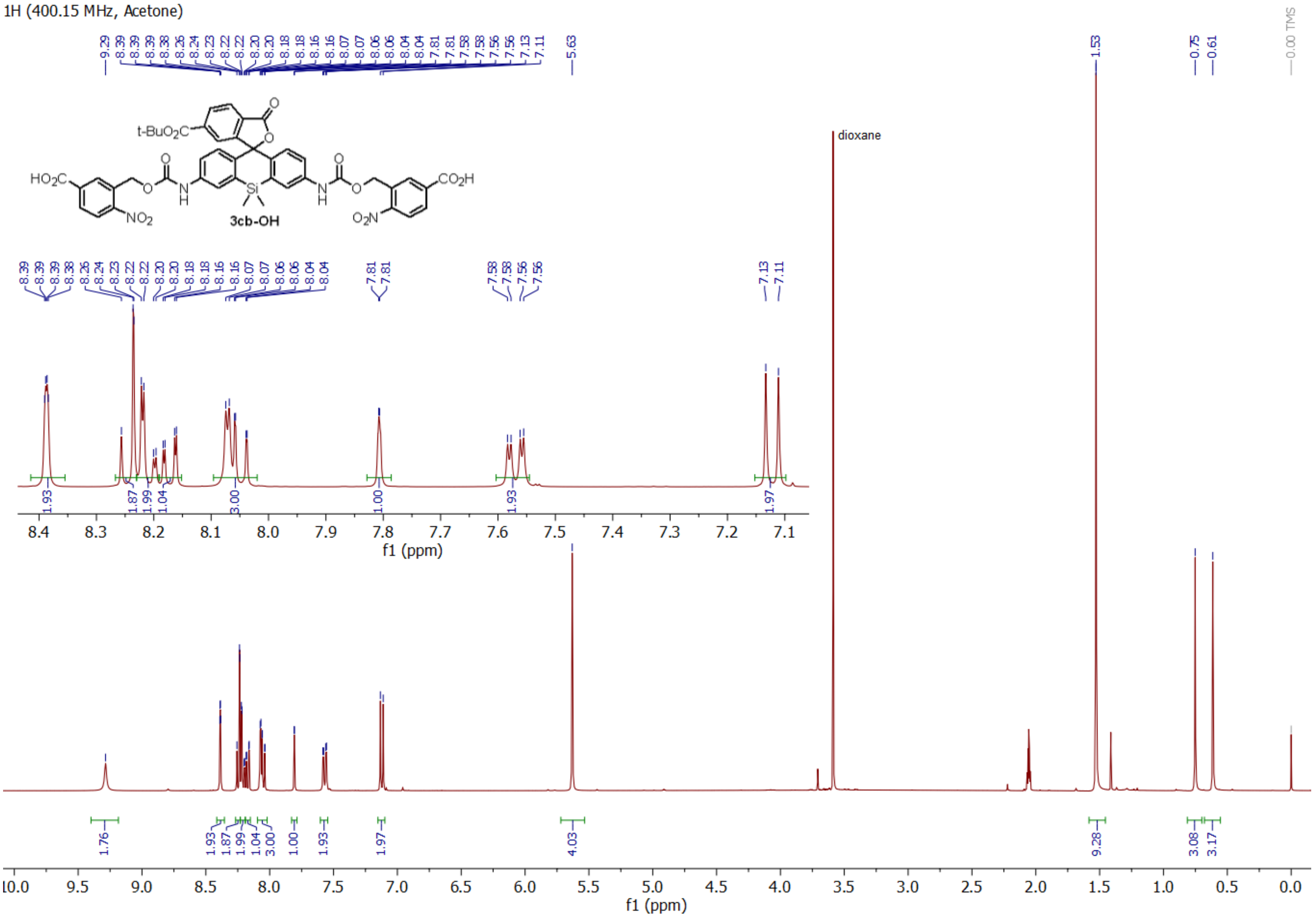
1H (400.15 MHz, Acetone)
9.29 8.39 8.39 8.39 8.38 8.26 8.24 8.23 8.22 8.22 8.20 8.20 8.18 8.18 8.16 8.16 8.07 8.07 8.06 8.06 8.04 8.04 7.81 7.81 7.58 7.58 7.56 7.56 7.13 7.11
5.63
1.53
0.75
0.61
0.00 TMS
t-BuO2C
HO2C
CO2H
NO2
O2N
3cb-OH
dioxane
1.76 1.93 1.87 1.99 1.04 3.00 1.00 1.93 1.97 4.03 9.28 3.08 3.17
f1 (ppm)

13C (100.63 MHz, Acetone)

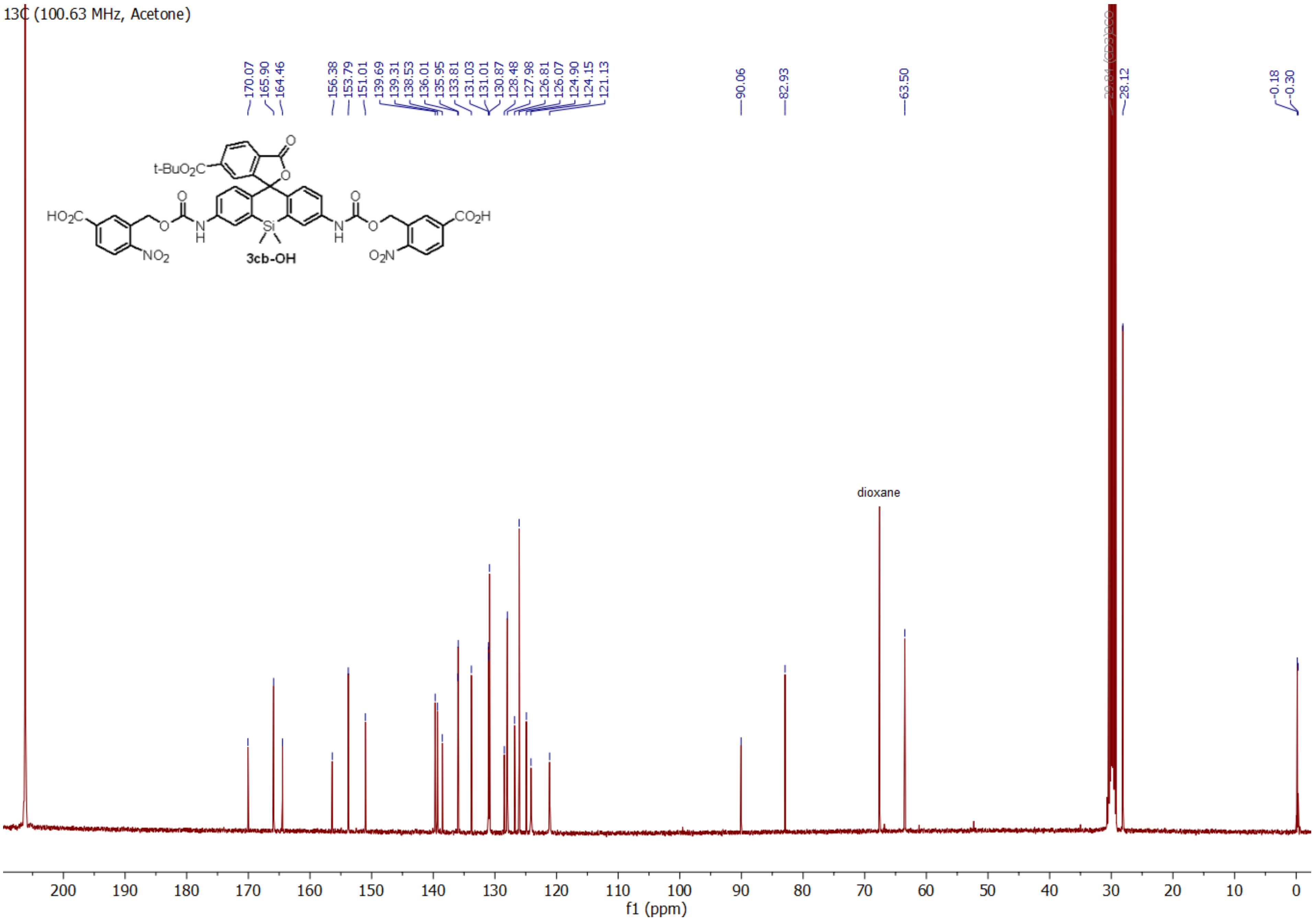

1H (400.15 MHz, MeOD)

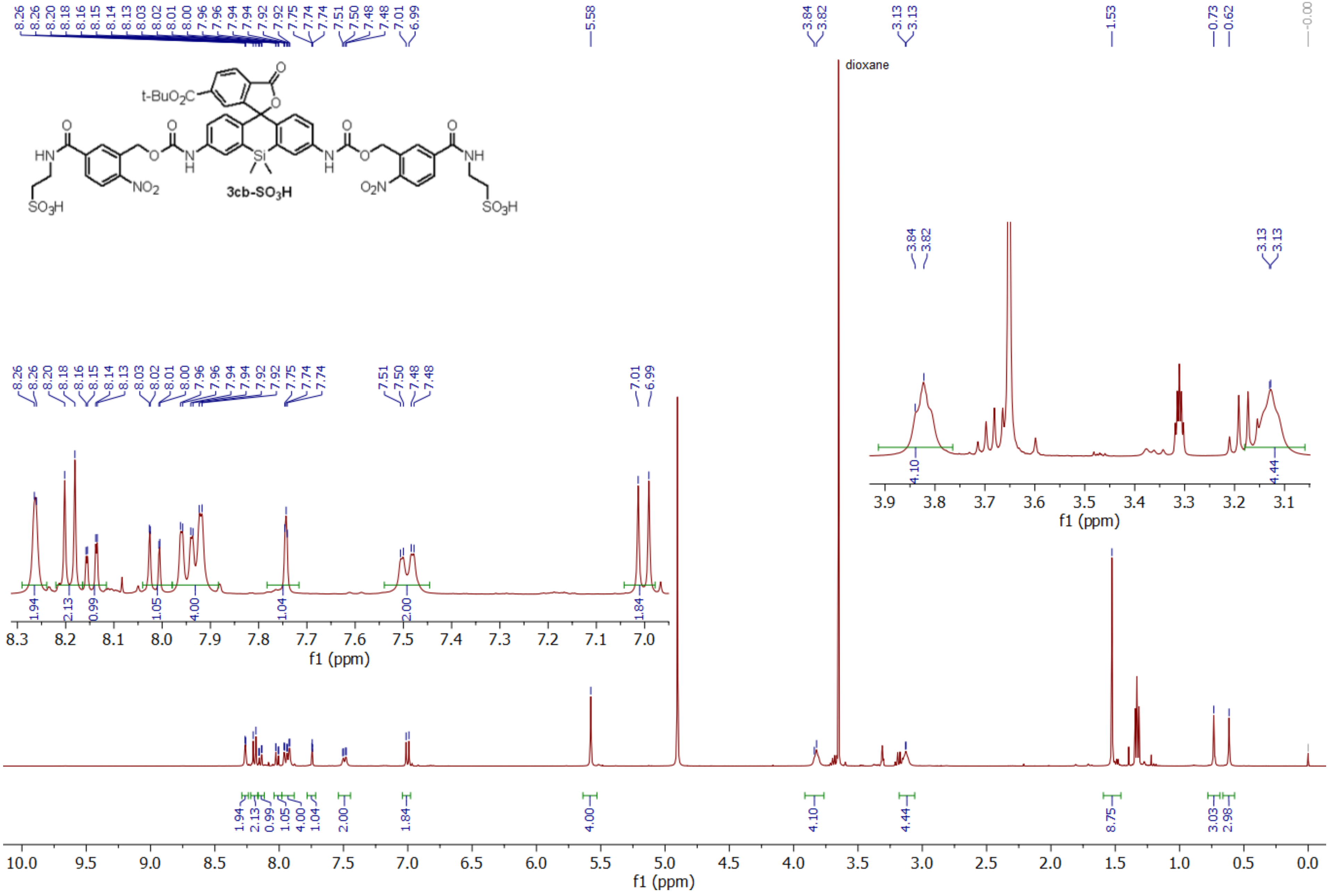

13C (100.63 MHz, MeOD)

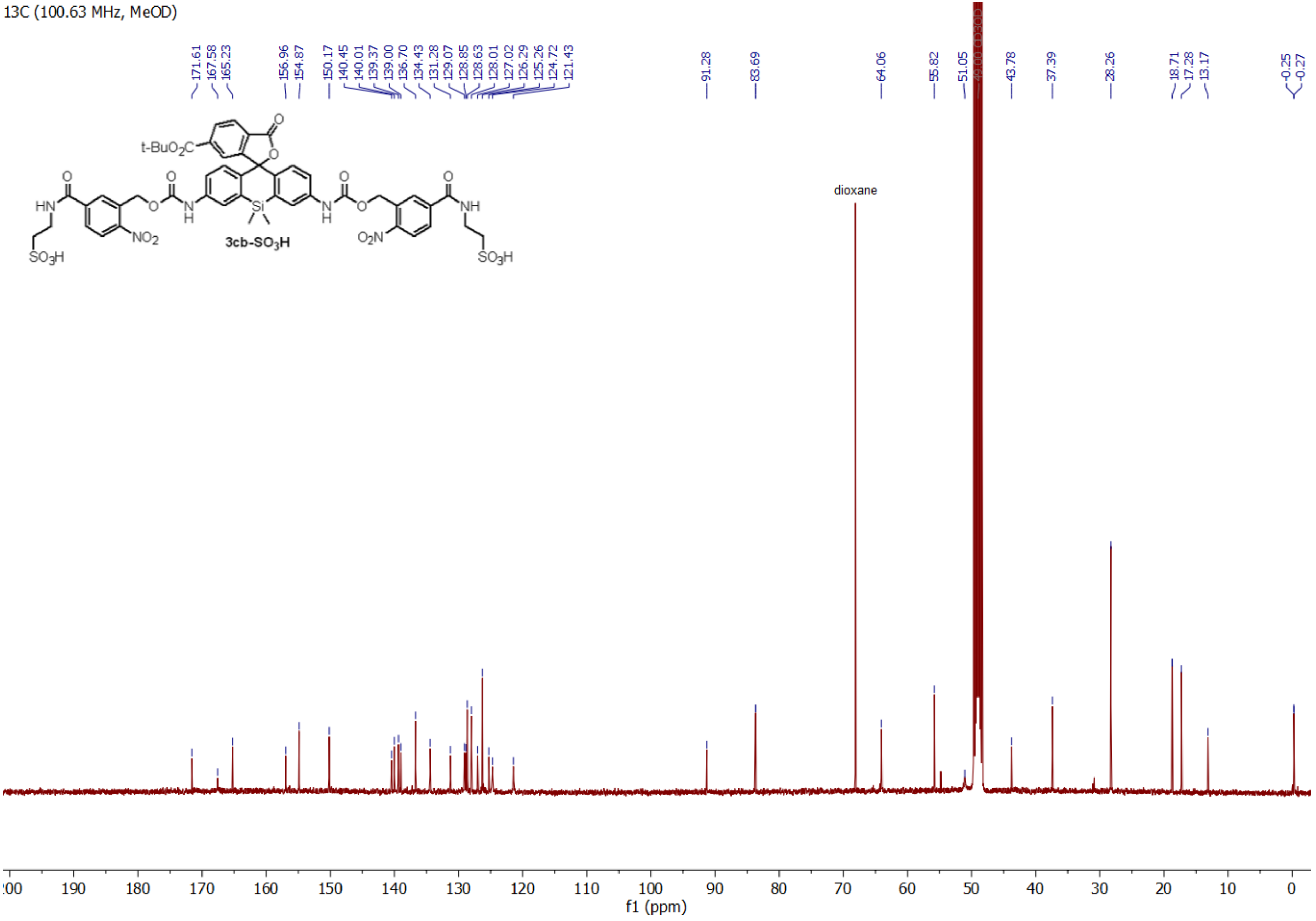

1H (400.15 MHz, DMSO)

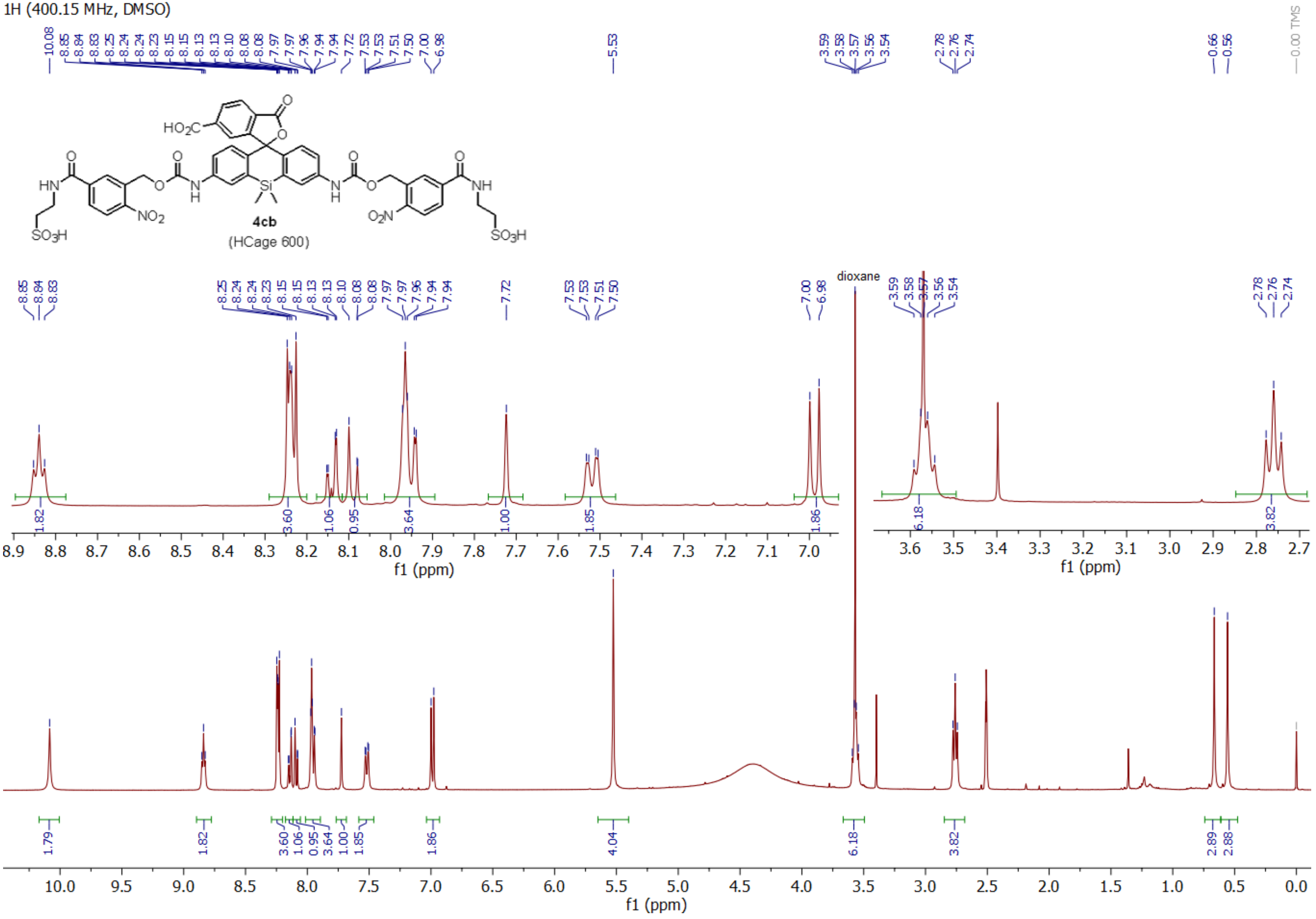

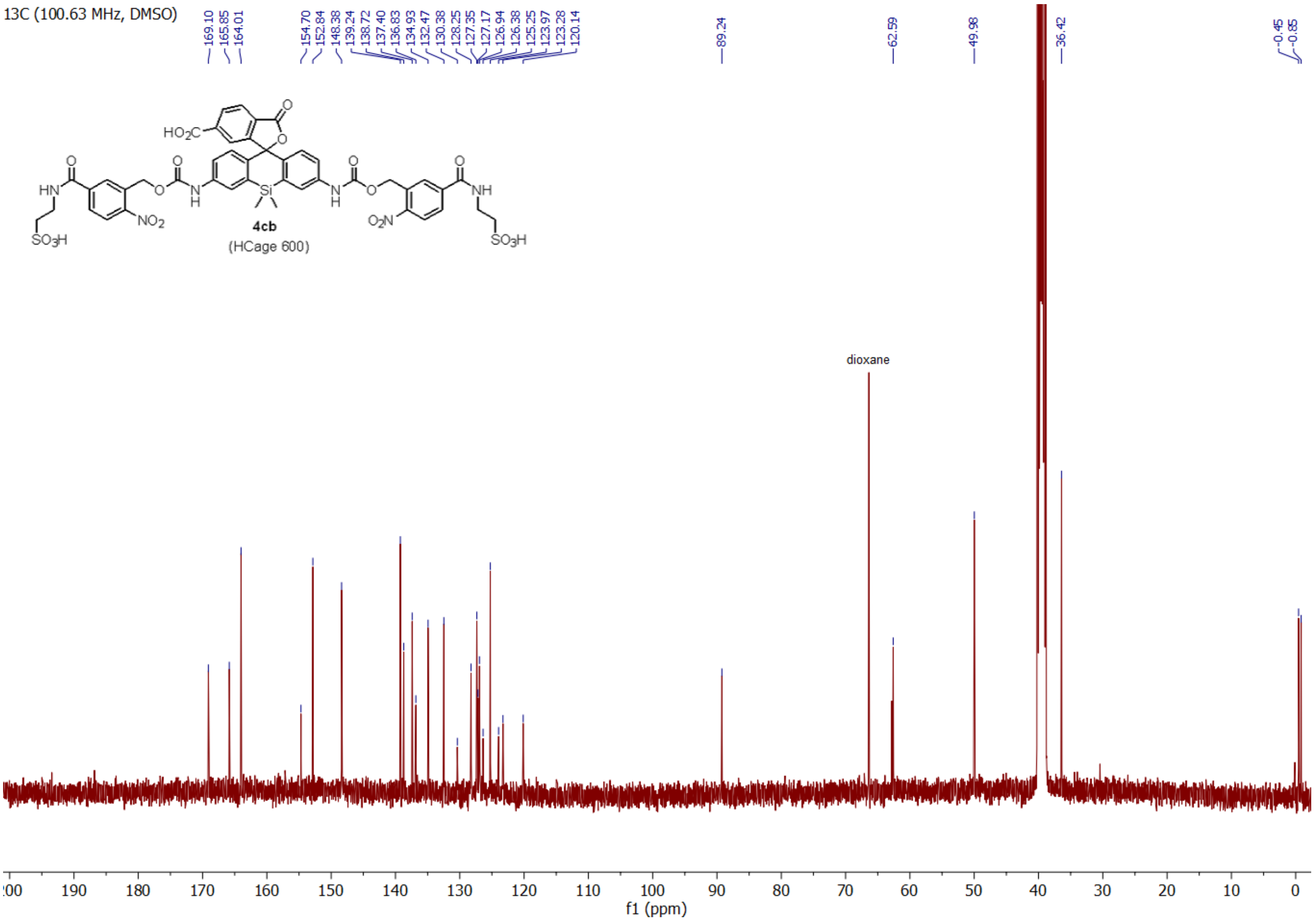

13C (100.63 MHz, DMSO)
169.10
165.85
164.01
154.70
152.84
148.38
139.24
138.72
137.40
136.83
134.93
132.47
130.38
128.25
127.35
127.17
126.94
126.38
125.25
123.97
123.28
120.14
89.24
62.59
49.98
36.42
-0.45
-0.85
4cb
(HCage 600)
dioxane
f1 (ppm)

1H (400.15 MHz, CDCl3)

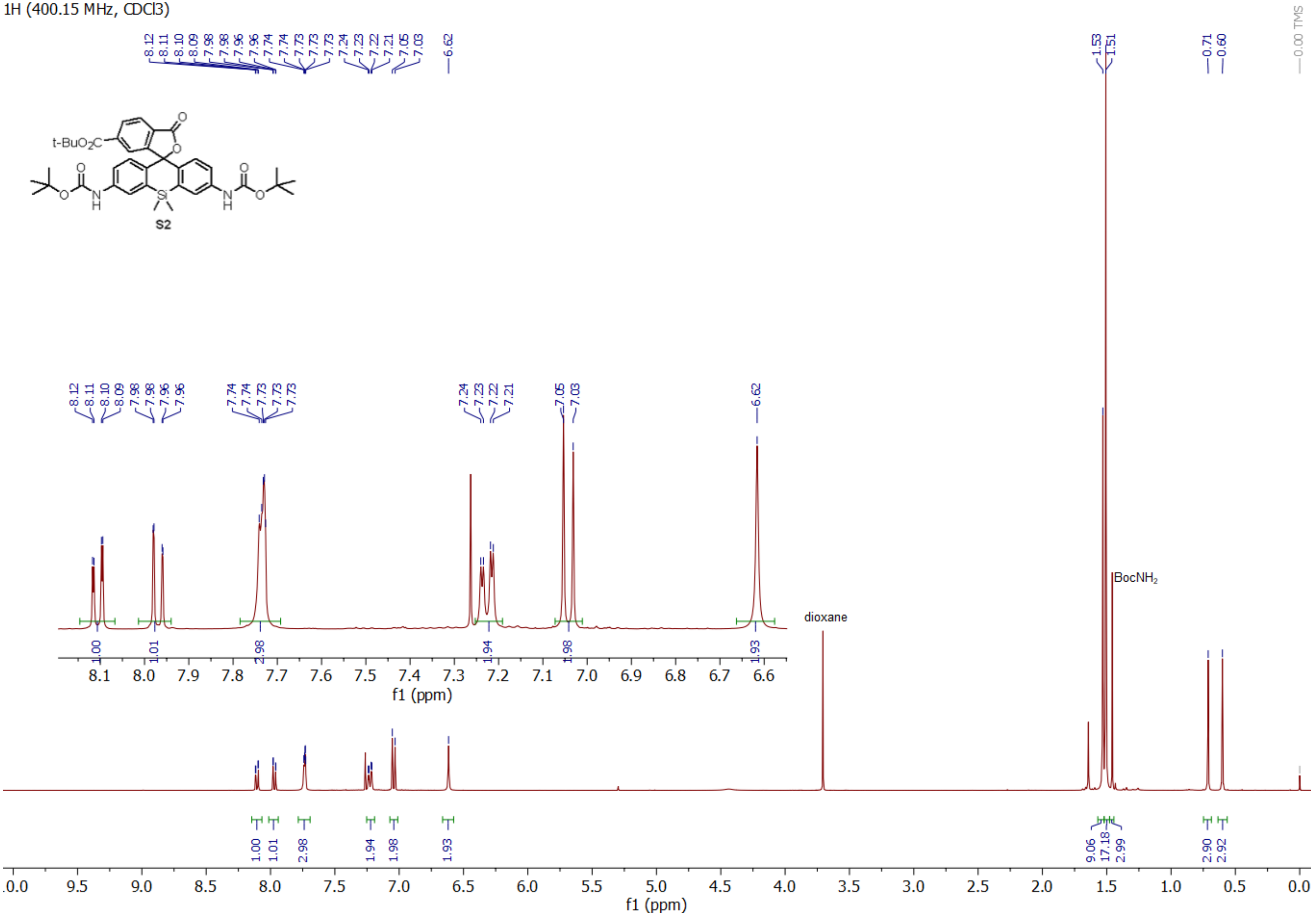

13C (100.63 MHz, CDCl3)

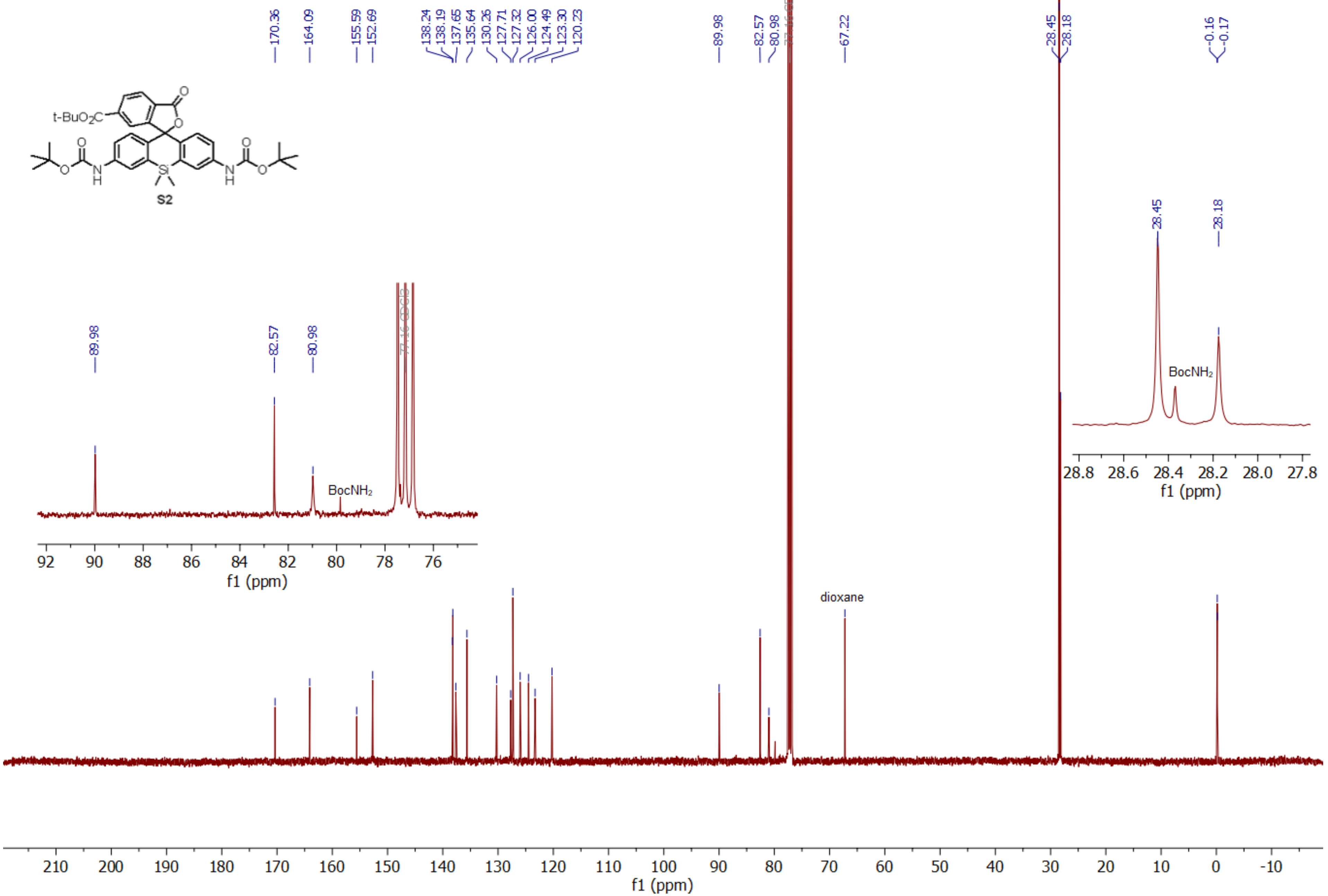

1H (400.15 MHz, CD3CN)

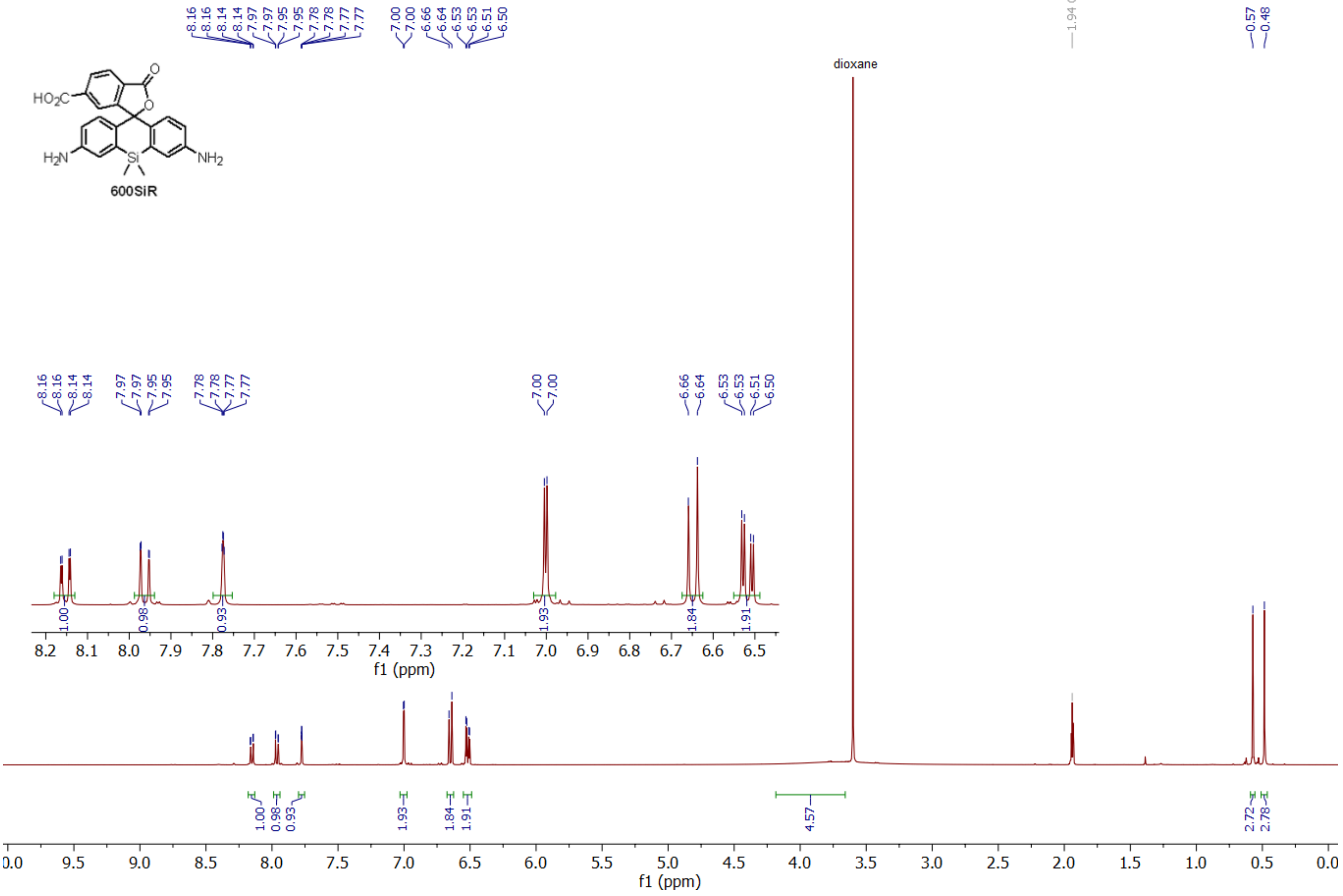

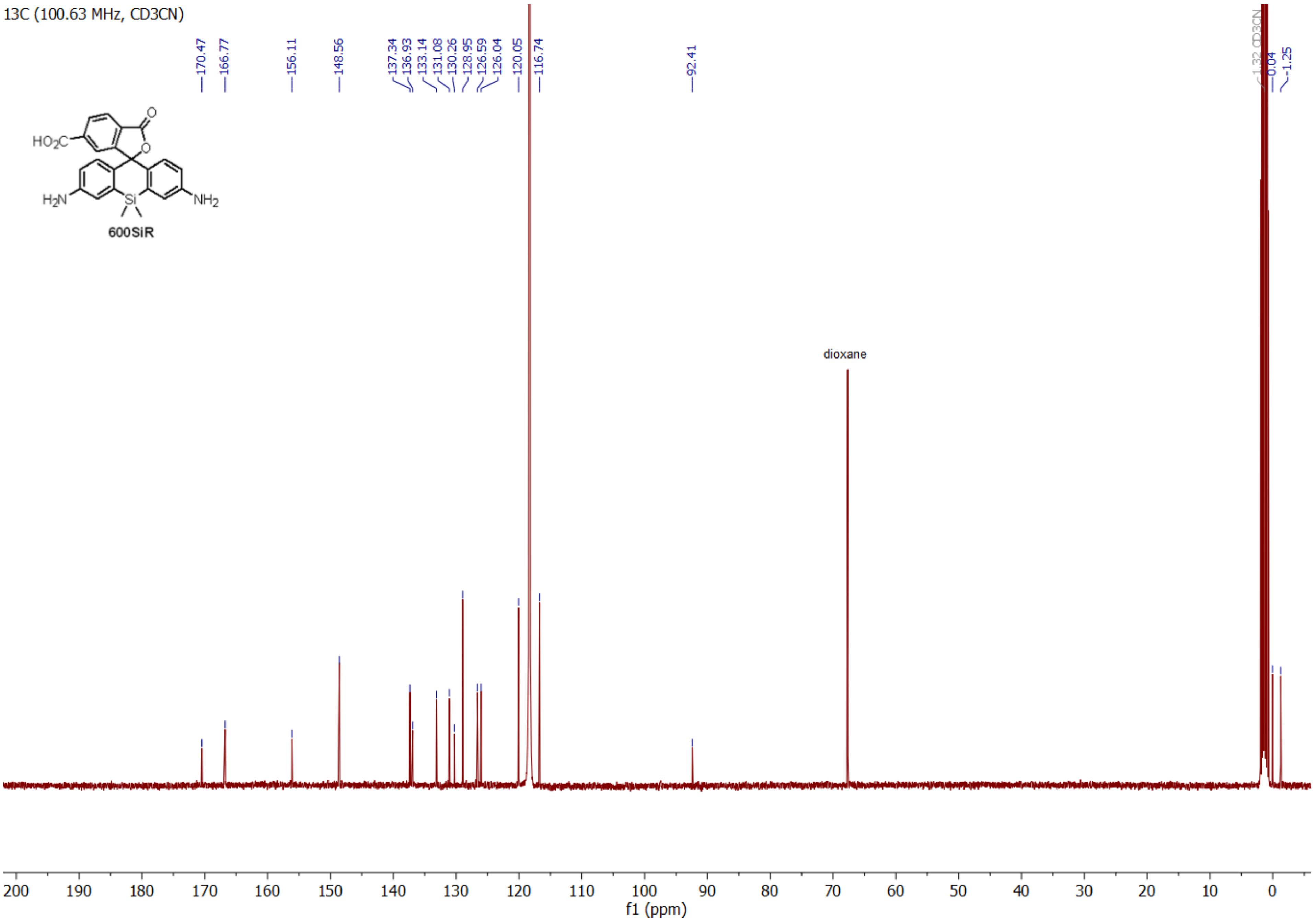
13C (100.63 MHz, CD3CN)
170.47
166.77
156.11
148.56
137.34
136.93
133.14
131.08
130.26
128.95
126.59
126.04
120.05
116.74
92.41
1.32 CD3CN
0.04
-1.25
dioxane
HO2C
H2N
NH2
Si
O
600SiR
f1 (ppm)
200
190
180
170
160
150
140
130
120
110
100
90
80
70
60
50
40
30
20
10
0
